# A Riemannian Geometry Theory of Three-Dimensional Binocular Visual Perception

**DOI:** 10.3390/vision2040043

**Published:** 2018-12-05

**Authors:** Peter D. Neilson, Megan D. Neilson, Robin T. Bye

**Affiliations:** 1 School of Electrical Engineering and Telecommunications, University of New South Wales, Sydney NSW 2052, Australia; 2 Independent Researcher, late School of Electrical Engineering and Telecommunications, University of New South Wales, Sydney NSW 2052, Australia; 3 Cyber-Physical Systems Laboratory, Department of ICT and Natural Sciences, NTNU—Norwegian University of Science and Technology, NO-6025 Ålesund, Norway

**Keywords:** visual space, Riemannian geometry, binocular vision, stereopsis, size perception, shape perception, place encoding, occlusions, computational model

## Abstract

We present a Riemannian geometry theory to examine the systematically warped geometry of perceived visual space attributable to the size–distance relationship of retinal images associated with the optics of the human eye. Starting with the notion of a vector field of retinal image features over cortical hypercolumns endowed with a metric compatible with that size–distance relationship, we use Riemannian geometry to construct a place-encoded theory of spatial representation within the human visual system. The theory draws on the concepts of geodesic spray fields, covariant derivatives, geodesics, Christoffel symbols, curvature tensors, vector bundles and fibre bundles to produce a neurally-feasible geometric theory of visuospatial memory. The characteristics of perceived 3D visual space are examined by means of a series of simulations around the egocentre. Perceptions of size and shape are elucidated by the geometry as are the removal of occlusions and the generation of 3D images of objects. Predictions of the theory are compared with experimental observations in the literature. We hold that the variety of reported geometries is accounted for by cognitive perturbations of the invariant physically-determined geometry derived here. When combined with previous description of the Riemannian geometry of human movement this work promises to account for the non-linear dynamical invertible visual-proprioceptive maps and selection of task-compatible movement synergies required for the planning and execution of visuomotor tasks.

## 1. Introduction

From the time of Euclid (300 BC) onwards builders and surveyors and the like have found the three-dimensional (3D) world in which they function to be adequately described by the theorems of Euclidean geometry. The shortest path between two points is a straight line, parallel lines never meet, the square on the hypotenuse equals the sum of squares on the other two sides, and so forth. When we look at the world about us we see a 3D world populated with 3D objects of various shapes and sizes. However, it is easy to show that what we see is a distorted or warped version of the Euclidean world that is actually out there. Hold the left and right index fingers close together about 10 cm in front of the eyes. They appear to be the same size. Fix the gaze on the right finger and move it out to arm’s length. The right finger now looks smaller than the left while, with the gaze fixed on the right finger, the left finger looks blurred and double. If the left finger is moved from side to side, neither of the double images occludes the smaller right finger; both images of the left finger appear transparent. The same phenomenon occurs regardless of which direction along the line of gaze the right finger is moved. Clearly, our binocular perception of 3D visual space is a distorted or warped version of the 3D Euclidean space actually out there.

Van Lier [1], in his thesis on visual perception in golf putting, reviewed many studies indicating that the perceived visual space is a warped version of the actual world. He likened this to the distorted image of the world reflected in a sphere as depicted in M C Escher’s 1935 lithograph “*hand with reflecting sphere*”. In Escher’s warped reflection straight lines have become curved, parallel lines are no longer parallel, and lengths and directions are altered. The exact nature of this warping can be defined by Riemannian geometry where the constant curvature of the sphere can be computed from the known Riemannian metric on a sphere. We mention Escher’s reflecting sphere simply to illustrate how the image of the 3D Euclidean world can be warped by the curvature of visual space. We are not claiming that the warped image of the Euclidean environment seen by humans is the same as a reflection in a sphere; indeed it is unlikely to be so. Nevertheless it provides a fitting analogy to introduce the Riemannian concept.

Luneburg [2] appears to have been first to argue that the geometry of the perceived visual space is best described as Riemannian with constant negative curvature. He showed that the geometry of any manifold can be derived from its metric which suggested that the problem is “*to establish a metric for the manifold of visual sensation*”. This is the approach we adopt here. We contend that the appropriate metric is that defined by the *size–distance relationship* introduced by the geometrical optics of the eye, as will be detailed later in this section. It is due to this relationship that objects are perceived to change in size without changing their infinitesimal shape as they recede along the line of gaze. A small ball, for example, appears to shrink in size as it recedes but it still looks like a ball. It does not appear to distort into an ellipsoid or a cube or any other shape. This gives an important clue to the geometry of 3D perceived visual space. For objects to appear to shrink in size without changing their infinitesimal shape as they recede along the line of gaze, the 3D perceived visual space has to shrink equally in all three dimensions as a function of Euclidean distance along the line of gaze. If it did not behave in this way objects would appear to shrink in size unequally in their perceived width, height and depth dimensions and, consequently, not only their size but also their infinitesimal shape would appear to change. (We use the term “infinitesimal shape” because, as explained in Section 4.1, differential shrinking in all three dimensions as a function of Euclidean distance causes a contraction in the perceived depth direction and so distorts the perceived shape of macroscopic objects in the depth direction as described by Gilinsky [3].)

The perceived change in size of objects causes profound distortion. Perceived depths, lengths, areas, volumes, angles, velocities and accelerations all are transformed from their true values. It is those transformations that are encapsulated in the metric from which we can define the warping of perceived visual space. The terms “perceived visual space” and “perceived visual manifold” occur throughout this paper and, since in Riemannian geometry a manifold is simply a special type of topological space, we apply these terms synonymously. Moreover, the use of “perceived” should be interpreted philosophically from the point of view of indirect realism as opposed to direct realism, something we touch on in Section 8. In other words, in our conceptualization, perceived visual space and the perceived visual manifold are expressions of the neural processing and mapping that form the physical representation of visual perception in the brain.

Since at least the eighteenth century, philosophers, artists and scientists have theorized on the nature of perceived visual space and various geometries have been proposed [4,5]. Beyond the simple demonstration above there has long been a wealth of formal experimental evidence to demonstrate that what we perceive is a warped transformation of physical space [2,3,6,7,8,9,10,11,12,13,14]. In some cases a Riemannian model has seemed appropriate but, as we shall see from recent considerations, the mathematics of the distorted transformation is currently thought to depend on the experiment. Prominent in the field has been the work of Koenderink and colleagues, who were first to make direct measurements of the curvature of the horizontal plane in perceived visual space using the novel method of *exocentric pointing* [15,16,17]. Results showed large errors in the direction of pointing that varied systematically from veridical, depending on the exocentric locations of the pointer and the target. The curvature of the horizontal plane derived from these data revealed that the horizontal plane in perceived visual space is positively curved in the near zone and negatively curved in the far zone. Using alternative tasks requiring judgements of *parallelity* [18] and *collinearity* [19] this same group further measured the warping of the horizontal plane in perceived visual space. Results from the collinearity experiment were similar to those from the previous exocentric pointing experiment but, compared with the parallelity results, the deviations from veridical had a different pattern of variation and were much smaller.

Using the parallelity data, Cuijpers et al. [20] derived the Riemannian metric and the Christoffel symbols for the perceived horizontal plane. They found that the Riemannian metric for the horizontal plane was *conformal*, that is, the angles between vectors defined by the metric are equal to the angles defined by a Euclidean metric. They computed the components $R_{jkl}^{i}$ of the curvature tensor and found them to be zero (i.e., flat) for every point in the horizontal plane. This was not consistent with their finding of both positive and negative curvature in the earlier pointing experiment. Meanwhile, despite the similarity of the experimental setups, these authors had concluded that their collinearity results could not be described by the same Riemannian geometry that applied to their parallelity results [19]. The implication was that the geometry of the perceived visual space is task-dependent. From the continued work of this group [21,22,23,24,25,26,27,28,29,30,31] along with that of other researchers, it is now apparent that experimental measures of the geometry of perceived visual space are not just task-dependent. They vary according to the many contextual factors that affect the spatial judgements that provide those measures [4,5,32]. Along with the nature of the task these can include what is contained in the visual stimuli, the availability of external reference frames, the setting (indoors vs. outdoors), cue conditions, judgement methods, instructions, observer variables such as age, and the presence of illusions.

The inconsistency of results in the many attempts to measure perceptual visual space has led some to question or even abandon the concept of such a space [19,33]. This is unnecessary. Wagner and Gambino [4] draw attention to researchers who argue that there really is only one visual space in our perceptual experience but that it has a cognitive overlay in which observers supplement perception with their knowledge of how distance affects size [11,34,35,36,37,38,39]. We agree. However, Wagner [32] argues that separation into sensory and cognitive components is meaningless unless the sensory component is reportable under some experimental condition. While there is, unfortunately, no unambiguous way to determine such a condition, mathematical models and simulations of sensory processes may provide a possible way around the dilemma. Rather than rejecting the existence of a geometrically invariant perceived visual space we suggest that the various measured geometries are accounted for by top-down cognitive mechanisms perturbing the underlying invariant geometry derivable mathematically from the size–distance relationship between the size of the image on the retina and the Euclidean distance between the nodal point of the eye and the object in the environment. This relationship is attributable to the anatomy of the human eye functioning as an optical system. For simplicity in what follows we will refer to this size–distance relationship as being attributable to the geometric optics of the eye. We now consider what determines that geometry.

The human visual system has evolved to take advantage of a frontal-looking, high-acuity central (foveal), low-acuity peripheral, binocular anatomy but at the same time it has had to cope with the inevitable size–distance relationship of retinal images that the geometric optics of such eyes impose. To allow survival in a changing and uncertain 3D Euclidean environment it would seem important for the visual system to have evolved so that the perceived 3D visual space matches as closely as possible the Euclidean structure of the actual 3D world. We contend that in order to achieve this, the visual system has to model the ever-present warping introduced by the geometrical optics of the eye and that this warping can be described by an invariant Riemannian geometry. Accordingly, this paper focuses on that geometry and on the way it can be incorporated into a realistic neural substrate.

A simple pinhole camera model of the human eye [40,41] shows that the size of the image on the retina of an object in the environment changes in inverse proportion to the Euclidean distance between the pinhole and the object in the environment. Modern schematic models of the eye are far more complex, with multiple refracting surfaces needed to emulate a full range of optical characteristics. However, as set out by Katz and Kruger [42], object–image relationships can be determined by simple calculations using the optics of the reduced human eye due to Listing. They state:

[Listing] reduced the eye model to a single refracting surface, the vertex of which corresponds to the principal plane and the nodal point of which lies at the centre of curvature. The justification for this model is that the two principal points that lie midway in the anterior chamber are separated only by a fraction of a millimetre and hardly shift during accommodation. Similarly, the two nodal points lie equally close together and remain fixed near the posterior surface of the lens. In the reduced model the two principal points and the two nodal points are combined into a single principal point and a single nodal point. Retinal image sizes may be determined very easily because the nodal point is at the centre of curvature of this single refractory surface. A ray from the tip of an object directed toward the nodal point will go straight to the retina without bending, therefore object and image subtend the same angle. The retinal image size is found by multiplying the distance from the nodal point to the retina (17.2 mm) by the angle in radians subtended by the object [42] (see Figure 18).

Thus the geometry of the eye determines that the size of the retinal image varies in proportion to the angle subtended by the object at the nodal point of the eye. Or stated equivalently, the geometry of the eye determines that the size of the image changes in inverse proportion to the Euclidean distance between the object and the nodal point of the eye. For the perceived sizes of objects in the perceived 3D visual space to change in inverse proportion to Euclidean distance along the line of gaze in the outside world without changing their perceived infinitesimal shape, the perceived 3D visual space has to shrink by equal amounts in all three dimensions in inverse proportion to the Euclidean distance. From this assertion, the Riemannian metric for the 3D perceived visual space can be deduced, and from the metric the geometry of the 3D perceived visual space can be computed and compared with the geometry measured experimentally.

Our principal aim is to present a mathematical theory of the information processing required within the human brain to account for the ability to form 3D images of the outside world as we move about within that world. As such, the theory developed is about the computational processes and not about the neural circuits that implement those computations. Nevertheless, the theory builds on established knowledge of the visual cortex (Section 2) as well as on the existence of *place maps* that have been shown to exist in hippocampal and parahippocampal regions of the brain [43,44,45,46,47,48,49,50]. Throughout this paper we take it as given that the *place* and *orientation* of the head, measured with respect to an external Cartesian reference frame (X,Y,Z), are encoded by neural activity in hippocampal and parahippocampal regions of the brain and that this region acts as a portal into visuospatial memory. We focus on the computational processes required within the brain to form a cognitive model of the 3D visual world experienced when moving about within that world. In that sense, the resulting Riemannian theory can be seen as an extension of the view-based theory of spatial representation in human vision proposed by Glennerster and colleagues [51].

The theory is presented in a series of steps starting in the periphery and moving centrally as described in Section 2 through Section 7. To provide a road map and to illustrate how the various sections relate to each other, we provide here a brief overview.

Section 2: It is well known that images on the retinas are encoded into neural activity by photoreceptors and transmitted via retinal ganglion cells and cells in the lateral geniculate nuclei to cortical columns (hypercolumns) in the primary visual cortex. We define left and right retinal hyperfields and hypercolumns and describe the retinotopic connections between them. We treat hyperfields and hypercolumns as basic modules of image processing. We describe extraction of orthogonal features of images on corresponding left and right retinal hyperfields during each interval of fixed gaze by minicolumns within each hypercolumn. We further describe how the coordinates of image points in the 3D environment projecting on to left and right retinal hyperfields can be computed stereoscopically and encoded within each hypercolumn.

Section 3: Here we describe a means of accumulating an overall image of the environment seen from a fixed place. This depends on visual scanning of the environment via a sequence of fixed gaze points. We argue that at the end of each interval of fixed gaze, before the gaze is shifted and the information within the hypercolumns lost, the vectors of corresponding left and right retinal hyperfield image features encoded within each hypercolumn are pasted into a visuospatial gaze-based association memory network (*G*-memory) in association with their cyclopean coordinates. The resulting gaze-based *G*-memory forms an internal representation of the perceived 3D outside world with each ‘memory site’ accessed by the cyclopean coordinates of the corresponding image point in the 3D outside world. The 3D *G*-space with image-point vectors stored in vector-spaces at each image point in the *G*-space has the structure of a *vector bundle* in Riemannian (or affine) geometry. We also contend in Section 3 that the nervous system can adaptively model the *size–distance relationship* between the size of the 2D retinal image on the fovea and the Euclidean distance in the outside world between the eye and the object. From this relationship the *Riemannian metric* g(r, θ, φ) describing the perceived size of an object at each image point (r, θ, φ) in the environment can be deduced and stored at the corresponding image point (r, θ, φ) in G-memory. The *G*-memory is then represented geometrically as a 3D Riemannian manifold (G*,*g) with metric tensor field g. The geometry of the 3D perceived visual manifold can be computed from the way the metric g(r, θ, φ) varies from image point to image point in the manifold.

Section 4: Applying the metric deduced in Section 3, we use Riemannian geometry to quantify the warped geometry of the 3D perceived visual space.

Section 5: By simulating families of geodesic trajectories we illustrate the warping of the perceived visual space relative to the Euclidean world.

Section 6: Here we describe the computations involved in perceiving the size and shape of objects in the environment. When viewing from a fixed place, occlusions restrict us to seeing only curved 2D patches on the surfaces of 3D objects in the environment. The perceived 2D surfaces can be regarded as 2D submanifolds with boundary embedded in the ambient 3D perceived visual manifold (G*,*g). We show how the size and shape of the submanifolds can be computed using Riemannian geometry. In particular, we compute the way the warped geometry of the 3D ambient perceived visual manifold (G*,*g) causes the perceived size and shape of embedded surfaces to change as a function of position and orientation of the object in the environment relative to the observer.

Section 7: This introduces the notion of place-encoded visual images represented geometrically by a structure in Riemannian geometry known as a *fibre bundle*. We propose that the place of the head in the environment, encoded by neural activity in the hippocampus, is represented geometrically as a point p in a 3D base manifold P called the *place map*. Each point p in the base manifold P acts as an accession code for a partition of visuospatial memory (i.e., a vector bundle) $\left(G_{p},g\right)$. As the person moves about in the local environment, the vectors of visual features acquired through visual scanning at each place p are stored into the vector bundle $\left(G_{p},g\right)$ accessed by the point (place) p in the base manifold P. Thus place-encoded images of the surfaces of objects as seen from different places in the environment are accumulated over time in different vector bundle partitions $\left(G_{p},g\right)$ of visuospatial memory. We show that adaptively tuned maps between each and every partition (vector bundle) $\left(G_{p},g\right)$ of visuospatial memory, known in Riemannian geometry as *vector-bundle morphisms*, can remove occlusions and generate a 3D cognitive model of the local environment as seen in the correct perspective from any place in the environment.

Section 8: In this discussion section, material in the previous sections is pulled together and compared with experimental findings and with other theories of visuospatial representation.

Section 9: This section points to areas for future research development.

Riemannian geometry is concerned with curved spaces and the calculus of processes taking place within those curved spaces. This, we claim, is the best computational approach for analysis of visual processes within the curved geometry of 3D perceived visual space. A Riemannian geometry approach will reveal novel aspects of visual processing and allow the theory of 3D visual perception to be expressed in the language of modern mathematical physics. Our intention is to develop the groundwork for a theory of visual information processing able to account for the non-linearities and dynamics involved. The resulting theory will be integrated with our previous theory on the Riemannian geometry of human movement [52] but in this paper we focus entirely on vision. We do not attempt to justify in a rigorous fashion the theorems and propositions that we draw from Riemannian geometry. For that we rely on several excellent texts on the subject and we direct readers to these at the appropriate places. However, for those unfamiliar with the mathematics involved, we have sought to provide intuitive descriptions of the geometrical concepts. We trust that these, together with similar descriptions in our previous paper [52], can assist in making the power and the elegance of this remarkable geometry accessible to an interdisciplinary readership.

## 2. Preliminaries

Constructing a Riemannian geometry theory of visuospatial representation requires us to build bridges between well-established elements in the known science of the visual system and abstract objects in the Riemannian geometry of curved spaces. These bridges can be taken as definitions that link the real-world structure of the visual system with the abstract but deductively logical structure of Riemannian geometry. Thus, the descriptions in this section are crafted in a form that facilitates the application of Riemannian geometry to visual processing. While these bridges are clearly important, we see them as preliminary and not the main focus of the geometrical theory developed in subsequent sections. Thus we give only abbreviated description of these preliminaries, and rely on the reader to refer as needed to texts such as *Seeing. The Computational Approach to Biological Vision* [40], *Perceiving in Depth* [53,54,55], *Sensation and Perception* [56] and papers referenced therein.

Section 2.1, Section 2.2, Section 2.3, Section 2.4, Section 2.5, Section 2.6 and Section 2.7 outline proposals concerning binocular vision and the encoding of retinal hyperfield images by the hypercolumns of V1. We define corresponding left and right retinal hyperfields and the cortical hypercolumn to which they connect as the basic computational module for extracting visual image features during an interval of fixed gaze. We propose that the parallel processing of many such modules provides the structure of vector fields over a Riemannian manifold. Then, in Section 2.8, Section 2.9 and Section 2.10, we address mechanisms of depth perception and the computation of cyclopean coordinates that provide the Riemannian manifold upon which the vector fields of image features sit.

### 2.1. Retinal Coordinates

The straight-line ray passing through the nodal point of the eye and impinging on the centre of the fovea is called the visual axis of the eye. The horizontal angle $\hat{\theta }$ and the vertical angle $\hat{\varphi }$ measured relative to the visual axis of all other straight-line rays passing through the nodal point of the eye and impinging on the retina define a set of coordinates ($\hat{\theta }$,$\hat{\varphi }$) on the retina. The angles ($\hat{\theta }$,$\hat{\varphi }$) can be related to the homogeneous coordinates of the real projective plane $RP^{2}$ defined in Riemannian geometry [57] but we do not explore this further here. For simplicity, we refer to the open subset of the projective plane corresponding to all the straight-line rays passing through the nodal point and impinging on the retina as the *retinal plane* and we use the angles ($\hat{\theta }$,$\hat{\varphi }$) as a coordinate system for points on this retinal plane. We use the ‘hat’ notation to distinguish the retinal coordinates ($\hat{\theta }$,$\hat{\varphi }$) from the coordinates (θ, φ) used later to represent direction of gaze. We define *corresponding points* in the left and right retinas to be points having the same retinal coordinates ($\hat{\theta }$,$\;\hat{\varphi }$). Retinal coordinates are important because, via projective geometry, they provide the only link between events in the Euclidean outside world with neural encoding of those visual events within the nervous system.

An advantage of using angles ($\hat{\theta }$,$\hat{\;\varphi }$) as coordinates for each retina is that, as mentioned in Section 1, the size of the image on the retina of an object in the environment is proportional to the angle subtended by the object at the nodal point of the eye. We claim that the *perceived* size of an object in the 3D environment is also proportional to the angle subtended by the object at the nodal point of the eye. Later we will show that this is a property of the Riemannian geometry of the perceived visual space.

### 2.2. Hyperfields

A *hyperfield* is a collection of overlapping ganglion cell receptive fields in the retina. We assume that the number of ganglion cells making up a hyperfield varies from small (≈25) in the fovea to large (300–500) in the periphery. For descriptive simplicity we assume that a hyperfield typically involves about 100–200 overlapping ganglion cell receptive fields. The instantaneous frequency of ganglion-cell action potentials is modulated by the Laplacian ΔΦ, sometimes called edge detection, of the intensity Φ($\hat{\theta }$,$\hat{\;\varphi }$) of light across the ganglion cell receptive field [58]. The response of a hyperfield to the spatial pattern of light Φ($\hat{\theta }$,$\hat{\varphi }$) falling on the hyperfield is encoded by the temporospatial pattern of action potentials of the collection of ganglion cells whose overlapping ganglion cell receptive fields make up the hyperfield.

### 2.3. Retinotopic Connections between Hyperfields and Hypercolumns

Ganglion cells from *corresponding hyperfields* in the left and right retinas (i.e., hyperfields centred on the same retinal coordinates ($\hat{\theta }$,$\hat{\;\varphi }$) in the left and right retinas) project in a retinotopic fashion via the lateral geniculate nucleus (LGN) to local clusters of cortical columns in V1. These local clusters of about 100–200 minicolumns can be defined as *hypercolumns* [40]. Each pair of nearby hyperfields in each retina is mapped to nearby hypercolumns in V1. Importantly, corresponding hyperfields in the left and right retinas map to the same hypercolumn in V1. Stimulation of a left-eye retinal hyperfield activates a subset of minicolumns within a hypercolumn while stimulation of the corresponding right-eye hyperfield activates another overlapping subset of minicolumns in the same hypercolumn. Thus we can define *left ocular dominance minicolumns, right ocular dominance minicolumns,* and *binocular minicolumns* within a hypercolumn.

### 2.4. Hypercolumns

We regard each hypercolumn in V1 together with the corresponding left and right retinal hyperfields connecting to it as a functional visual processing module. The response of any single cortical cell is too ambiguous for it to serve as a reliable feature detector on its own [40]. Instead, we see minicolumns within a hypercolumn as the feature detectors for the different spatial patterns of light I($\hat{\theta }$, $\hat{\varphi }$) falling during a fixed-gaze interval on corresponding left and right retinal hyperfields that project retinotopically to the hypercolumn. This is consistent with long-established work concerning (i) the functional organization of the neocortex into a columnar arrangement [59,60,61,62]; (ii) the reciprocal columnar organization between the thalamus and the cerebral cortex [63]; (iii) the existence of networks of interconnected columns within widely separated regions of the cortex [64]; and (iv) the computational modelling of cortical columns [65,66,67]. Microelectrode recordings as well as other methods for visualizing the activity of cortical columns in V1 show that cells within a cortical column respond to the same feature of the light pattern on the retina. This has led to terms such as *orientation columns, ocular dominance columns, binocular columns* and *color blobs.*

### 2.5. Visual Features Extracted by Cortical Columns

The synaptic connections to cortical columns in V1 are well known to be plastic. They tune slowly over several weeks [68,69,70]. The features extracted by minicolumns in V1 are, therefore, stochastic features averaged over the tens of thousands of different patterns of light falling on the retinal hyperfields over the several weeks needed to tune the synaptic weights.

The stochastic properties of natural scenes averaged over long time windows possess statistical regularities at multiple scales [71,72]. Amplitude spectra averaged over tens of thousands of natural images are maximal at low spatial frequencies and decrease linearly with increasing frequency. This reveals the presence of correlations between neighbouring points in the images that persist in the averaged image [71]. Recent studies have revealed that micro movements of the head and eyes during each interval of fixed gaze modify the averaged spectra. The resulting averaged amplitude spectra are flat up to a spatial frequency of about 10 cycles per degree, after which they decrease rapidly with increasing spatial frequency [72,73,74].

While this information is valuable, it depends on linear analysis where amplitude spectra are derived from second-order statistics without taking higher-order moments into account. Yet the probability distribution of natural scenes is known to be non-Gaussian, with the stochastic structure revealing the existence of persistent higher-order moments [75,76]. The visual system, therefore, has to deal with these non-linearities. It is established [71,75,77,78] that the behaviour of simple cells in V1 can be described by Gabor functions [79] whose responses extend in both spatial and temporal frequency. Indeed, Olshausen and Field [77] have shown that maximizing the non-Gaussianity (sparseness) of image components is enough to explain the emergence of Gabor-like filters resembling the receptive fields of simple cells in V1. Likewise, Hyvӓrinen and Hoyer [80,81] have shown that the technique of *independent components analysis* can explain the emergence of invariant features characteristic of both simple and complex cells in V1. In reviewing the statistics of natural images and the processes by which they might efficiently be neurally encoded, Simoncelli and Olshausen [76] discussed independent components analysis as equivalent to a two-stage process involving first a linear principal components decomposition followed by a second rotation to take non-Gaussianity (non-linearity) into account. Our description of non-linear singular value decomposition as a two-stage process for visual feature extraction (Section 2.7, Appendix A) parallels the two-stage process described by Simoncelli and Olshausen [76].

### 2.6. Gaze and Focus Control

High acuity vision derives from the foveal region of the retina where the density of photoreceptors is greatest (≈160,000 cones per sq mm). The central region of the foveal pit is only about 100 microns across and subtends an angle of only about 0.3 degrees [40] while the rod-free fovea covers only 1.7 degrees of the visual field leaving 99.9% in the periphery [82]. Consequently, with the gaze fixed, it is only possible to obtain a high resolution image for a relatively small patch centred about the gaze point on the surface of an object in the environment. To build a high resolution image of the environment as seen from a fixed place the gaze has to be shifted from point to point via a sequence of head rotations and/or saccades with fixations of the eyes allowing the left and right retinal images for each gaze point to be accumulated in visuospatial memory. The *interval of fixed gaze* can vary from as short as 100 ms up to many thousands of milliseconds.

To perform such visual scanning, the nervous system requires a precision movement control system able to control the place and orientation of the head in the environment as well as the rotation of the eyes in the head and the thicknesses of the lenses in the eyes. Given a required gaze point in the environment, the *gaze control system* has to plan and execute coordinated movements of the head and eyes. Once the gaze point has been acquired the control system has to hold the images of the gaze point fixed on the foveas. Simultaneously the *focus control system* has to adjust the thicknesses of the lenses to maximize the sharpness of the images on the foveas.

Eye-movement control has *wired-in* synergies for conjugate, vergence, vertical and roll movements of the two eyes in the head and has a vestibulo-ocular reflex (feedforward) system able to generate movements of the eyes to compensate for perturbations of the place and orientation of the head. However, on their own, these wired-in synergies are insufficient to account for the coordinations required for accurate gaze and focus control. The required coupling of conjugate, vergence, vertical and roll movements of the eyes with each other, and with focus control and with movements of the head differs from one shift of gaze to another depending on the initial and final gaze points. The wired-in synergies themselves have to be coordinated by a more comprehensive, overriding, non-linear, multivariable, adaptive, optimal, predictive, feedforward/feedback movement control system. Much of our previous work has been concerned with developing a systems theory description of how such a movement control can be achieved [83].

### 2.7. Singular Value Decomposition as a Model for Visual Feature Extraction

We have long emphasized the importance of orthogonalization (and deorthogonalization) in the central processing of sensory and motor signals [52,83,84,85]. Indeed, we have argued that orthogonalization is ubiquitous at all levels throughout all sensory and motor systems of the brain. Not only does it ensure that sensory information is encoded centrally in the most efficient way by removing redundancy, as argued by Barlow [86], but it is necessary for the nervous system to be able to form forward and inverse adaptive models of the non-linear dynamical relationships within and between sensory and motor signals. Previously we have described a network of neural adaptive filters able to extract independently-varying (orthogonal) feature signals using a non-linear, dynamical, Gramm–Schmidt orthogonalization algorithm [83]. The same process can be described mathematically by non-linear, dynamical, Q–R factorization or by non-linear singular value decomposition (SVD).

In Appendix A we give a theoretical description of a two-stage extraction within a hypercolumn of linear and non-linear stochastic orthogonal visual-image feature signals from corresponding left and right retinal hyperfields during an interval of fixed gaze using non-linear SVD. We propose that non-linear SVD provides a useful computational model for the extraction of image-point vectors by the slowly tuning synaptic connectivity of minicolumns within the hypercolumns of V1. As described in Appendix A, the resulting 30 (ball-park figure) orthogonal non-linear stochastic feature signals $\Sigma_{L}=\left(\Sigma_{L1},\cdots ,\Sigma_{L30}\right)$ extracted from the image $I_{L}$($\hat{\theta }$,$\;\hat{\varphi }$) on a left retinal hyperfield during an interval of a fixed gaze are represented by the temporospatial patterns of activity induced in 30 left ocular dominance minicolumns in a hypercolumn. Similarly, the 30 orthogonal non-linear stochastic feature signals $\Sigma_{R}=\left(\Sigma_{R1},\cdots ,\Sigma_{R30}\right)$ extracted from the image $I_{R}$($\hat{\theta }$,$\;\hat{\varphi }$) on the corresponding right retinal hyperfield during the same interval of fixed gaze are encoded by the temporospatial patterns of neural activity induced in 30 right ocular dominance minicolumns in the same hypercolumn.

The orthogonal feature signals are extracted from images on corresponding hyperfields across the left and right retinas and so, together, the hypercolumns provide an encoding of both central and peripheral visual fields. As stated by Rosenholtz [82], peripheral vision most likely supports a variety of visual tasks including peripheral recognition, visual search, and getting the “gist” of a scene. Incorporating the work of others [87,88,89], she models the encoding of peripheral vision with parameters that include luminance autocorrelations, correlations of magnitudes of oriented V1-like wavelets (Gabor filters) across different orientations, neighbouring positions and scales, and phase correlation across scale. She states that, when pooled over sparse local image regions that grow linearly with eccentricity, these provide a rich, high-dimensional, efficient, compressed encoding of retinal images. Given the change in size of hyperfields and in the density of rods and cones therein as a function of eccentricity, this description of the encoding of retinal images is consistent with the extraction by non-linear SVD of vectors of orthogonal image features $\Sigma_{L}$ and $\Sigma_{R}$ from hyperfields across the left and right retinas during each interval of fixed gaze.

For subsequent simplicity we refer to $\Sigma_{L}$ and $\Sigma_{R}$ as *image-point vectors*. As described in Appendix A, during an interval of fixed gaze they encode the 30-dimensional vectors of orthogonal non-linear stochastic features extracted from the images that project respectively on to left and right retinal hyperfields from small neighbourhoods of points on the surfaces of objects in the environment. The image-point vectors $\Sigma_{L}$ across all the left hyperfields form a 30-dimensional vector field **V**_L_ over all the left ocular dominance minicolumns in the hypercolumns of V1. Similarly, the image-point vectors $\Sigma_{R}$ from all the corresponding right hyperfields form a 30-dimensional vector field **V**_R_ over all the right ocular dominance minicolumns in the hypercolumns of V1. Due to the retinotopic projections between retinal hyperfields and cortical hypercolumns, the vector fields **V**_L_ and **V**_R_ can also be thought of as vector fields over the left and right retinal hyperfields, respectively.

Representing the extracted orthogonal visual feature vector fields **V**_L_ and **V**_R_ over hypercolumns and over retinal hyperfields in this way facilitates a mathematical framework appropriate for development of a Riemannian geometry theory of binocular vision. But this requires a mechanism for quantifying the depth of objects perceived. These depth measures then provide a coordinate system for the Riemannian manifold on which the above vector fields are defined.

### 2.8. Depth Perception

As can be quickly verified by closing one eye or by seeing depth in a flat two-dimensional (2D) picture, stereopsis is not the only mechanism in the brain for perception of depth. Indeed, a variety of mechanisms have evolved for depth perception. We refer to these as *top-down cognitive mechanisms* and they work in parallel to estimate the depth of points in the visual world [40,41,56,90]. Top-down cognitive processes employ information derived from occlusions, relative size, texture gradients, shading, height in the visual field, aerial perspective and perspective to estimate depth [91] and they depend on memorized experience [92]. Whenever an estimate of depth is altered by one or other top-down mechanism (e.g., as in the Ames room or the virtual expanding room described below) the geometry of the perceived visual space will change giving rise to an inhomogeneous geometry. This applies particularly to monocular and pictorial visual space but is not to deny that there exists an underlying visual space with a stable Riemannian geometry attributable to the size–distance relationship introduced by the eye.

Usually the depths estimated by the various depth-estimation modules are in agreement, but circumstances can arise where they disagree. Sometimes the contradictions are reconciled into a single coherent perception such as when seeing depth in a picture while at the same time seeing the flat plane of the picture. In other circumstances, one or more of the depth estimates is overruled leading to illusions and the analysis of illusions is important in perceptual science. Artists and magicians often take advantage of this phenomenon to trick the visual systems of their observers. When looking through the peep hole of a trapezoidal-shaped Ames room, for example, a normal room with parallel walls, horizontal floor and rectangular windows is seen rather than the actual distorted trapezoidal shape because experience tells us that rooms are normally shaped this way [93,94,95,96]. The Ames room illusion is compelling even to the extent of seeing people change size as they walk about in the room. Similarly, in the expanding virtual room experiment of Glennerster et al. [97], estimates of depth derived from convergence of the eyes, retinal disparity and optical flow as a person moves about in the virtual room are overruled in favour of a cognitive estimate based on the experience that rooms do not expand as we walk about within them.

Clearly there are many cues that can influence the perception of depth and, as argued by Gilinsky [3], changes in perceived depth can influence the perceived size of an object independently of the angle subtended by the object at the eye. Foley et al. [10] argued that while the retinal image decreases in size in proportion to object distance, the perceived size changes much less. The principal assumption in their model is that, in the computation of perceived extent, the visual angle undergoes a magnifying transformation. Given the variety of cues and cognitive mechanisms that can contribute to the perception of depth, it should not be surprising that experimental conditions have a strong influence on experimental results, particularly on the experimentally measured geometry of 3D perceived visual space.

While not underestimating the importance of top-down cognitive mechanisms, we focus in this paper on binocular depth perception as the only means of obtaining an *absolute* measure of Euclidean depth. For the vector fields of left and right retinal images encoded during an interval of fixed gaze to have meaning in terms of events in the outside world, the vectors of corresponding left and right hyperfield image features encoded within each hypercolumn during an interval of fixed gaze have to be associated with coordinates of the points $a_{Li}$ and $a_{Ri}$ in the outside world projecting on to corresponding left and right hyperfields respectively. In Section 2.9 and Section 2.10 we outline processes able to compute those coordinates.

### 2.9. Cyclopean Gaze Coordinates

**Figure 1:**
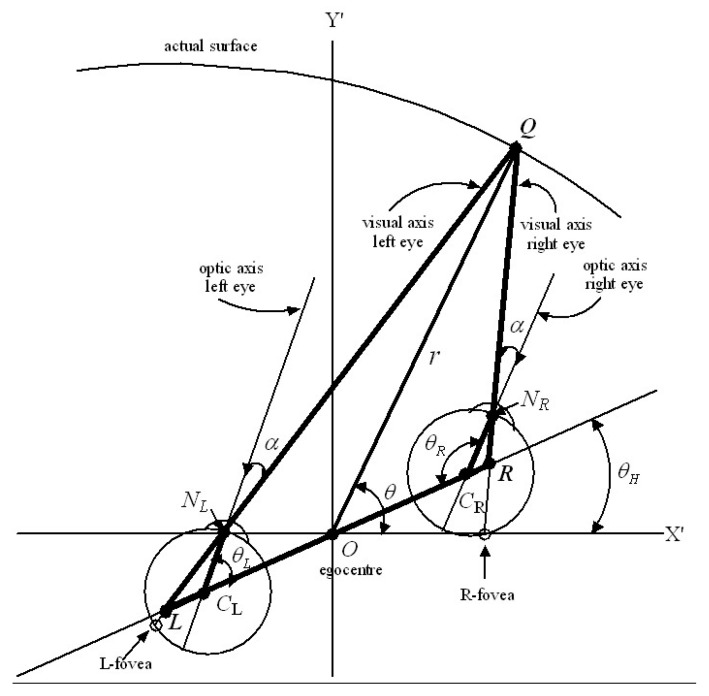
A schematic 2D diagram based on the reduced model of the human eye [42]. *C_L_* and *C_R_* are the centres of rotation of the left and right eye, respectively, and *N_L_* and *N_R_* are the nodal points. The optic axis for each eye connects the centre of rotation to the nodal point. The visual axis for each eye connects the fovea to the nodal point. The distance between *C_L_* and *C_R_* is known as *d*. Its midpoint *O* is known as the egocentre and marks the position of a hypothetical cyclopean eye. The distances *C_L_N_L_* and *C_R_N_R_* are the same for each eye and are known as *r_E_*. The angle α between the optic axis and the visual axis is also the same for each eye and is typically about 5 degrees in adults. $\theta_{H}$ gives the angle of the head relative to a translated external reference frame ($X^{\prime }$, $Y^{\prime }$ ) and $\theta_{L}$ and $\theta_{R}$ give the angles of the left and right eyes relative to the head when gaze is fixed on a surface point *Q* in the environment. The diagram shows the cyclopean gaze vector *OQ* in relation to the above geometry.

The nervous system has only one bottom-up mechanism for obtaining an absolute measure of the Euclidean distance to gaze points in the environment [91,98]. This module uses afferent information encoding the place and orientation of the head in the environment as well as proprioceptive and vestibular information encoding the angles of the eyes within the head [98]. By minimizing disparity between images on the left and right foveas, the gaze control system adjusts the orientation of the head in the environment as well as the angles of the eyes in the head so that the visual axes of the eyes intersect accurately at the gaze point. The geometry is shown in Figure 1, and is based on the *reduced model of the human eye* [42] which takes into account the fact that the visual axis does not pass through the centre of rotation of the eye.

The position of the egocentre *O* measured with respect to the external reference frame (X,Y,Z) provides a measure of the egocentric place of the head in the environment. Since *O* is the point where a cyclopean eye would be located (if we had one) we define the line *OQ* connecting the *O* to the point of fixed gaze *Q* to be the *cyclopean gaze vector*. The distances *d* and *r_E_* and the angle α are anatomical parameters that change with growth of the head and eye. Since these parameters influence the geometrical optics of images projected on to the retinas it does not seem unreasonable to suggest that the nervous system is able to model them adaptively through experience, for example, by modelling the relationship between the depth of an object and the size of its image on the retina, and by sensing the change in place of the head required to match the image on one retina with the memorized image on the other. The place and orientation of the head in the environment are encoded by neural activity in the hippocampus and parahippocampus so, referring to Figure 1, the angle $\theta_{H}$ of the head relative to the translated external coordinates ($X^{\prime }$,$Y^{\prime }$) is known, and the angles of rotation $\theta_{L}$ and $\theta_{R}$ of the left and right eye within the head are sensed proprioceptively. Using the geometry of Figure 1, it can be shown that these known variables $\theta_{H}$, $\theta_{L}$, $\theta_{R}$, *d*, *r_E_* and α completely determine the Euclidean distance and angle from each eye to the gaze point as well as the length and direction (r, θ) of the cyclopean gaze vector *OQ*. This can be demonstrated by basic trigonometry (sine rule and cosine rule) of the three triangles *N_L_LC_L_*, *N_R_LC_R_*, and *LQR*. Importantly, this is not to say that the nervous system ‘does’ trigonometry in the same way we do. It is simply to establish that the information available to it is sufficient to determine uniquely the length and direction of the cyclopean gaze vector.

Thus we propose that during each interval of fixed gaze the nervous system is able to compute the *cyclopean gaze coordinates* (r, θ, φ) of the fixed gaze point *Q* and hold them in working memory within the hypercolumn(s) receiving retinotopic input from the left and right foveal hyperfields. The length r of the cyclopean gaze vector *OQ* provides an egocentric measure of the Euclidean distance (depth) of the gaze point *Q* while the angles (θ, φ) provide a measure of the cyclopean direction of gaze relative to the external reference frame (X,Y,Z). A third angle ψ equal to a rotation about the cyclopean gaze vector *OQ* is needed to completely specify the cyclopean gaze coordinates. Rolling of the head in the environment as well as internal and external rotation of the eyes in the head introduce a roll angle ψ about the cyclopean gaze vector (r, θ, φ). However, while this roll angle ψ is important in that it leads to rotation of the perceived visual image (e.g., when lying down or standing on one’s head), we will ignore it temporarily. We show in Section 5.2 that a roll ψ about the gaze vector (r, θ, φ) transforms the positions of all retinal image points in the 3D perceived visual space in an isometric fashion without changing the size or shape of the local image.

### 2.10. Cyclopean Coordinates of Peripheral Image Points

**Figure 2:**
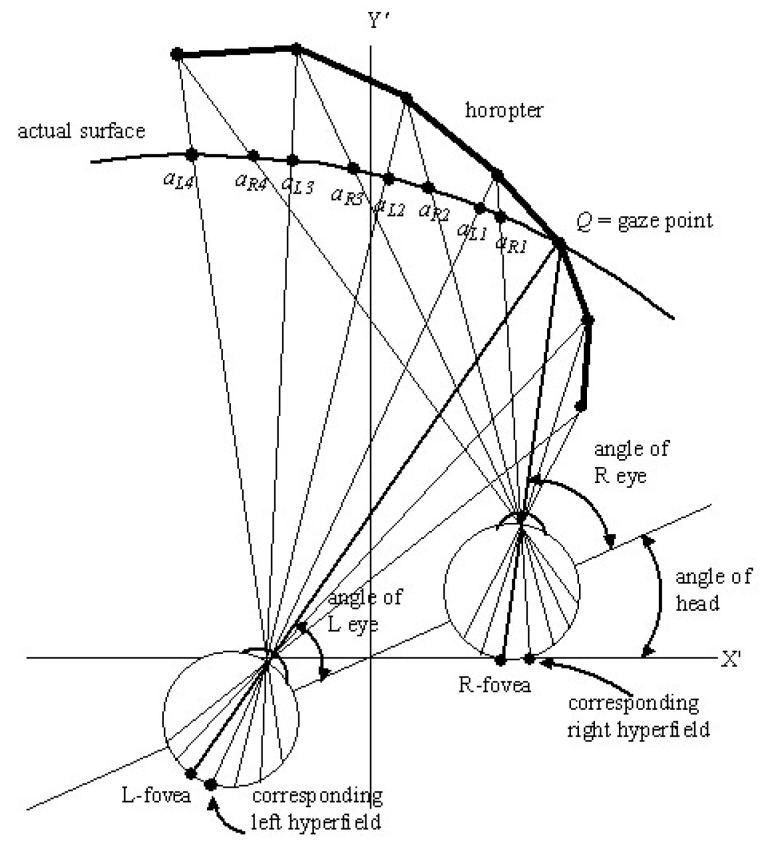
A schematic 2D diagram illustrating the angle of the head relative to a translated external reference frame ($X^{\prime }$, $Y^{\prime }$ ) and the angles of the left and right eyes relative to the head when gaze is fixed on a surface point *Q* in the environment. The left and right eye visual axes are straight lines connecting the fovea through the nodal point of the eye to the gaze point *Q*. The fan-shaped grids of straight lines passing through the nodal point of each eye connect corresponding left and right retinal hyperfields to points $a_{Li}$ and $a_{Ri}$, respectively, on the surface. The image point $a_{Li}$ projecting to a left retinal hyperfield is translated by a small amount relative to the image point $a_{Ri}$ projecting to the corresponding right retinal hyperfield. Thus the points $a_{Li}$ and $a_{Ri}$ induce a disparity between the images projected to the corresponding left and right retinal hyperfields. The diagram also includes the hypothetical surface known as an horopter. This contains the points which induce no disparity between the images projected to corresponding left and right hyperfields.

When gaze is fixed, points *q* other than the fixed gaze point *Q* on the surface of an object project with different angles into the two eyes and impinge on different retinal coordinates in the left and right eyes. This creates disparity between the left and right peripheral retinal images. Put alternatively, with the gaze point Q=(r, θ, φ) fixed, corresponding hyperfields on the left and right retinas receive projections from different points $a_{Li}$ and $a_{Ri}$ (i=1,2,… ) on the surface of an object in the environment. This is illustrated in Figure 2.

We now propose that the difference $\Sigma_{L}-\Sigma_{R}$ between image-point vectors within each hypercolumn are computed and held on-line in binocular minicolumns within the same hypercolumns. In other words, we suggest that the high-dimensional statistics extracted in the form of image-point vectors from hyperfields across the retinas provide a rich encoding of disparity between images on the left and right retinas. Since certain ganglion cells respond to motion in retinal images, this can include disparity of velocity fields as proposed by Cormack et al. [99]. These difference vectors encode the disparity between the images from the points $a_{Li}$ and $a_{Ri}$ (i=1,2,… ) on corresponding left and right hyperfields. The image-disparity vectors $\Sigma_{L}-\Sigma_{R}$ form a disparity vector field over the hypercolumns. It has been established [100,101] that the geometry of visual disparity fields can be expressed in terms of four differential components; viz., expansion or dilation, curl or rotation, and two components of deformation or shear. An algebraic combination of these operators allows one eye image to be mapped on to the other. Likewise, in the Riemannian geometry theory presented here, we see the gradients, translations, rotations, dilations, and shear of the components of the image-disparity vector field $\Sigma_{L}-\Sigma_{R}$ over the hypercolumns as providing detailed measures of the local disparity between images on corresponding left and right retinal hyperfields.

The image-point vectors $\Sigma_{L}$ and $\Sigma_{R}$ provide sufficient information to accurately reconstruct the left and right hyperfield images. Hence the vectors $\Sigma_{L}-\Sigma_{R}$ between corresponding and neighbouring hyperfields in left and right eyes provide sufficient information to accurately reconstruct the difference between the images giving a measure of the shifts $a_{Ri}-a_{Li}$, $a_{R\left(i+1\right)}-a_{Li}$, $a_{R\left(i+1\right)}-a_{Ri}$ and $a_{L\left(i+1\right)}-a_{Li}$ (i=1,2,… ) between them. With ‘knowledge’ of these shifts, the grid of overlapping triangles formed by the straight lines emanating from corresponding left and right retinal hyperfields via the nodal point of each eye (Figure 2) allows the cyclopean coordinates $\left(r_{a_{Li}},\;\theta_{a_{Li}},\;\varphi_{a_{Li}}\right)$ and $\left(r_{a_{Ri}},\;\theta_{a_{Ri}},\;\varphi_{a_{Ri}}\right)$ for a line drawn from the egocentre *O* to each of the points $a_{Li}$ and $a_{Ri}$ (i=1,2,… ) to be computed relative to the cyclopean gaze coordinates (r, θ, φ) of the fixed gaze point *Q*.

We propose that, along with the image-point vectors of all the points $a_{Li}$ and $a_{Ri}$, the cyclopean coordinates $\left(r_{a_{Li}},\;\theta_{a_{Li}},\;\varphi_{a_{Li}}\right)$ and $\left(r_{a_{Ri}},\;\theta_{a_{Ri}},\;\varphi_{a_{Ri}}\right)$ (i=1,2,… ) are also held on line in working memory by minicolumns within the hypercolumns receiving retinotopic projections from the corresponding left and right retinal hyperfields. In other words, during each interval of fixed gaze each hypercolumn encodes the two 30-dimensional vectors $\Sigma_{L}$ and $\Sigma_{R}$ of orthogonal image features extracted from the images on corresponding left and right retinal hyperfields and the cyclopean coordinates $\left(r_{a_{Li}},\;\theta_{a_{Li}},\;\varphi_{a_{Li}}\right)$ and $\left(r_{a_{Ri}},\;\theta_{a_{Ri}},\;\varphi_{a_{Ri}}\right)$ of the points $a_{Li}$ and $a_{Ri}$ (i=1,2,… ) projecting the images on to those corresponding left and right hyperfields. To emphasize the fact that the coordinates $\left(r_{a_{Li}},\;\theta_{a_{Li}},\;\varphi_{a_{Li}}\right)$ and $\left(r_{a_{Ri}},\;\theta_{a_{Ri}},\;\varphi_{a_{Ri}}\right)$ (i=1,2,… ) are different even though they are encoded within the same hypercolumn, we use the term *cyclopean gaze coordinates* for the fixed gaze point *Q* and the term *cyclopean coordinates* for all other points $a_{Li}$ and $a_{Ri}$ in the peripheral visual field.

Figure 2 also shows the *horopter,* defined in the Oxford Dictionary to be “a line or surface containing all those points in space of which images fall on corresponding points of the retinae; the aggregate of points of which are seen single in any given position of the eyes*”* [102]. As can be seen in the figure, this hypothetical curved line/surface contains the gaze point *Q,* and is constructed by plotting the intersections of all the straight lines that come from corresponding left and right hyperfields and pass through the nodal point of each eye. Because every point on the horopter projects to corresponding left and right retinal hyperfields, an actual surface that mimics it would generate a zero disparity image. From Figure 2 it can be seen that the shape of the horopter depends on the cyclopean gaze coordinates Q=(r, θ, φ) that in turn depend on the angles of the eyes in the head and the angle of the head relative to an external reference frame. It becomes less curved as the Euclidean distance r to the fixed gaze point increases. With the gaze point *Q* and the orientation of the head fixed, the disparity between points on any *actual surface* in the environment projecting on to left and right retinal hyperfields increases in a non-linear way as the distance between the actual surface and the horopter increases. Because of this, the disparity between points on any actual surface varies in a non-linear fashion across the retinas depending on how its distance and shape varies from that of the horopter.

Despite this complicated variation of the image-disparity field across the retinas, its non-linear dependence on the location of the gaze point *Q*, the orientation of the head in the environment, and on the location of actual surfaces in the outside world relative to the horopter, it is well known from random-dot stereogram experiments [90,103,104] that the visual system can extract depth and direction of points on the surfaces of objects in the peripheral visual fields. Random-dot stereograms provide compelling evidence that disparity between left and right retinal images plays an important role in peripheral depth perception. Marr [90] defined *disparity* to mean the angular discrepancy in the positions of the images of an object in the environment in the two eyes. Marr and Poggio [105] developed a computer algorithm to detect disparities in computer-generated random-dot stereograms. Our method for measuring disparity based on differences between image-point vectors extracted from corresponding retinal hyperfields is slightly different and less demanding on neural resources in that it obviates the need for the stereo correspondence neural network proposed by Marr and Poggio or for local pattern matching (local correlation) mechanisms. Differencing 30 or so left and right hyperfield image features encoded in ocular dominance columns within a hypercolumn pools activity from an extended region of V1 and involves multiple image features. This is not inconsistent with the detailed picture of neural architectures for stereo vision in V1 described by Parker et al. [106].

## 3. The Three-Dimensional Perceived Visual Space

The size–distance relationship introduced into retinal images by the optics of the eye is independent of the scene being viewed and of the position of the head in the environment. While the retinal image itself changes from one viewpoint to another, the geometry of the 3D perceived visual space derived from stereoscopic vision with estimates of Euclidean depth based on triangulation remains the same regardless of the scene and of the place of the head in the environment. In this section we present theory that describes a means for the visual system to form an internal representation of the 3D perceived visual space and of the visual images in that space viewed from a fixed place.

### 3.1. Gaze-Based Visuospatial Memory

When the head is in a fixed place and gaze is shifted from one gaze point to another the vector fields **V**_L_ and **V**_R_ of image-point vectors $\Sigma_{L}\left(r_{a_{Li}},\;\theta_{a_{Li}},\;\varphi_{a_{Li}}\right)$ and $\Sigma_{R}\left(r_{a_{Ri}},\;\theta_{a_{Ri}},\;\varphi_{a_{Ri}}\right)$ over the hypercolumns described in Section 2.10 are replaced by new image-point vectors and by new vector fields associated with the next gaze point in the scanning sequence. To build a visuospatial memory of an environment through scanning we argue that the information encoded by the vector fields **V**_L_ and **V**_R_ during a current interval of fixed gaze must be stored before the gaze is shifted and the information lost. Such memory is accumulated over time and scanning of an environment from a fixed place does not have to occur in one continuous sequence. Images associated with different gaze points from a fixed place can be acquired (and if necessary overwritten) in a piecemeal fashion every time the person passes through that given place.

We propose that, at the end of each interval of fixed gaze, the 30-dimensional image-point vectors $\Sigma_{Li}$ encoding left-hyperfield images within each hypercolumn are stored into a *gaze-based association memory* or G*-memory* in association with their cyclopean coordinates $\left(r_{a_{Li}},\;\theta_{a_{Li}},\;\varphi_{a_{Li}}\right)$. Similarly, the 30-dimensional image-point vectors $\Sigma_{Ri}$ encoding right-hyperfield images within each hypercolumn are stored in the same G*-*memory in association with their cyclopean coordinates $\left(r_{a_{Ri}},\;\theta_{a_{Ri}},\;\varphi_{a_{Ri}}\right)$. In other words, the cyclopean coordinates $\left(r_{a},\;\theta_{a},\;\varphi_{a}\right)$ for each point ‘a’ in the 3D Euclidean outside space provides an *accession code* for the G*-*memory. This concept of an accession code for memory stems from an earlier proposal of ours (see [83] Section 8.3) and is analogous to the way the accession code in a library catalogue points to a book in the library. A particular cyclopean coordinate $\left(r_{a},\;\theta_{a},\;\varphi_{a}\right)$ gives the ‘site’ in the G-memory where two 30-dimensional image-point vectors $\Sigma_{L}\left(r_{a},\;\theta_{a},\;\varphi_{a}\right)$ and $\Sigma_{R}\left(r_{a},\;\theta_{a},\;\varphi_{a}\right)$ are stored. Actually, such a ‘site’ in an association memory network is distributed across the synapses of a large number of neurons in the network and the information is retrieved by activating the network with an associated pattern of neural activity encoding the cyclopean coordinate $\left(r_{a},\;\theta_{a},\;\varphi_{a}\right)$, as in a Kohonen association neural network (see [83] for further description).

The ‘library accession code’ analogy provides a simplified metaphor for the storage and retrieval of information in an association memory network and is used throughout the rest of the paper. Each site in the G-memory corresponds to a cyclopean image point $\left(r_{a},\;\theta_{a},\;\varphi_{a}\right)$ in the Euclidean environment. As shown in Appendix A the left and right hyperfield image-point vectors $\Sigma_{L}\left(r_{a},\;\theta_{a},\;\varphi_{a}\right)$ and $\Sigma_{R}\left(r_{a},\;\theta_{a},\;\varphi_{a}\right)$ extrapolated from the same image point $\left(r_{a},\;\theta_{a},\;\varphi_{a}\right)$ in the outside space require 30 components (features) to adequately encode the non-linear stochastic characteristics of images on the hyperfields during each interval of fixed gaze. Thus each memory site can be thought of geometrically as a 30-dimensional vector space able to store two 30-dimensional image-point vectors. Because of disparity between left and right retinal images, the two 30-dimensional image-point vectors $\Sigma_{L}\left(r_{a},\;\theta_{a},\;\varphi_{a}\right)$ and $\Sigma_{R}\left(r_{a},\;\theta_{a},\;\varphi_{a}\right)$ stored at the image-point site $\left(r_{a},\;\theta_{a},\;\varphi_{a}\right)\;$ in the G-memory derive from different locations on the left and right retinas and are encoded within different hypercolumns. Thus, the storage of individual left and right image-point vectors $\Sigma_{L}\left(r_{a_{Li}},\;\theta_{a_{Li}},\;\varphi_{a_{Li}}\right)$ and $\Sigma_{R}\left(r_{a_{Ri}},\;\theta_{a_{Ri}},\;\varphi_{a_{Ri}}\right)$ into the G-memory in association with their respective cyclopean coordinates $\left(r_{a_{Li}},\;\theta_{a_{Li}},\;\varphi_{a_{Li}}\right)$ and $\left(r_{a_{Ri}},\;\theta_{a_{Ri}},\;\varphi_{a_{Ri}}\right)$ performs the task of linking disparate sites on left and right retinas receiving an image from the same image point in the environment.

For a fixed gaze point Q=(r, θ, φ), the left and right image-point vectors associated with *foveal* hyperfields are fused by the gaze control system into a single hyperfield image-point vector. However, as the point $q=\left(r_{a},\;\theta_{a},\;\varphi_{a}\right)$ in the environment moves away from the fixed gaze point Q=(r, θ, φ), the difference between the left and right image-point vectors $\Sigma_{L}\left(r_{a},\;\theta_{a},\;\varphi_{a}\right)$ and $\Sigma_{R}\left(r_{a},\;\theta_{a},\;\varphi_{a}\right)$ increases because the size of retinal hyperfields and the densities of rods and cones change with retinal eccentricity. The superimposed left and right hyperfield images stored at the same site $\left(r_{a},\;\theta_{a},\;\varphi_{a}\right)$ in the G-memory become less well fused and, consequently, appear more fuzzy.

We define the *functional region of central vision* to be an area containing all those points $\left(r_{a},\;\theta_{a},\;\varphi_{a}\right)$ in the Euclidean environment where the fuzziness and imprecision of location of the superimposed left and right hyperfield images $\Sigma_{L}\left(r_{a},\;\theta_{a},\;\varphi_{a}\right)$ and $\Sigma_{R}\left(r_{a},\;\theta_{a},\;\varphi_{a}\right)$ stored at the same site $q=\left(r_{a},\;\theta_{a},\;\varphi_{a}\right)\;$ in G-memory is acceptable for the visual task at hand. The size of this region varies with the visual resolution required for the particular task and with the Euclidean depth of gaze r. It varies with the extent of cluttering in the peripheral visual field [82]. Points $q=\left(r_{a},\;\theta_{a},\;\varphi_{a}\right)$ in peripheral visual fields where the image-point vectors $\Sigma_{L}\left(r_{a},\;\theta_{a},\;\varphi_{a}\right)$ and $\Sigma_{R}\left(r_{a},\;\theta_{a},\;\varphi_{a}\right)$ stored at the same site $\left(r_{a},\;\theta_{a},\;\varphi_{a}\right)$ in G-memory are so different they cannot be fused into a single vector, give rise to the perception of double images, one from the left eye and one from the right eye, that may appear fuzzy.

As gaze is shifted from one point in the environment to another, large regions of the peripheral visual fields for the various gaze points overlap. Consequently, the sites $q=\left(r_{a},\;\theta_{a},\;\varphi_{a}\right)$ in G-memory where the peripheral image-point vectors associated with a current point of fixed gaze are to be stored may overlap with sites where image-point vectors associated with previous gaze points are already stored. We propose the following rule for determining whether or not image-point vectors already stored in G-memory are overwritten by the image-point vectors associated with the current gaze point: *Image-point vectors at sites in the peripheral visual field are only overwritten if the absolute difference* $\left|\Sigma_{L}\left(r_{a},\;\theta_{a},\;\varphi_{a}\right)-\Sigma_{R}\left(r_{a},\;\theta_{a},\;\varphi_{a}\right)\right|$ *between the left and right image-point vectors already stored at the site* $\left(r_{a},\;\theta_{a},\;\varphi_{a}\right)$ is larger than the absolute difference between the two image-point vectors for the same site $\left(r_{a},\;\theta_{a},\;\varphi_{a}\right)$ *associated with the current gaze point.* Using this rule, the images accumulated in G-memory from any given scanning pattern will consist of those image points that are closest to their regions of functional vision. As the number of gaze points in the scanning pattern increases, the above rule causes the acuity of the accumulated peripheral image to improve. In the extreme case, with an infinite number of gaze points in the scanning pattern, gaze is shifted to every point in the environment and only fused foveal images are stored at every site in an infinite G-memory.

This is consistent with the evidence reviewed by Hulleman and Olivers [107] showing that it is the fixation of gaze that is the fundamental unit underlying the way the cognitive system scans the visual environment for relevant information. The finer the detail required in the search task, the smaller the functional region of central vision and the greater the number of fixations required. The notion of a variable functional region of central vision provides a link between visual attention and peripheral vision and attributes a more important role to peripheral vision as argued by Rosenholtz [82].

### 3.2. A Riemannian Metric for the G-Memory

As indicated in Section 1, the size of the 2D image on the retina varies in inverse proportion to the Euclidean distance between the nodal point of the eye and the object. A key proposal of the present theory is that the nervous system can model adaptively the relationship between the Euclidean distance to an object in the outside world (sensed by triangulation) and the size of its image on the retina. The notion that this modelling is adaptive is consistent with the observation that the visual system can adapt to growth of the eye and to wearing multifocal glasses. We hypothesize that the modelled size–distance relationship can be applied to three dimensions and encoded in the form of a Riemannian metric g(r, θ, φ) stored at every site q=(r, θ, φ) in the 3D cyclopean G-memory (for simplicity here and in what follows we have dropped the subscript a for peripheral points). In other words, we hypothesize that *the perceived size of a 3D object in the outside world varies in inverse proportion to the cyclopean Euclidean distance* r *between the egocentre and the object*, this being simply a reflection of the 2D size–distance relationship introduced by the optics of the eye.

When the G-memory is endowed with the metric g(r, θ, φ) it can be represented geometrically as a Riemannian manifold (G*,*g). At each point q in the (G*,*g) manifold there exists a 3D tangent space $T_{q}G$ spanned by *coordinate basis vectors* ∂r=∂∂r, ∂θ=∂∂θ, ∂φ=∂∂φ. Any tangential velocity vector v in the tangent space $T_{q}G$ can be expressed as a linear combination of the coordinate basis vectors $\left(\partial_{r},\partial_{\theta },\partial_{\varphi }\right)$ spanning that space. The metric inner product of any two vectors v and w in the tangent vector space $T_{q}G$ at the point q is denoted by ${\langle v,w\rangle }_{g}$ and the angle σ between the two vectors is computed using cosσ=〈v,w〉g(‖v‖g·‖w‖g). When the angle σ between the two vectors is π2 rad the two vectors are said to be g-orthogonal. We use this terminology throughout the paper.

The metric distance between any two points $q_{i}$ and $q_{j}$ in the (G*,*g) manifold is obtained by integrating the metric speed (i.e., metric norm or g-norm ‖v‖g=〈v,v〉g12) of the tangential velocity vector v along the geodesic path connecting the two points. This provides the visual system with a type of *measure* that can be used to compute distances, lengths and sizes in the perceived visual manifold (G*,*g). Since the metric g changes from point to point in the manifold, and the metric speed ${\left\|v\right\|}_{g}$ depends on the metric, it follows that the metric distance between any two points $q_{i}$ and $q_{j}$ depends on where those points are located in the (G*,*g) manifold. In other words, the metric g stretches or compresses (warps) the perceived visual manifold relative to the 3D Euclidean outside space.

In discussing the perception of objects, Frisby and Stone [40] suggest that it would be helpful to dispense with the confusing term *size constancy* and instead concentrate on the issue of the nature and function of the size representations that are built by our visual system. In keeping with this, we propose that, consistent with the size–distance relationship introduced by the optics of the eye, *the Riemannian metric* g(r, θ, φ) *on the perceived visual manifold varies inversely with the square* $r^{2}$ *of the cyclopean Euclidean distance* r *and is independent of the cyclopean direction* (θ, φ). This causes metric distances between neighbouring points in the perceived visual manifold (G*,*g) to vary in all three dimensions in inverse proportion to cyclopean Euclidean distance r in the outside world. As a result, the perceived size of 3D objects in the outside world varies in inverse proportion to the cyclopean Euclidean distance r without changing their perceived infinitesimal shape. We use the term “infinitesimal shape” because, in attempting to define and establish a measure of subjective distance, Gilinsky states that “the depth dimension becomes perceptively compressed at greater distances” [3] (p. 463). Consequently, as a macroscopic object recedes its shape appears to change because its contraction is greater in the depth extent than in height or width.

The proposal that the Riemannian metric g(*r*) varies only as a function of cyclopean Euclidean distance r and is independent of cyclopean direction (θ, φ) implies that the metric is constant on concentric spheres in the outside world centred on the egocentre. These concentric spheres play an important role in describing the geometry of the perceived visual manifold (G,g) and from now on we refer to them simply as the *visual spheres*.

For the size of an object to be perceived as changing in all three dimensions in inverse proportion to the cyclopean Euclidean distance r without changing its infinitesimal shape, the Riemannian metric g(*r*) must be such that the g-norm ${\left\|v\right\|}_{g}$ of the tangential velocity vector at each point in the perceived visual manifold (G,g) is equal to the norm ${\left\|v\right\|}_{\bar{g}}$ of the velocity vector in Euclidean space (where $\bar{g}$ is the metric of the 3D Euclidean space) divided by the cyclopean Euclidean distance r, that is, ${\left\|v\right\|}_{g}=\left(1⁄r\right){\left\|v\right\|}_{\bar{g}}$ at each point.

If egocentric Cartesian coordinates (x,y,z) are employed, the Euclidean metric for the outside world is:

(1) $$\bar{g}\left(x,y,z\right)=\left[\begin{array}{ccc} 1 & 0 & 0\\ 0 & 1 & 0\\ 0 & 0 & 1 \end{array}\right],$$

and the required Riemannian metric g(x,y,z) for the perceived visual manifold (G,g) is:

(2) ‖v‖g=〈gv,v〉12=(1r)‖v‖g¯=〈(1r2)g¯v,v〉12.

That is,

(3) g((x2+y2+z2)12)=1x2+y2+z2g¯= [1x2+y2+z20001x2+y2+z20001x2+y2+z2].

However, the depth and direction of gaze is best described using spherical coordinates (r, θ, φ). Thus we require the Riemannian metric of the perceived visual manifold to be expressed in terms of (r, θ, φ). The transformation between spherical coordinates (r, θ, φ) and Cartesian coordinates (x, y, z) in Euclidean space is given by:

(4) $$\begin{array}{l} x=r\cos\varphi \cos\theta ,\\ y=r\cos\varphi \sin\theta ,\\ z=r\sin\varphi . \end{array}$$

When the Euclidean metric in Equation (1) is pulled back to spherical coordinates using Equation (4) we get:

(5) $$\bar{g}\left(r,\;\theta ,\;\varphi \right)=\left[\begin{array}{ccc} 1 & 0 & 0\\ 0 & r^{2}\cos^{2}\varphi & 0\\ 0 & 0 & r^{2} \end{array}\right].$$

Consequently, the Riemannian metric g(r, θ, φ) for the perceived visual manifold is:

(6) ‖v‖g=〈gv,v〉12=〈(1r2)g¯v,v〉12.

That is,

(7) g(r, θ, φ)=1r2g¯(r, θ, φ)=[1r2000cos2φ0001],

where:

(8) $$v=\left[\begin{array}{c} \dot{r}\\ \dot{\theta }\\ \dot{\varphi } \end{array}\right].$$

In Euclidean space, using spherical coordinates, tangential velocities are related to angular velocities by:

(9) $$\left[\begin{array}{c} v^{r}\\ v^{\theta }\\ v^{\varphi } \end{array}\right]=\left[\begin{array}{c} \dot{r}\\ r\left(\cos\varphi \right)\dot{\theta }\\ r\dot{\varphi } \end{array}\right].$$

If the inverses of the relationships in Equation (9) are used to express the angular velocities ($\dot{r},\;\dot{\theta },\;\dot{\varphi })$ in terms of the tangential velocities ($v^{r},v^{\theta },\;v^{\varphi })$, then the terms $r^{2}cos^{2}\varphi$ and $r^{2}$ in Equation (5) cancel and we obtain:

(10) 〈[1000r2cos2φ000r2][vrvθrcosφvφr],[vrvθrcosφvφr]〉12=〈[100010001][vrvθvφ],[vrvθvφ]〉12.

In other words, to compute the norm of the Euclidean tangential velocity vector ${\left[\begin{array}{ccc} v^{r} & v^{\theta } & v^{\varphi } \end{array}\right]}^{T}$ in Euclidean spherical coordinates we require the Euclidean metric:

(11) $$\bar{g}=\left[\begin{array}{ccc} 1 & 0 & 0\\ 0 & 1 & 0\\ 0 & 0 & 1 \end{array}\right].$$

Thus the Riemannian metric matrix required to compute the g-norm $v_{g}$ of the tangential velocity vector ${\left[\begin{array}{ccc} v^{r} & v^{\theta } & v^{\varphi } \end{array}\right]}^{T}$ at any point (r, θ, φ) in the perceived visual manifold (G,g) is:

(12) g(r)=(1r2)g¯=[1r20001r20001r2].

## 4. Quantifying the Geometry of the Perceived Visual Manifold

Given the metric g in Equation (7) and/or Equation (12) depending on the coordinates employed, the theorems of Riemannian geometry can be applied to compute measures of the warping of the perceived visual manifold (G,g). In this section we use the geometry to quantify: (i) the relation between perceived depth and Euclidean distance in the outside world; (ii) the illusory accelerations associated with an object moving at constant speed in a straight line in the outside world; (iii) the perceived curvatures and accelerations of lines in the outside world; (iv) the curved accelerating trajectories (geodesic trajectories) in the outside world perceived as constant speed straight lines; (v) the Christoffel symbols describing the change of coordinate basis vectors from point to point in the perceived visual manifold; and (vi) the curvature at every point in the perceived visual manifold. Together, these Riemannian measures provide a detailed quantitative description of the warped geometry of the perceived visual manifold that can be compared with measures obtained experimentally.

### 4.1. The Relationship Between Perceived Depth and Euclidean Distance

As described by Lee [57] (Chapter 3), two metrics $g_{1}$ and $g_{2}$ on a Riemannian manifold are said to be *conformal* if there is a positive, real-valued, smooth function f on the manifold such that $g_{2}=fg_{1}$. Two Riemannian manifolds $\left(M_{1},g_{1}\right)$ and $\left(M_{2},g_{2}\right)$ are said to be *conformally equivalent* if there is a diffeomorphism (i.e., one-to-one, onto, smooth, invertible map) $\Phi :M_{2}\to M_{1}$ between them such that the pull back $\Phi^{*}g_{1}$ is conformal to $g_{2}$. Conformally equivalent manifolds have the same angles between tangent vectors at each point but the g-norms of the vectors are different and the lengths of curves and distances between points are different. Conformal mappings between conformally equivalent manifolds preserve both the angles and the infinitesimal shape of objects but not their size or curvature. For example, a conformal transformation of a Euclidean plane with Cartesian rectangular coordinates intersecting at right angles produces a compact plane with curvilinear coordinates that nevertheless still intersect at right angles [108].

Let us now consider two spaces, one being $\left(\bar{G},\bar{g}\right)$ corresponding to the Euclidean outside world with Euclidean metric $\bar{g}$ (Equation (5)) and Euclidean distance $\bar{r}$ and the other being (G,g) corresponding to the perceived visual manifold with metric g (Equations (7) or (12)) and metric distance r. The Riemannian manifold (G,g) is, by definition, locally Euclidean and can be mapped diffeomorphically to the Euclidean space $\left(\bar{G},\bar{g}\right)$. The symbol r can be used, therefore, to represent both the distances from the origin in $\left(\bar{G},\bar{g}\right)$ and from the egocentre in (G,g). We use this convention throughout the paper, however in this section (and only this section) we separate $\bar{r}$ and r because we are interested in the relationship between them. Comparing Equation (5) and Equation (7), it can be deduced that g=(1r¯2)g¯. This shows that the 3D Euclidean outside world $\left(\bar{G},\bar{g}\right)$ and the 3D perceived visual manifold (G,g) are conformally equivalent with conformal metrics $\bar{g}$ and g and with 1r¯2 equalling the positive, smooth, real-valued function fmentioned above.

Thus, from the theory of conformal geometry the following properties of the perceived visual manifold follow: (a) Objects appear to change in size in inverse proportion to the Euclidean distance $\bar{r}$ in the Euclidean outside world without changing their apparent infinitesimal shape. (b) The perceived visual manifold (G,g) is isotropic at the egocentre; i.e., the apparent change in size with Euclidean distance $\bar{r}$ is the same in all directions radiating out from the egocentric origin. (c) There exists a diffeomorphic map between the two manifolds but it does not preserve the metric; i.e., the map is not an isometry. While there is a one-to-one mapping between points in the outside space and points in the perceived visual manifold, the two spaces are not isometric so distances between points are not preserved. (d) Angles between vectors in corresponding tangent spaces $T_{\left(\bar{r},\bar{\theta },\bar{\varphi }\right)}\bar{G}$ and $T_{\left(r,\theta ,\varphi \right)}G$ are preserved. (e) As the Euclidean distance $\bar{r}$ increases towards infinity in $\left(\bar{G},\bar{g}\right)$ the perceived distance r in (G,g) converges uniformly to a limit point. Stars in the night sky, for example, appear as dots of light in the dome of the sky. (f) If $\bar{r}$ is the Euclidean distance from *O* to a point in $\left(\bar{G},\bar{g}\right)$, the perceived distance r from *O* to the corresponding point in (G,g) can be computed using Equation (2) by integrating the metric norm of the unit radial velocity vector $\partial_{\bar{r}}$ along the radial path to obtain:

(13) r=∫1r¯〈(1r¯2)∂r¯,∂r¯〉g¯12dr¯=∫1r¯1r¯dr¯.

**Figure 3:**
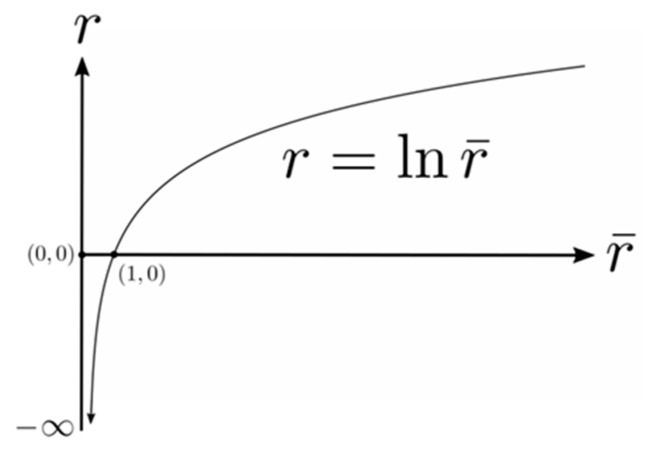
A graph showing the logarithmic relationship between the perceived distance r and the actual Euclidean distance $\bar{r}$. See text for details.

The lower limit has been fixed to unity to remove ambiguity about the arbitrary constant of integration and, consequently, the integral is given by the following function of its upper limit:

(14) $$r=\log_{e}\bar{r}=\ln\;\bar{r},$$

as illustrated graphically in Figure 3. The perceived distance r is foreshortened in all radial directions in the perceived visual manifold relative to the corresponding distance $\bar{r}$ in the Euclidean outside space. The amount of foreshortening increases with increasing $\bar{r}$.

It is interesting to notice in Figure 3 that according to the logarithmic relationship the perceived distance r is negative for values of $\bar{r}$ less than one. This does not immediately make intuitive sense because perceived distance should always be a positive number. In fact it implies an anomaly, the existence of a hole about the egocentric origin in $\left(\bar{G},\bar{g}\right)$ that cannot be perceived. This *does* make intuitive sense because we cannot see our own head let alone our own ego. The existence of a hole at the origin has consequences for the perception of areas and volumes containing the origin, but we will not explore that further here.

### 4.2. The Geodesic Spray Field

As defined in Riemannian geometry [109] (Chapter IV), the geodesic spray field,

(15) $$F\left(q,\dot{q}\right)=\left(f_{1}\left(q,\dot{q}\right),f_{2}\left(q,\dot{q}\right)\right)$$

is a second-order vector field in the double-tangent bundle TTG over the tangent bundle TG at each point q=(r, θ, φ) in the perceived visual manifold (G,g) and at each velocity $\dot{q}=\left(\dot{r},\;\dot{\theta },\;\dot{\varphi }\right)$ in the tangent vector space $T_{q}G$ at q∈(G,g). The geodesic spray field is well known in Riemannian geometry and we have previously given a detailed description of it [52]. At each point q=(r, θ, φ) in the manifold (G,g), there exists a tangent space containing the velocity vector (or the direction vector) $\dot{q}=\left(\dot{r},\;\dot{\theta },\;\dot{\varphi }\right)$, and at each point $\dot{q}=\left(\dot{r},\;\dot{\theta },\;\dot{\varphi }\right)$ in the tangent space, there exists a tangent space on the tangent space; that is, a double tangent space. The double tangent space contains the geodesic spray field. It is not a tensor field so it depends on the chosen coordinates and since it can be precomputed as described below it can be regarded as an inherent part of the perceived visual manifold (G,g). It has two parts, $f_{1}\left(q,\dot{q}\right)$ and $f_{2}\left(q,\dot{q}\right)$, known as the horizontal part and vertical part, respectively. The horizontal part $f_{1}\left(q,\dot{q}\right)$ equals the tangential velocity $\dot{q}\in$ $T_{q}G$ in the 3D tangent vector space $T_{q}G$ at each q∈(G,g). The vertical part $f_{2}\left(q,\dot{q}\right)$ provides a measure of the *negative of the illusory acceleration* perceived by a person looking at an object that is actually moving relatively in the outside world at constant speed (the negative sign is explained in the next section).

To illustrate, telegraph poles observed from a car moving at constant speed along a straight road appear not only to loom in size but also to accelerate as they approach. This common example shows that our perceptions of the position, velocity and acceleration of moving objects are distorted in ways consistent with the proposed warped geometry of the visual system. The illusory acceleration $-f_{2}\left(q,\dot{q}\right)$ can be attributed to the metric g in Equations (7) and (12) causing the apparent distance between neighbouring points in (G,g) to appear to increase as the distance r in the Euclidean outside world decreases. Consequently, an object appears to travel through greater distances per unit time as it gets closer to the observer and, therefore, appears to accelerate as it approaches.

The velocity $\dot{q\;}\in T_{q}G$ at each q∈(G,g) is measured relative to the cyclopean coordinate basis vectors $\left(\partial_{r},\partial_{\theta },\partial_{\varphi }\right)$ spanning the 3D tangent vector space $T_{q}G$. However, warping of the perceived visual manifold (G,g) causes these basis vectors $\left(\partial_{r},\partial_{\theta },\partial_{\varphi }\right)$ to change relative to each other from point to point in the manifold. As a result, since the velocity $\dot{q\;}$at each point is measured with respect to these basis vectors, their changes from point to point give rise to apparent changes in the velocity vector $\dot{q\;}$, thereby inducing illusory accelerations. These illusory accelerations do not happen in flat Euclidean space. The negative of the geodesic spray vector $f_{2}\left(q,\dot{q}\right)$ provides a measure of this illusory acceleration at each position and velocity $\left(q,\dot{q}\right)$ in the tangent bundle TG (i.e., union of all the tangent spaces $T_{q}G$ over (G,g)). The geodesic spray field $\left(\dot{q\;},f_{2}\left(q,\dot{q}\right)\right)$ on the tangent bundle TG can be precomputed and can, therefore, be regarded as an inherent part of the perceived visual manifold (G,g).

As shown by Lang [109] and by Marsden and Ratiu [110], the acceleration part $f_{2}\left(q,\dot{q}\right)$ of the geodesic spray field is given by the equation:

(16) $$\langle gf_{2},w\rangle =\frac{1}{2}\langle g^{\prime }w\dot{q,}\;\dot{q}\rangle -\langle g^{\prime }\dot{q}\dot{q,}\;w\rangle ,$$

where 〈·,·〉 represents the metric inner product, $g^{\prime }$ is the Jacobian matrix of the metric g in Equation (7), w is a fixed arbitrary vector, and q=[rθφ]; q˙=[r˙θ˙φ˙]; g=[1r2000cos2φ0001].

Solving Equation (16) for $f_{2}\left(q,\dot{q}\right)$ as a function of position and velocity on the perceived visual manifold (G,g) we obtain:

(17) $$f_{2}\left(q,\dot{q}\right)=\left[\begin{array}{c} f_{r}\\ f_{\theta }\\ f_{\varphi } \end{array}\right]=\left[\begin{array}{c} \frac{{\left(\dot{r}\right)}^{2}}{r}\\ 2\frac{\sin\varphi }{\cos\varphi }\dot{\theta }\dot{\varphi }\\ -\left(\cos\varphi \right)\left(\sin\varphi \right){\dot{\theta }}^{2} \end{array}\right],$$

where ($f_{r},f_{\theta },\;f_{\varphi }$) are the components of $f_{2}\left(q,\dot{q}\right)$ in cyclopean spherical coordinates at each position q and velocity $\dot{q}$ in the tangent bundle TG.

Since, as mentioned above, the geodesic spray field is non-tensorial it follows that it depends on the chosen coordinate basis vectors. When the components ($f_{r},f_{\theta },\;f_{\varphi }$) of the acceleration geodesic spray field $f_{2}\left(q,\dot{q}\right)$ in Equation (17) are recomputed in terms of the tangential velocities $v^{r}$, $v^{\theta }$, and $v^{\varphi }$ in Equation (9) and the metric g(r) in Equation (12), we obtain a different expression:

(18) $$f_{2}\left(q,\dot{q}\right)=\left[\begin{array}{c} f_{r}\\ f_{\theta }\\ f_{\varphi } \end{array}\right]=\left[\begin{array}{c} \frac{{\left(v^{r}\right)}^{2}}{r}-\frac{{\left(v^{\theta }\right)}^{2}}{r}-\frac{{\left(v^{\varphi }\right)}^{2}}{r}\\ 2\frac{v^{r}v^{\theta }}{r}\\ 2\frac{v^{r}v^{\varphi }}{r} \end{array}\right].$$

The illusory accelerations introduced by the warped geometry of the perceived visual space are easier to understand intuitively when presented in terms of the tangential velocities $v^{r}$, $v^{\theta }$, and $v^{\varphi }$ in Equation (18). For example, if $v^{r}=-1$, $v^{\theta }=0$, and $v^{\varphi }=0$, corresponding to an object approaching the observer at constant unit speed along a radial line (like looking out the front window of a train travelling at constant speed along a straight line), the spray acceleration $f_{2}\left(q,\dot{q}\right)$ equals a radial acceleration $\frac{{\left(v^{r}\right)}^{2}}{r}$ directed outward along the radial line. The negative of this is consistent with the object appearing to accelerate as it approaches. If $v^{r}=0$, $v^{\theta }=1$, and $v^{\varphi }=0$, corresponding to an object moving normal to the line of sight (like looking out a side window of the same train), the radial acceleration $f_{2}\left(q,\dot{q}\right)=-\frac{{\left(v^{\theta }\right)}^{2}}{r}$ given by Equation (18) equals the centripetal acceleration required for the object to follow a circular motion with constant tangential velocity centred about the egocentre (consistent with the perceived acceleration of an object seen from a side window of the train at a distance r normal to the train). If $v^{r}=1$, $v^{\theta }=1$, and $v^{\varphi }=0$, corresponding to an object having both radial and tangential components of velocity (like looking ahead but off to one side from the front window of the train), the spray acceleration $f_{2}\left(q,\dot{q}\right)$ given by Equation (18) has a radial component $\frac{{\left(v^{r}\right)}^{2}}{r}-\frac{{\left(v^{\theta }\right)}^{2}}{r}$ and a tangential component $2\frac{v^{r}v^{\theta }}{r}$. The latter equals a coriolis acceleration causing a change in the rate of rotation of the coordinates or direction of gaze. These intuitive descriptions are verified in Section 5 below.

### 4.3. Covariant Derivatives

*Covariant derivatives* defined in Riemannian geometry have an important role to play in visual perception. They provide a quantitative measure of the perceived directional accelerations of objects moving in the outside world taking both actual accelerations and illusory accelerations into account. They also quantify the perceived curvature at each point along the edges of objects and thereby provide the perceived shape of objects as judged from their outlines (see Section 6). Importantly, the perceived rate of change in the nominated direction at the specified point is measured relative to the curvature of the ambient perceived visual manifold at that point. Given a curve α(t) in the manifold (G,g) parameterized by time t, the covariant derivative $\nabla_{\dot{\alpha }}\dot{\alpha }\left(t_{i}\right)$ of the velocity vector $\dot{\alpha }\left(t_{i}\right)$ tangent to the curve in the direction $\dot{\alpha }\left(t_{i}\right)$ at the point $\alpha \left(t_{i}\right)$ is obtained by subtracting the spray acceleration $f_{2}\left(\alpha \left(t_{i}\right),\dot{\alpha }\left(t_{i}\right)\right)$ from the acceleration $\ddot{\alpha }\left(t_{i}\right)$ at the point $\alpha \left(t_{i}\right)$:

(19) $$\nabla_{\dot{\alpha }}\dot{\alpha }\left(t_{i}\right)=\ddot{\alpha }\left(t_{i}\right)-f_{2}\left(\alpha \left(t_{i}\right),\dot{\alpha }\left(t_{i}\right)\right).$$

The acceleration $\ddot{\alpha }\left(t_{i}\right)$ is an ordinary Euclidean acceleration. It does not take into account rotation of the coordinate basis vectors $\left(\partial_{r},\partial_{\theta },\partial_{\varphi }\right)$ from point to point in the manifold, so does not include illusory accelerations. It can be interpreted as a measure of the actual Euclidean acceleration at the corresponding point and direction in the Euclidean outside world.

If the Euclidean acceleration $\ddot{\alpha }\left(t_{i}\right)$ is everywhere zero (i.e., the object is moving at constant speed along a straight line in the environment) then we obtain:

(20) $$\nabla_{\dot{\alpha }}\dot{\alpha }\left(t_{i}\right)=-f_{2}\left(\alpha \left(t_{i}\right),\dot{\alpha }\left(t_{i}\right)\right)$$

at every point $\alpha \left(t_{i}\right)$ along the curve. In this case the covariant derivative $\nabla_{\dot{\alpha }}\dot{\alpha }\left(t_{i}\right)$ is the perceived illusory acceleration equal to the negative of the geodesic spray vector $f_{2}\left(\alpha \left(t_{i}\right),\dot{\alpha }\left(t_{i}\right)\right)$ at each point $t_{i}$ along the curve; i.e., the object appears to accelerate towards the observer as it approaches or decelerate as it recedes. If the Euclidean acceleration $\ddot{\alpha }\left(t\right)$ is not zero and the object is actually accelerating in the Euclidean outside world (most likely following a curved path) then the covariant derivative in Equation (19) gives the perceived metric directional acceleration in the direction $\dot{\alpha }\left(t_{i}\right)$ at each point along the path, taking both the actual Euclidean acceleration and the illusory acceleration into account. It is important to notice that the covariant derivative can be used to measure the *perceived* accelerations across the perceived visual manifold. We will use this fact in subsequent sections.

We can now ask the converse question. How does an object have to move in the Euclidean outside world for it to be perceived as moving at constant speed in a straight line? By setting the perceived acceleration $\nabla_{\dot{\alpha }}\dot{\alpha }\left(t\right)$ equal to zero in Equation (19) we obtain:

(21) $$\ddot{\alpha }\left(t_{i}\right)=f_{2}\left(\alpha \left(t_{i}\right),\dot{\alpha }\left(t_{i}\right)\right).$$

Thus, to be perceived as moving in a straight line at constant metric speed, an object in the Euclidean outside world has actually to be accelerating with a Euclidean acceleration $\ddot{\alpha }\left(t_{i}\right)$ which, at every point along its path in Euclidean space, equals the acceleration spray vector $f_{2}\left(\alpha \left(t_{i}\right),\dot{\alpha }\left(t_{i}\right)\right)$ at the corresponding point and velocity in the perceived visual manifold (G,g). In other words, to be perceived as moving in a straight line at constant speed the object must follow a (usually curved) path in the outside world with a Euclidean acceleration $\ddot{\alpha }\left(t_{i}\right)$ equal but opposite in sign to the illusory acceleration $-f_{2}\left(\alpha \left(t_{i}\right),\dot{\alpha }\left(t_{i}\right)\right)$ introduced by the visual system. This is exactly how the geodesics of the perceived visual manifold (G,g) are defined. The geodesics are accelerating curves in the Euclidean outside world that appear as straight lines with constant metric speed (i.e., their metric acceleration $\nabla_{\dot{\alpha }}\dot{\alpha }\left(t\right)$ equals zero) in the perceived visual manifold (G,g).

The covariant derivative $\nabla_{X}Y\;$where *X* and Y are arbitrary vector fields on (G,g) provides a measure at each point q∈(G,g) of the rate of change of the vector Y for movement in the *X*-direction. In other words, it measures the *directional derivative* of the vector Y in the *X*-direction taking into account the warping of the manifold (G,g) and hence the illusory acceleration. We now use this definition of the covariant derivative to obtain a measure relating to the concept of *parallelity*. At each point along a geodesic α(t) there exists a vector $\dot{\alpha }\left(t\right)$ tangent to the curve. The family of vectors $\dot{\alpha }\left(t\right)$ forms a vector field along the geodesic α(t). Being a geodesic, α(t) has zero metric acceleration ($\nabla_{\dot{\alpha }}\dot{\alpha }\left(t\right)=0$), hence α(t) is perceived as being a constant speed straight line while tangent vectors $\dot{\alpha }\left(t\right)$ along it are perceived as being collinear and are said to be *parallel translated* along α(t).

This notion of parallel translation can be generalized to any family of vectors γ(t) along α(t) not necessarily tangent to the geodesic α(t). As presented by Lang [109], the change in the vector γ(t) for movement in the direction $\dot{\alpha }\left(t\right)$ along α(t) is given by the covariant derivative

(22) $$\nabla_{\dot{\alpha }}\gamma \left(t_{i}\right)=\gamma^{\prime }\dot{\alpha }\left(t_{i}\right)-B\left(\alpha \left(t_{i}\right);\dot{\alpha }\left(t_{i}\right),\gamma \left(t_{i}\right)\right),$$

where $\gamma^{\prime }$ is the Jacobian matrix of the vector γ at each point $\alpha \left(t_{i}\right)$ along the curve and $B\left(\alpha \left(t_{i}\right);\dot{\alpha }\left(t_{i}\right),\gamma \left(t_{i}\right)\right)$ is a symmetrical bilinear map at $\alpha \left(t_{i}\right)$ that transforms the two vectors $\dot{\alpha }\left(t_{i}\right)$ and $\gamma \left(t_{i}\right)$ in the 3D tangent vector space $T_{\alpha \left(t_{i}\right)}G$ at the point $\alpha \left(t_{i}\right)$ along the curve into an acceleration vector in $T_{\alpha \left(t_{i}\right)}G$. The bilinear map B is algebraically related to the geodesic spray field $f_{2}$ and, as shown in Equations (23) and (24), given one the other can be computed [109]:

(23) $$B\left(\alpha \left(t_{i}\right);\dot{\alpha }\left(t_{i}\right),\gamma \left(t_{i}\right)\right)=\frac{1}{2}\left[f_{2}\left(\alpha \left(t_{i}\right),\dot{\alpha }\left(t_{i}\right)+\gamma \left(t_{i}\right)\right)-f_{2}\left(\alpha \left(t_{i}\right),\dot{\alpha }\left(t_{i}\right)\right)-f_{2}\left(\alpha \left(t_{i}\right),\gamma \left(t_{i}\right)\right)\right],$$

(24) $$f_{2}\left(\alpha \left(t_{i}\right),\dot{\alpha }\left(t_{i}\right)\right)=B\left(\alpha \left(t_{i}\right);\dot{\alpha }\left(t_{i}\right),\dot{\alpha }\left(t_{i}\right)\right).$$

When the covariant derivative $\nabla_{\dot{\alpha }}\gamma \left(t\right)$ in Equation (22) is zero everywhere along the curve α(t) the vector γ(t) is said to be parallel translated along α(t) and all the vectors γ(t) along the curve are parallel to each other. Parallel translation of the vector $\gamma \left(t_{0}\right)$ at $\alpha \left(t_{0}\right)$ to the vector $\gamma \left(t_{i}\right)$ at $\alpha \left(t_{i}\right)$ along α(t) is path dependent and is described by $P_{t_{0}}^{t_{i}}\gamma \left(t_{0}\right)=\gamma \left(t_{i}\right)\;$where $P_{t_{0}}^{t_{i}}$ is a linear invertible isometric transformation between the vector spaces $T_{\alpha \left(t_{0}\right)}G$ and $T_{\alpha \left(t_{i}\right)}G$ along the curve.

Defined in this way, parallel transformation has a useful role to play in understanding visual perception. This is because vectors γ(t) that are parallel translated along a geodesic in the visual manifold will be perceived as being parallel to each other, whereas they are not parallel in the Euclidean outside world. The generalized covariant derivative $\nabla_{\dot{\alpha }}\gamma \left(t\right)$ of vector field γ(t) along α(t) and the associated parallel translation of γ(t) along α(t) when $\nabla_{\dot{\alpha }}\gamma \left(t\right)=0$ can be used to quantify the difference between lines in the outside world that are truly parallel and lines that are perceived as being parallel. This underlies the experimental work of Cuijpers and colleagues [17,18,19,20] introduced in Section 1 and discussed in Section 8.5.

### 4.4. Christoffel Symbols

To simplify notation in this section and the next we implement a number-indexing system that equates *r* with 1, θ with 2, and φ with 3. Thus, for example, the cyclopean coordinate basis vectors $\partial_{r},\partial_{\theta },$ and $\partial_{\varphi }$ spanning a tangent vector space will be written in the alternative form $\partial_{k}$ with *k* = 1, 2, 3 and we introduce the *Christoffel symbols* notated as $\Gamma_{jk}^{i}$ with i,j,k=1, 2, 3. The Christoffel symbols $\Gamma_{jk}^{i}$ are important in vision because they allow us to quantify the relative directional rates of change of the coordinate basis vectors $\partial_{k}$ that occur with infinitesimal movements in the warped visual manifold (G,g). They are related to the covariant derivatives $\nabla_{\partial_{k}}\partial_{j}$ with j,k=1, 2, 3 measuring the rate of change of each coordinate basis vector $\partial_{j}$ associated with movement in the direction of another coordinate basis vector $\partial_{k}$ at each point q in the manifold (G,g). As indicated in Section 4.2, it is this change in the coordinate basis vectors with movement across the manifold that gives rise to illusory accelerations of objects moving in the outside world. It should not be a surprise, therefore, that the Christoffel symbols provide an alternative way of quantifying the components ${\left(f_{2}\left(q,\dot{q}\right)\right)}^{i}=-{\dot{q}}^{k}{\dot{q}}^{j}\Gamma_{jk}^{i}$ of the acceleration spray vector $f_{2}\left(q,\dot{q}\right)$ at each position and velocity $\left(q,\dot{q}\right)$. The repetition of the indices j and k first as superscripts and then as subscripts in this equation implies summation over j,k=1, 2, 3. Known as Einstein’s summation convention, it is used from here forward in this paper, particularly in Section 4.5. There we introduce tensors which operate on dual spaces of vectors and covectors (analogously to matrices operating on dual spaces of column and row vectors). To facilitate use of the summation convention, index positions are always chosen so that vectors have lower indices and covectors have upper indices while the components of vectors have upper indices and those of covectors have lower indices. This ensures that the Einstein summation convention can always be applied. This simplifies notation by removing the need for summation signs. We require the Christoffel symbols in order to compute the Riemann curvature at each point in the manifold in Section 4.5.

At each q∈(G,g), the Christoffel symbols $\Gamma_{jk}^{i}$ correspond to the components of the covariant derivative vector $\nabla_{\partial_{k}}\partial_{j}$ in the tangent space projected on to the basis vectors spanning the tangent space at that point; that is:

(25) $$\nabla_{\partial_{k}}\partial_{j}=\sum_{i=1}^{3}\Gamma_{jk}^{i}\partial_{i}=\Gamma_{jk}^{1}\partial_{1}+\Gamma_{jk}^{2}\partial_{2}+\Gamma_{jk}^{3}\partial_{3}=\Gamma_{jk}^{i}\partial_{i}.$$

Notice the use of the Einstein summation convention in the last term in Equation (25). Working in this way generates a large number of components $\Gamma_{jk}^{i}$, i.e., 27 Christoffel symbols are required at each point q=(r, θ, φ) in the 3D perceived visual manifold (G,g). Nevertheless, these have the advantage that they can be computed at each point from the known metric g(r) and its differentials. By definition, in any Riemannian manifold the Christoffel symbols are compatible with the Riemannian metric, that is ∇g=0, and are symmetrical, that is $\Gamma_{jk}^{i}=\Gamma_{kj}^{i}$. From these properties the following equation expressing the Christoffel symbols at each point in terms of the metric g(r) and its differentials is derived [111]:

(26) $$\Gamma_{jk}^{i}=\frac{1}{2}g^{im}\left(\frac{\partial g_{mj}}{\partial x^{k}}+\frac{\partial g_{mk}}{\partial x^{j}}-\frac{\partial g_{jk}}{\partial x^{m}}\right),$$

where $g_{mj}$ are the components of g at each q∈(G,g) and $g^{im}$ are the components of the inverse metric $g^{-1}$ at each q∈(G,g).

Using Equation (26) and the Riemannian metric in Equation (12), we computed all 27 Christoffel symbols for the perceived visual manifold (G,g) as a function of their position q=(r, θ, φ) in (G,g). We found all to be zero except for the following seven:

(27) $$\Gamma_{11}^{1}=-\frac{1}{r},{\;\Gamma }_{22}^{1}=\frac{1}{r},{\;\Gamma }_{33}^{1}=\frac{1}{r},{\;\Gamma }_{12}^{2}=-\frac{1}{r},{\;\Gamma }_{21}^{2}=-\frac{1}{r},\;\Gamma_{13}^{3}=-\frac{1}{r},{\;\Gamma }_{31}^{3}=-\frac{1}{r}.$$

Returning to (r, θ, φ) notation, the subparts of Equation (27) show respectively:

(i) ∇∂r∂r=(−1r)∂r+0∂θ+0∂φ so only the component of $\nabla_{\partial_{r}}\partial_{r}$ in the $\partial_{r}$ direction is non-zero,

(ii) ∇∂θ∂θ=(1r)∂r+0∂θ+0∂φ so only the component of $\nabla_{\partial_{\theta }}\partial_{\theta }$ in the $\partial_{r}$ direction is non-zero,

(iii) ∇∂φ∂φ=(1r)∂r+0∂θ+0∂φ so only the component of $\nabla_{\partial_{\varphi }}\partial_{\varphi }$ in the $\partial_{r}$ direction is non-zero,

(iv and v) ∇∂r∂θ=∇∂θ∂r=0∂r−(1r)∂θ+0∂φ so only the components of $\nabla_{\partial_{r}}\partial_{\theta }\;\mathrm{and}\;\;\nabla_{\partial_{\theta }}\partial_{r}$ in the $\partial_{\theta }$ direction are non-zero, and (vi and vii) ∇∂r∂φ=∇∂φ∂r=0∂r+0∂θ−(1r)∂φ so only the components of $\nabla_{\partial_{r}}\partial_{\varphi }\;\mathrm{and}\;\nabla_{\partial_{\varphi }}\partial_{r}$ in the $\partial_{\varphi }$ direction are non-zero. We now use this information to compute the curvature of the perceived visual manifold (G,g).

### 4.5. The Riemann Curvature Tensor

Warping of the perceived visual manifold (G,g) is quantified by the Riemann curvature tensor at each point q=(r, θ, φ) in the manifold. However, while the curvature of a 2D surface is an easily understood concept, the idea of the curvature of a 3D manifold is more difficult to grasp. Hence we provide the following intuitive description. A key property of the 3D Euclidean outside world is that it is “flat” with zero curvature everywhere. Consequently, an arbitrary tangent vector can be parallel translated along any pathway between any two points and remain parallel everywhere. In other words, in the Euclidean world parallel translation is path independent and the flat space is said to be *totally parallel*. Given arbitrary vector fields X and Y on the flat Euclidean space, parallel translation of a vector Z for an infinitesimal time along the integral flow of Y followed by parallel translation for an infinitesimal time along the integral flow of X does not in general equal parallel translation of the vector Z for an infinitesimal time along the integral flow of X followed by parallel translation for an infinitesimal time along the integral flow of Y. Equivalently, in terms of covariant derivatives, $\nabla_{X}\nabla_{Y}Z-\nabla_{Y}\nabla_{X}Z\neq 0$, and we can say that in general the products of covariant derivatives do not commute, even on flat Euclidean spaces. However, by definition, $\left(\nabla_{X}\nabla_{Y}-\nabla_{Y}\nabla_{X}\right)Z$ equals $\nabla_{\left[X,Y\right]}Z$, where [X,Y]=(XY−YX) is a vector known as the *Lie bracket*. Thus, for arbitrary vector fields X and Y and an arbitrary vector Z on a flat Euclidean space, we can write that $\nabla_{X}\nabla_{Y}Z-\nabla_{Y}\nabla_{X}Z-\nabla_{\left[X,Y\right]}Z=0$. This provides a criterion for flatness. Any other space for which $\nabla_{X}\nabla_{Y}Z-\nabla_{Y}\nabla_{X}Z-\nabla_{\left[X,Y\right]}Z$ does not equal zero is not flat. Indeed we can define a curvature operator $R\left(X,Y\right)=\nabla_{X}\nabla_{Y}-\nabla_{Y}\nabla_{X}-\nabla_{\left[X,Y\right]}$ that operates on a vector Z to give a vector $R\left(X,Y\right)Z=\nabla_{X}\nabla_{Y}Z-\nabla_{Y}\nabla_{X}Z-\nabla_{\left[X,Y\right]}Z$ at every point across a manifold. If R(X,Y)Z is not zero at a point then the manifold is not flat at that point and the magnitude of the vector R(X,Y)Z provides a measure of how far the curvature deviates from flatness.

Similar to the curvature operator R(X,Y) operating on a vector Z across a manifold, we now define a *curvature tensor* that quantifies curvature as a real number independently of the coordinates in which a manifold is expressed. Using the tensor characterization lemma [57] (Chapter 2) and by introducing a covector ω (i.e., a dual vector similar to a row vector in linear matrix theory) at each point in the manifold, we can define a type (1,3) curvature tensor that operates on three vectors X,Y,Z and a covector ω and transforms them into a real number:

(28) R(X,Y,Z,ω)=〈R(X,Y)Z,ω〉=a real number.

The vectors X,Y,Z can be written as linear combinations $X=X^{i}\partial_{i}$, $Y=Y^{j}\partial_{j}$, Z= $Z^{k}\partial_{k}$ of the cyclopean coordinate basis vectors $\partial_{r},\partial_{\theta },$ and $\partial_{\varphi }$ spanning the tangent space $T_{q}G$ at the point q∈(G,g) and the covector ω can be written as a linear combination $\omega =\;\omega_{l}\;dx^{l}$ of the dual basis vectors $\left(dx^{1}=dr,\;dx^{2}=\;d\theta ,dx^{3}=\;d\varphi \right)$ spanning the covector space $T_{q}^{*}G$ at q∈(G,g). Notice the use of upper and lower indices consistent with the Einstein summation convention (Section 4.4). The tensor R(X,Y,Z,ω) can be represented as a tensor in an 81-dimensional tensor space spanned by basis tensors $\left(dx^{i}⊗dx^{j}⊗dx^{k}⊗\partial_{l}\right)$ (⊗ signifies tensor product) with components ${R_{ijk}}^{l}$ quantifying the projection onto each basis tensor. The tensor R(X,Y,Z,ω) can then be expressed in terms of its 3×3×3×3=81 components,

(29) $${R_{ijk}}^{l}=R\left(\partial_{i},\partial_{j},\partial_{k},dx_{l}\right),i,j,k,l=1,\;2,\;3$$

at each q∈(G,g). The components ${R_{ijk}}^{l}$ can be computed [111] from the previously obtained Christoffel symbols in Equation (27) using the equation:

(30) $${R_{ijk}}^{l}=\frac{\partial \Gamma_{jk}^{l}}{\partial x^{i}}-\frac{\partial \Gamma_{ki}^{l}}{\partial x^{j}}+\Gamma_{jk}^{m}\Gamma_{mi}^{l}-\Gamma_{ki}^{m}\Gamma_{mj}^{l}.$$

Applying Equation (30), we computed all 81 components ${R_{ijk}}^{l}$ (i,j,k,l=1,2,3) of the curvature tensor R(X,Y,Z,ω) as a function of position q= (r, θ,φ) in (G,g). We then implemented a useful conversion that entails expressing the tensor R(X,Y,Z,ω) in the form $R_{m}\left(X,Y,Z,W\right)$ where W is the vector in $T_{q}G$ dual to the covector ω in $T_{q}^{*}G$. The type (0,4) Riemann curvature tensor $R_{m}\left(X,Y,Z,W\right)$ can be represented as a tensor in an 81-dimensional tensor space spanned by basis tensors $\left(dx^{i}⊗dx^{j}⊗dx^{k}⊗dx^{l}\right)$ with components $R_{ijkl}$ (i,j,k,l=1,2,3) quantifying the projection onto each basis tensor. The Riemann curvature tensor $R_{m}\left(X,Y,Z,W\right)$ can then be expressed in terms of its 3×3×3×3=81 components,

(31) $$R_{ijkl}=R\left(\partial_{i},\partial_{j},\partial_{k},\partial_{l}\right),i,j,k,l=1,\;2,\;3$$

at each q∈(G,g) by the operation known as *lowering the index*. This is achieved using the important property of Riemannian metrics g that allows us to convert tangent vectors to cotangent vectors and vice versa; viz., gW=ω and $\omega =g^{-1}W$ [57] (Chapter 2). In terms of coordinate basis vectors $\partial_{r},\partial_{\theta },\partial_{\varphi }$ spanning the tangent space $T_{q}G$ at the point q∈(G,g) and coordinate basis covectors $dx^{1}=dr,\;dx^{2}=\;d\theta ,\;dx^{3}=\;d\varphi$ spanning the dual cotangent space $T_{q}^{*}G$ at the same point q∈(G,g) this conversion can be written as $g_{ll}\left(W^{l}\partial_{l}\right)=\omega_{l}dx^{l}$. In other words, the components convert according to $g_{ll}W^{l}=\omega_{l}$. This is called *lowering the index on the components*. Applying this between components ${R_{ijk}}^{l}$ and $R_{ijkl}$ of the curvature tensors gives:

(32) $$g_{ll}R_{ijk}^{\;\;\;\;\;\;l}=R_{ijkl}\left(i,j,k,l=1,2,3\right)$$

where $g_{ll}=\frac{1}{r^{2}}$ from Equation (12). The advantage of lowering the index on the curvature components ${R_{ijk}}^{l}$ in this way to obtain components $R_{ijkl}$ is that curvature components $R_{ijkl}$ are known to possess symmetries $R_{ijkl}=-R_{jikl}$, $R_{ijkl}=-R_{ijlk}$, $R_{ijkl}+\;R_{jkil}+R_{kijl}=0$, and $R_{ijkl}=R_{klij}$. These symmetries allow the number of components to be greatly reduced. Indeed, for the 81 components ${R_{ijk}}^{l}$ computed using Equation (30) we find that all components $R_{ijkl},\;i,j,k,l=1,2,3$ of the Riemann curvature tensor $R_{m}\left(X,Y,Z,W\right)$ at each point q∈(G,g) are zero with the exception of the following three:

(33) $$R_{1221}=R_{r\theta \theta r}=-\frac{1}{r^{4}};\;R_{1331}=R_{r\varphi \varphi r}=-\frac{1}{r^{4}};\;R_{2332}=R_{\theta \varphi \varphi \theta }=-\frac{1}{r^{4}};$$

where r is the Euclidean cyclopean distance from the egocentre. As described in the next section, these three nonzero components of the Riemann curvature tensor are known as *sectional curvatures* of (G,g). In Section 5 we examine these sectional curvatures by means of geodesic simulations.

## 5. Geodesics of the Perceived Visual Manifold

### 5.1. Simulations

Geodesics plotted in the outside world are accelerating pathways (most often curved) that are perceived as constant speed straight lines. Thus, the Euclidean accelerations $\ddot{\alpha }\left(t_{i}\right)=f_{2}\left(\alpha \left(t_{i}\right),\dot{\alpha }\left(t_{i}\right)\right)$ of geodesic trajectories in the outside world quantify the compressions and stretchings of the perceived visual space introduced by the visual system. Plotting families of geodesic trajectories in the outside world provides a method for visualizing and quantifying the warped geometry of the perceived visual manifold. For example, roughly speaking, neighbouring geodesics that diverge in the outside world indicate a negative curvature (bowl shape) of the manifold (G,g) while neighbouring geodesics that converge in the outside world indicate a positive curvature of (G,g).

**Figure 4:**
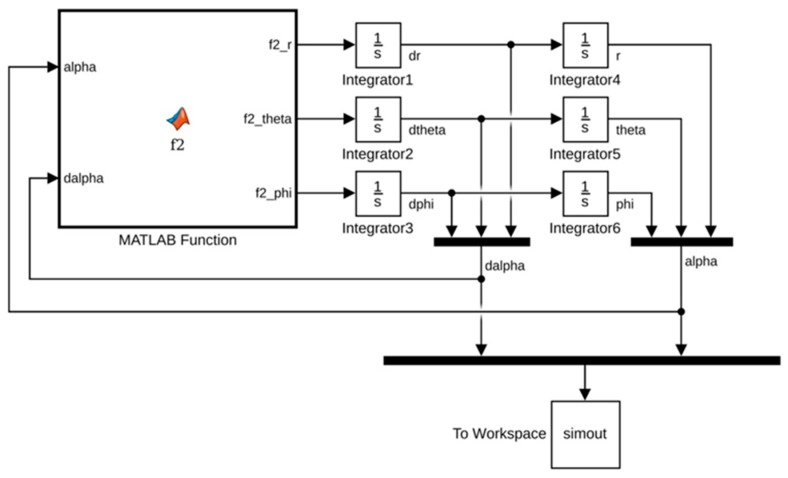
A block diagram for the Matlab/Simulink simulator used to generate geodesic trajectories in the 3D Euclidean outside world given initial conditions α(0)=(r(0), θ(0), φ(0)) and $\dot{\alpha }\left(0\right)=\left(\dot{r}\left(0\right),\;\dot{\theta }\left(0\right),\;\dot{\varphi }\left(0\right)\right)$ set equal to (r(0),theta(0),phi(0) ) and (dr(0),dtheta(0),dphi(0) ) in the diagram. The MATLAB Function block was programmed to evaluate the expression for $f_{2}\left(\alpha \left(t_{i}\right),\dot{\alpha }\left(t_{i}\right)\right)$ in Equation (17). For each run the geodesic trajectory alpha = (r,theta,phi) was stored in the workspace, converted to Cartesian coordinates and plotted as shown in Figure 5, Figure 6 and Figure 7 below.

Geodesic trajectories are plentiful. Like straight lines in Euclidean space they pass through every point in every direction. Given an initial position α(0)=(r(0),θ(0),φ(0)) and an initial velocity $\dot{\alpha }\left(0\right)=\left(\dot{r}\left(0\right),\dot{\theta }\left(0\right),\dot{\varphi }\left(0\right)\right)$ at t=0 the geodesic trajectory α(t) in the outside world parameterized by time t can be computed by solving the non-linear differential equations Equations (17) and (21). To solve these non-linear equations we constructed the Matlab/Simulink simulator shown in Figure 4. We call the simulator a *geodesic trajectory generator* (GTG). By appropriately setting initial conditions α(0)=(r(0),θ(0),φ(0)) and $\dot{\alpha }\left(0\right)=\left(\dot{r}\left(0\right),\dot{\theta }\left(0\right),\dot{\varphi }\left(0\right)\right)$ in the GTG, it can generate geodesic trajectories emanating from any initial point with any initial velocity in any initial direction in the 3D Euclidean outside world.

A useful way to examine the warped geometry of the perceived visual manifold is to plot families of geodesics in the 3D outside world emanating from different initial points q=α(0) with a family of initial velocities $v_{i}=\dot{\alpha }\left(0\right)$ confined to a chosen 2D plane at each initial point in the outside world. The nature of the warping of the manifold will determine the pattern of geodesics that result for each initial point and each initial plane containing the initial velocities. Such families of geodesics can be constructed using the following procedure: Choose an initial point q=α(0)=(r(0),θ(0),φ(0)) in the outside world. At this initial point q, specify two orthonormal vectors $e_{1}$ and $e_{2}$ that span a chosen 2D plane in the 3D tangent space at the initial point. This is denoted as *plane II*. Construct a family of equally spaced unit-length initial velocity vectors say $v_{i},\;i=0,1,\cdots ,35$ from 0 deg to 350 deg in the plane II with angular spacing of 10 deg. Use q and each initial velocity $v_{i},\;i=0,1,\cdots ,35$ as initial conditions $\left(\alpha \left(0\right),\dot{\alpha }\left(0\right)\right)$ in the GTG simulator to generate a family of geodesic trajectories α(t) in the 3D outside world emanating from the initial point q=α(0) in every direction determined by the initial velocity vectors $\dot{\alpha }\left(0\right)=v_{i},\;i=0,1,\cdots ,35$ in the initial velocity plane II at the initial point.

The resulting family of geodesics α(t) emanating from the initial point q=α(0) sweep out a 2D surface in the 3D Euclidean outside world denoted by $S_{II}$ called the *plane section determined by the plane II.* As derived in Appendix B, due to this method for constructing $S_{II}$, every geodesic in the ambient manifold (G,g) emanating from the point q=α(0) with initial velocity tangent to the plane II is also a geodesic emanating from the point q=α(0) in the 2D submanifold $S_{II}$. Consequently the component $R_{e_{1}e_{2}e_{2}e_{1}}$ of the Riemann curvature tensor at the initial point q=α(0) is both the *sectional curvature* $K\left(e_{1},e_{2}\right)=R_{m}\left(e_{1},e_{2},e_{2},e_{1}\right)$ of the perceived visual manifold (G,g) at q=α(0) and the *Gaussian curvature* $\tilde{K}\left(e_{1},e_{2}\right)={\tilde{R}}_{m}\left(e_{1},e_{2},e_{2},e_{1}\right)$ of the 2D plane section $S_{II}$ at q=α(0). Thus the non-zero curvature components $R_{r\theta \theta r},\;R_{r\varphi \varphi r}\;\mathrm{and}\;R_{\theta \varphi \varphi \theta }$ computed in Section 4.5 measure both the sectional curvatures of the manifold (G,g) and the Gaussian curvatures of the plane sections $S_{II}$ determined by the planes II spanned respectively by the pairs of coordinate basis vectors $\partial_{r}\partial_{\theta }$, $\partial_{r}\partial_{\varphi }$, and $\partial_{\theta }\partial_{\varphi }$ at q=α(0).

Using the GTG simulator we generated families of geodesics with different initial points and initial velocity planes in the 3D outside Euclidean world. The GTG shown in Figure 4 uses the geodesic spray field given in Equation (17). (In theory we might expect a GTG using the geodesic spray field given in Equation (18) to work equally well. However, in practice such a simulator proved unsatisfactory because some of the geodesics it produced were distorted by a singularity at θ=π2 rad, φ=π2 rad.)

**Figure 5:**
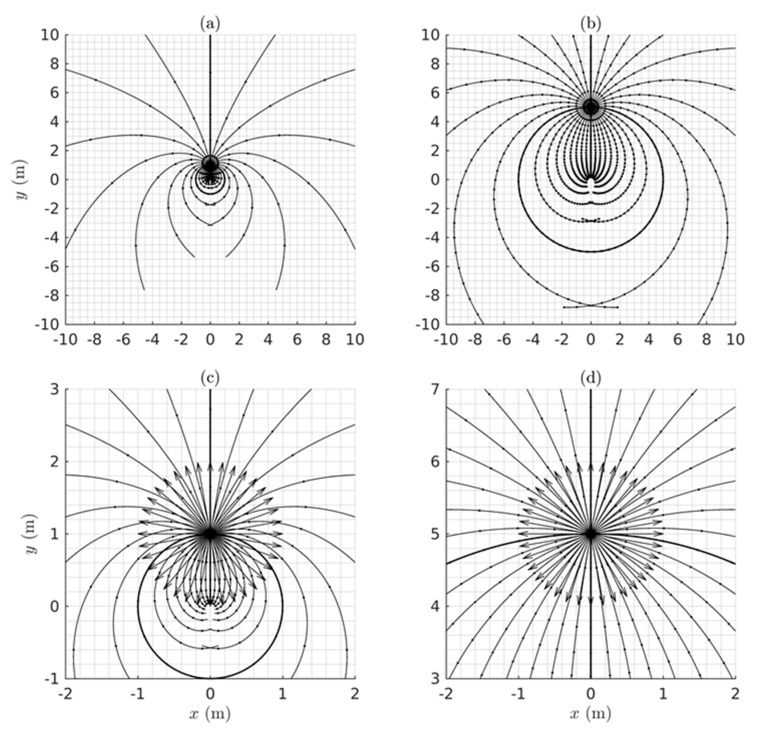
Cartesian plots in the Euclidean outside world of geodesics emanating from two different initial points in the horizontal xy-plane passing through the egocentre at (0, 0, 0) with unit-length initial velocities at the initial point set in the xy-plane. These geodesics all remain confined to the xy-plane where they form radial, circular, or spiral lines from the initial point (see text for detail). The radial and circular geodesics have been slightly thickened in all four diagrams. Dots along geodesics mark 500 ms intervals of time. The same plots are obtained for any plane passing through the egocentre. (**a**) A family of 36 geodesics emanating from initial point (x = 0 m, y = 1 m, z = 0 m) generated from 36 unit-length initial velocity vectors set in the xy-plane and equally spaced in all directions from the initial point. (**b**) A family of 36 geodesics emanating from initial point (x = 0 m, y = 5 m, z = 0 m) generated from 36 unit-length initial velocity vectors as in (a). (**c**) A magnified view of the initial point in (a) showing the family of 36 unit-length initial velocity vectors set in the xy-plane at that point together with their corresponding geodesics. (**d**) A magnified view of the initial point in (b) showing the family of 36 unit-length initial velocity vectors set in the xy-plane at that point together with their corresponding geodesics.

**Figure 6:**
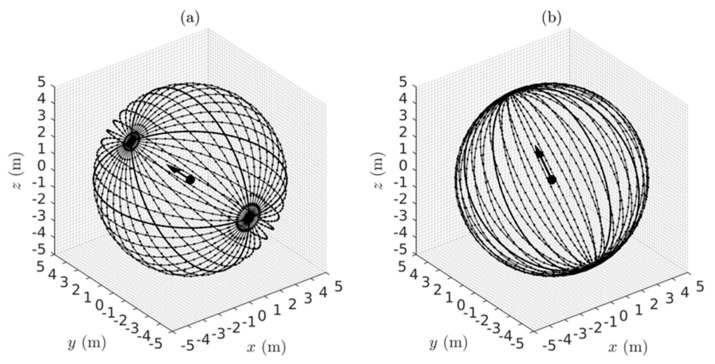
Cartesian plots in the Euclidean outside world of geodesics emanating from two different initial points with 36 unit-length initial velocity vectors set in a plane at the initial point normal to the radial line (indicated by the arrow) connecting it to the egocentre (0, 0, 0) (indicated by the dot). Small dots along the geodesics mark 500 ms intervals of time. (**a**) A family of 36 geodesics emanating from initial point (x = 0 m, y = 5 m, z = 0 m) (same as initial point in Figure 5b) generated from 36 unit-length initial velocity vectors set in the xz-plane passing through the initial point and equally spaced in all directions from that point in the plane. The resulting 36 geodesics do not remain in the xz-plane but become constant tangential speed longitude lines emanating from the initial point to form a 5 m radius sphere centred on the egocentre. The circle geodesic in the xy-plane in Figure 5b can be seen in Figure 6a and is slightly thickened. (**b**) A family of 36 geodesics emanating from initial point (x = 0 m, y = 3.54 m, z = 3.54 m). The 36 unit-length equally spaced initial velocity vectors are set in the plane normal to the line connecting the egocentre and the initial point (a plane tilted by π4 rad to the horizontal and tangent to the sphere at that initial point). The resulting 36 geodesics do not remain in that plane but become constant tangential speed longitude lines emanating from the initial point to form a 5 m radius sphere centred on the egocentre with its axis between the egocentre and the initial point tilted by π4 rad. The spheres generated in (a) and (b) correspond to visual spheres centred on the egocentre.

We found that the resulting plane sections $S_{II}$ corresponding to the families of generated geodesics can be characterized by three categories related to the three nonzero sectional curvatures in Equation (35). Together these three categories of plane sections $S_{II}$ provide a complete picture of the warped geometry of the perceived visual manifold and of its sectional curvatures $R_{r\theta \theta r},\;R_{r\varphi \varphi r}\;\mathrm{and}\;R_{\theta \varphi \varphi \theta }$. The geodesics sweeping out each of the three types of $S_{II}$ are presented in the figures below where, for convenience of plotting, the trajectories α(t)=(r(t),θ(t),φ(t)) are converted to Cartesian coordinates α(t)=(x(t),y(t),z(t)). As a consequence, in Figure 5 the $\partial_{r}\partial_{\theta }$-plane is depicted as the xy-plane and the $\partial_{r}\partial_{\varphi }$-plane is depicted as the yz-plane. Likewise, in Figure 6 the $\partial_{\theta }\partial_{\varphi }$-plane through the initial point is depicted as a plane in (x, y, z)-space tangent to the sphere passing through initial point α(0)=(x(0),y(0),z(0)).

### 5.2. Initial Planes II Passing through the Egocentre

The simulations show that the family of geodesics $S_{II}$ emanating from any initial point q in the outside world at Euclidean distance r from the egocentre with initial velocities $v_{i},\;i=0,1,\cdots ,35$ set within any plane II *passing through the egocentre* remain confined to that plane for all time. In other words, each plane passing through the egocentre is *totally geodesic* so that any point in the plane can be connected to any other point in the plane by a geodesic confined to the plane. Such planes include the $\partial_{r}\partial_{\theta }$-plane, the $\partial_{r}\partial_{\varphi }$-plane, or any rotation of these planes through any rotation angle ψ about the $\partial_{r}$-axis, the $\partial_{\theta }$-axis or the $\partial_{\varphi }$-axis passing through the origin.

Figure 5 (plotted in equivalent Cartesian coordinates) illustrates the above result. It shows two families of geodesics, one emanating from initial point q=(r(0)=1 m, θ(0)=π2 rad, φ(0)=0 rad) (i.e., x = 0 m, y = 1 m, z = 0 m) and the other from initial point q=(r(0)=5 m, θ(0)=π2 rad, φ(0)=0 rad) (i.e., x = 0 m, y = 5 m, z = 0 m) in the outside world. In each case unit-length initial velocities $v_{i}$, i=0,1,⋯,35 are set in the $\partial_{r}\partial_{\theta }$-plane (i.e., the horizontal xy-plane passing through the egocentre) at the initial point. All the geodesics in 5a and 5b (magnified in 5c and 5d) are contained for all time within the $\partial_{r}\partial_{\theta }$-plane passing through the egocentre in the Euclidean outside world where they appear as accelerating or decelerating trajectories. When the initial unit tangential velocity v at the initial point q is aligned with the $\partial_{r}$-axis (i.e., the y-axis), the geodesic follows a straight-line pathway along the axis, either accelerating outwards away from the egocentre or decelerating inwards towards it. When the initial tangential velocity v at the initial point q is aligned with the $\partial_{\theta }$-axis, the geodesic follows a constant tangential speed circular pathway (circular geodesic) in the outside world centred on the egocentre. When the initial tangential velocity v at the initial point q is at any other angle in the $\partial_{r}\partial_{\theta }$-plane, the geodesic follows a spiral pathway in the outside world. When the initial velocity v has an outward pointing component, the geodesic follows an accelerating outward spiral while when it has an inward pointing component, the geodesic follows a decelerating inward spiral. In contrast, if plotted in the perceived visual manifold (G,g) all these geodesics would appear as constant speed straight lines confined to the $\partial_{r}\partial_{\theta }$-plane through the egocentre.

All the geodesics in Figure 5 diverge from each other indicating that the horizontal plane through the egocentre in the perceived visual manifold (G,g) is negatively curved. Indeed, all the planes passing through the egocentre are negatively curved. Comparing 5a with 5b shows that the deviation between neighbouring geodesics decreases as the Euclidean distance r increases. This is consistent with the negative sectional curvature $R_{r\theta \theta r}=R_{1221}=-\frac{1}{r^{4}}$ in Equation (33) computed in Section 4.5 decreasing with increasing Euclidean distance r. The 500 ms spacing between the dots in 5a compared with 5b shows that the acceleration along outward spiralling geodesics is greater in the near visual field (Figure 5a) than in the far visual field (Figure 5b). This is consistent with objects appearing to accelerate as they approach. Also, comparing 5c and 5d, the deviation between the initial velocity vectors and the corresponding geodesics is greater in the near field than in the far field. The same pattern of geodesics is obtained for all planes passing through the egocentre. This includes the vertical $\partial_{r}\partial_{\varphi }$-plane, consistent with the negative sectional curvature $R_{r\varphi \varphi r}=R_{1331}=-\frac{1}{r^{4}}$ in Equation (33) computed in Section 4.5. The logarithmic spiral structure of the simulated geodesics in Figure 5 is the same as the geodesic structure described by Koenderink and van Doorn [27] for *frontally centred visual fields* with a well-defined “primary visual direction” and a specialized fovea.

### 5.3. Initial Planes II Normal to the Radial line from the Egocentre to the Initial Point

The simulations show that the family of geodesics $S_{II}$ emanating from any initial point q in the outside world at any Euclidean distance r from the egocentre with unit-length initial velocities $v_{i}$, i=0,1,⋯,35 set in a plane II normal to a radial line from the egocentre (i.e., any $\partial_{\theta }\partial_{\varphi }$-plane normal to a radial line) does NOT remain confined to that initial velocities plane. This plane is illustrated by the two families of geodesics (plotted in equivalent Cartesian coordinates) in Figure 6. Figure 6a shows a family of geodesics emanating from initial point q=(r(0)=5 m, θ(0)=π2 rad, φ(0)=0 rad) (i.e., x = 0 m, y = 5 m, z = 0 m) in the outside world (same point as in Figure 5b) but with initial velocities $v_{i}$, i=0,1,⋯,35 set in the $\partial_{\theta }\partial_{\varphi }$-plane at the initial point normal to the radial line (i.e., the xz-plane passing through the initial point). All the geodesics in Figure 6a follow constant tangential speed great circle pathways (like longitude lines emanating from the north pole) on the sphere centred on the egocentre and passing through the initial point q in the outside world. A similar family of great circle geodesics is shown in Figure 6b, this time for the initial point q=(r(0)=5 m, θ(0)=π2 rad, φ(0)=π4 rad) (i.e., x = 0 m, y = 3.54 m, z = 3.54 m) in the outside world. Again, the initial velocities are set in the $\partial_{\theta }\partial_{\varphi }$-plane at the initial point normal to the radial line, now a plane tilted by π4 radians from the xy-plane. Again, these geodesics follow constant tangential speed great circle pathways on the 5 m radius sphere centred on the egocentre and passing through the initial point q in the outside world but now the axis of the sphere is tilted by π4 radians towards the egocentre. While the simulated geodesics extend all the way around the egocentre as shown in Figure 6, a human observer has only a forward-looking visual field so can see only a portion of the sphere. However, the direction of gaze can be varied in any direction and so the entire sphere can be experienced via visual scanning and a series of fixations.

### 5.4. Initial Planes II Not Normal to the Radial Line from the Egocentre to the Initial Point and Not Passing Through the Egocentre

**Figure 7:**
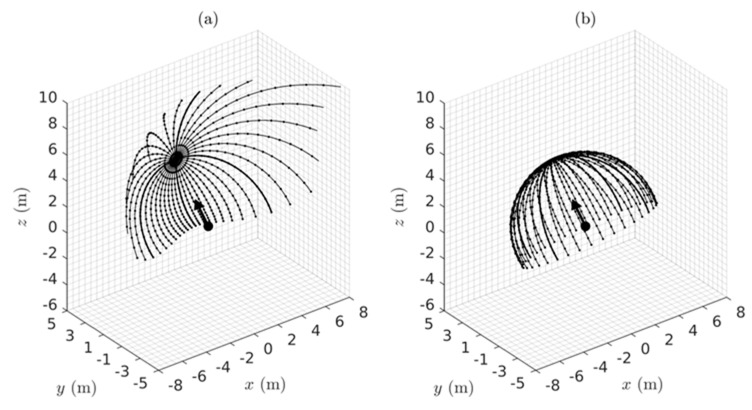
Cartesian plots in the Euclidean outside world comparing geodesics with unit-length initial velocity vectors set in a plane at the initial point not normal to the radial line to the egocentre (0, 0, 0) with those where the velocity plane is normal. The radial line is indicated by an arrow, the egocentre by a dot and small dots along the geodesics mark 500 ms intervals of time. (**a**) Geodesics emanating from the initial point (x = 0 m, y = 3.54 m, z = 3.54 m) with a family of 36 unit-length initial velocity vectors set in the xz-plane passing through the initial point. This plane is tilted back by an angle of π4 rad from the normal to the radial line. The resulting family of geodesics is comprised of a weighted sum of two parts: (i) Spherical geodesics associated with initial velocity vectors projected into the plane normal to the radial line as in Figure 6b. (ii) Spiral geodesics associated with initial velocity vectors projected into the plane containing the radial line through the egocentre and orthogonal to the normal plane. These geodesics resemble those in Figure 5b. (**b**) For ease of comparison with part (a) the family of geodesics in Figure 6b is reproduced here but only half the sphere is plotted.

The simulations show that the family of geodesics $S_{II}$ emanating from any initial point q in the outside world at any Euclidean distance r from the egocentre with unit-length initial velocities $v_{i}$, i=0,1,⋯,35 set in a plane II not normal to the radial line from the egocentre (and not passing through the egocentre) does not remain confined to that initial velocities plane. In Figure 7a the initial point is q=(r(0)=5 m, θ(0)=π2 rad, φ(0)=π4 rad) (i.e., (x = 0, y = 3.54 m, z = 3.54 m), the same as in Figure 6b) but the initial velocities plane II is no longer normal to the radial line. Here it is tilted back π4 radians from the normal plane (i.e., the initial velocities are in the xz-plane passing through the initial point). For ease of comparison, Figure 7b reproduces the spherical geodesics of Figure 6b but only half the sphere is plotted.

The geodesics of Figure 7a follow a weighted combination of both accelerating spiral pathways in the $\partial_{r}\partial_{\theta }$-plane and of constant speed great circle pathways on the sphere passing through the initial point associated with initial velocities in the $\partial_{\theta }\partial_{\varphi }$-plane normal to the radial line. Generalizing this to any initial point and any initial velocity plane tilted at any angle so it is not normal to the radial line from the egocentre and does not pass through the egocentre, the initial velocities can be projected into the $\partial_{r}\partial_{\theta },\;\partial_{r}\partial_{\varphi }$ and $\partial_{\theta }\partial_{\varphi }$ orthogonal coordinate planes at the initial point. Each geodesic with an initial velocity vector in the tilted initial velocities plane can be constructed as a weighted combination of the accelerating spiral and constant speed great circle pathways generated by the initial velocities projected into the coordinate planes. In other words, the families of geodesics associated with initial velocity vectors confined to coordinate planes as illustrated in Figure 5 and Figure 6 are sufficient to characterize all geodesics in (G,g) emanating from any initial point with any initial velocity.

### 5.5. Interpreting Geodesic Simulations

The geodesic families plotted in the outside Euclidean world in Section 5.2, Section 5.3 and Section 5.4 provide a way for us to analyze and understand the warped geometry of the perceived visual manifold attributable to the size–distance relationship introduced by the optics of the eye. As the initial point (r, θ,φ) from which a family of geodesics emanates is moved about in the outside world the spherical coordinate basis vectors $\left(\partial_{r},\partial_{\theta },\partial_{\varphi }\right)$ at the initial point move and rotate with it. In consequence, all the geodesics in Figure 5, Figure 6 and Figure 7 simply rotate with rotation of the radial line between the egocentre and the initial point. In other words, the pattern of geodesics and hence the warping of the perceived visual manifold is isotropic at the egocentre; that is, the same in every direction from the egocentre.

The simulated geodesics illustrated in Figure 5 show that the perception of every plane in the outside world passing through the egocentre is warped in the same way. All the geodesics emanating from any point in such a plane with initial velocities in the plane remain within the plane. The accelerating nature of the geodesics shows that Euclidean distances in the plane are perceived as foreshortened (Figure 3) and the curved nature of the geodesics shows that the plane is perceived as warped. A line connecting any two points in the plane perceived as being a straight line with zero acceleration in the warped perceived visual manifold is actually curved towards the egocentre in the outside world with the actual direction between the two points changing towards the egocentre as the distance between the two points increases. This underlies what is found in the pointing error experiments discussed in Section 8.5.

The simulated geodesics with initial velocities confined to a plane normal to a radial line connecting the egocentre to the initial point form longitude lines on a sphere centred at the egocentre as shown in Figure 6. Such families of simulated geodesics sweep out *visual spheres* defined in Section 3.2 forming spherical surfaces on which the Riemannian metric g is constant. In Section 5.6 below we will see that the visual spheres in the outside world are perceived as planes normal to the direction of gaze in the warped visual manifold (G,g) on which the size of any object is perceived to be constant. Transformation of the visual spheres in the outside world into planes in (G,g) emphasizes the extent of the warping of the perceived visual world introduced by the visual system.

### 5.6. Euclidean Coordinates versus Perceptual Coordinates

**Figure 8:**
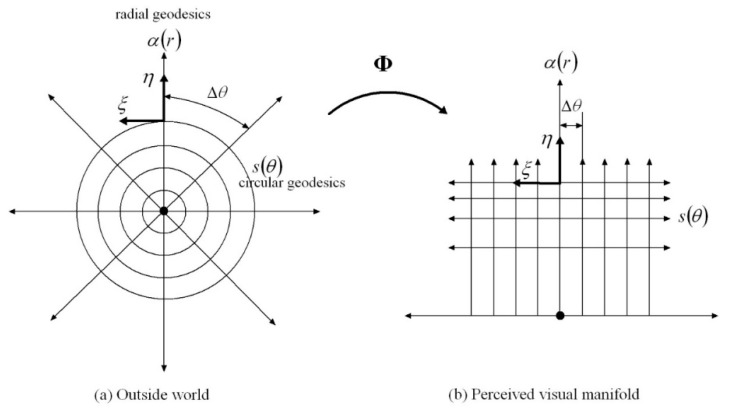
(**a**) A grid of circular and radial geodesics in the outside world in any plane passing through the egocentre represented by the dot • at the origin and (**b**) its image under a conformal map Φ between the plane in the Euclidean outside world and the corresponding plane in the perceived visual manifold with the egocentre again represented by •. The vectors ξ are Killing vectors whose integral flows preserve the metric g. The vectors $\eta =\dot{\alpha }$ are velocity vectors tangent to the radial geodesics α(r). The conformal map Φ maps circular geodesics s(θ) and radial geodesics α(r) intersecting at right angles in the Euclidean outside world to equivalent horizontal straight lines s(θ) and vertical straight lines α(r) intersecting at right angles in the perceived visual manifold. Notice that in (b) the intervals on the horizontal straight lines are equal while the equivalent circular arc-lengths s=rΔθ in the outside world increase linearly. Also, the intervals on the vertical lines in (b) decrease logarithmically while the equivalent radial intervals in (a) are constant. The difference between the two coordinate systems illustrates the profound warping introduced by the visual system.

As shown in Section 5.2, any plane in the outside world that passes through the egocentre is totally geodesic (i.e., any two points in the plane can be joined by a geodesic confined to the plane). We have shown previously [52] that such planes can be spanned by totally geodesic coordinate systems with all of the coordinate grid lines being geodesics. Radial and circular geodesics like those slightly thickened in Figure 5 can be used to build such a geodesic coordinate system. Construction of a totally geodesic coordinate grid for the horizontal plane in the Euclidean outside world using radial and circular geodesics is illustrated in Figure 8a and its conformal transformation into the perceived visual manifold (G,g) is illustrated in Figure 8b.

If the plane in Figure 8a is rotated about any radial line, the circular geodesics sweep out the family of egocentric visual spheres in the outside world seen in Figure 6. The visual spheres are closely related to the *Killing vectors* of (G,g). Killing vector fields ξ (named after the German mathematician Wilhelm Killing 1847–1923) are defined to be those vector fields ξ whose integral flows in the manifold preserve the metric g [109] (Chapter XIII). In the perceived visual manifold (G,g) the metric g(r) in Equation (12) depends only on the Euclidean distance r and, therefore, is constant along points in the manifold corresponding to points along the circular geodesics (on visual spheres) in the outside world. Consequently, all vector fields ξ tangent to points in the manifold on visual spheres in the outside world are g-Killing vector fields and their integral flows in the manifold are isometries; i.e., they preserve metric inner products and metric distances between local points. A Lie group of 2D isometries (transformations) acting on the perceived visual manifold (G,g) will generate the set of visual spheres in the outside world as its 2D orbits. Since the singularity at the egocentre has been removed by the hole described in Section 4.1, the space of orbits (visual spheres) is itself a manifold [110] (Chapter 9).

In Figure 8a the vector field ξ tangent to the circular geodesics is a g-Killing vector field whose integral flow is confined to the circular geodesics and the vector field η normal to the circular geodesics has an integral flow α(r) along the radial geodesics connecting one circular geodesic to the next. These circular and radial geodesics form a totally geodesic coordinate system for the horizontal plane passing through the egocentre in the outside world.

Equations (34) and (35) are derived from the theory of Killing vector fields [109] (Chapter XIII):

(34) $$\mathrm{grad}{\left\|\xi \right\|}_{g}^{2}=-2\nabla_{\xi }\xi =0,$$

(35) $$\nabla_{\eta }{\langle \xi ,\eta \rangle }_{g}=0.$$

Equation (34) implies that for any point on any visual sphere the gradient of the metric norm ‖ξ‖g2=〈ξ,ξ〉g12 of the Killing vector ξ at the corresponding point in the manifold is zero. In other words, the metric norm is unchanged by movement in any direction in the 3D (G,g) manifold. This might seem counterintuitive but it has to be kept in mind that the metric g causes metric distances in (G,g) to shrink in all three dimensions in inverse proportion to Euclidean distance r. Equation (35) implies that the metric inner product ${\langle \xi ,\eta \rangle }_{g}$ remains constant along points in the manifold corresponding to any radial geodesic α(r) in the outside world. Consequently it can be deduced from Equations (34) and (35) that both η and ξ and their metric inner products ${\langle \eta ,\eta \rangle }_{g}$, ${\langle \xi ,\xi \rangle }_{g}$, and ${\langle \eta ,\xi \rangle }_{g}$ are parallel translated and remain constant along pathways in the manifold corresponding to both radial geodesics and great circle geodesics in the outside world. As seen in Figure 8a, the angle between the velocity vector η and the g-Killing vector ξ in the outside world equals π2 radians at every point in the plane. The equivalent straight lines in Figure 8b also intersect at an angle of π2 radians in the perceived visual manifold (G,g) consistent with a conformal mapping between the outside world and (G,g).

Remember that geodesics are accelerating curves in the outside world that are perceived as constant speed straight lines in the perceived visual manifold (G,g). Consequently, points in the manifold corresponding to circular geodesics in Figure 8a are constant speed straight lines in Figure 8b. From the properties of Killing vector fields described above, it follows that the metric speed ‖ξ‖g=〈ξ,ξ〉g(r)12 along any straight line in Figure 8b corresponding to a circular geodesics in Figure 8a is constant and independent of the distance r. Since the length along the straight-line pathway in the manifold (Figure 8b) corresponding to the arc-length s=rΔθ in the outside world (Figure 8a) is computed by integrating the metric speed ${\left\|\xi \right\|}_{g}$ along the straight-line pathway, and since the metric speed ${\left\|\xi \right\|}_{g}$ in the manifold is constant and independent of the distance r, it follows that the perceived interval along the straight-line pathway in the manifold is determined by the angle Δθ and is independent of the distance r. Thus, radial lines evenly spaced by angle Δθ in the outside world (Figure 8a) correspond to parallel vertical straight lines in the manifold (G,g) with a fixed interval between them independent of the perceived distance *r* (Figure 8b). This is consistent with the observation that human observers using monocular viewing treat diverging ‘visual rays’ as experientially parallel [29]. Similarly, circular geodesics evenly spaced by distance Δr in the outside world correspond to horizontal straight lines spaced logarithmically in the perceived visual manifold (G,g) because of the logarithmic relationship between actual distance and perceived distance described in Section 4.1. These vertical and horizontal straight lines in the manifold (G,g) form a geodesic coordinate system spanning the horizontal plane passing through the egocentre in (G,g).

Figure 8 also illustrates three points made earlier. (i) While the angle between the vertical and horizontal straight lines in (G,g) equals π2 radians everywhere, as does the angle between the radial and circular geodesics in the outside world, the lengths of intervals on those lines in (G,g) are stretched or compressed relative to the corresponding interval lengths in the outside world. This is consistent with conformal mapping between the Euclidean outside world and the perceived visual manifold (G,g) (Section 4.1 and Figure 3). (ii) The horizontal straight line passing through the egocentre in Figure 8b corresponds to a point at the egocentre in Figure 8a and, therefore, is infinitely stretched. But this singularity is avoided by virtue of the hole in visual perception about the egocentre (Section 4.1). (iii) Since the warping of (G,g) is the same for every plane passing through the egocentre, including the vertical $\partial_{r}\partial_{\varphi }$-plane, it follows that the perceived size of a 3D object in the environment is determined by the solid angle it subtends at the egocentre or equivalently, the perceived size of an object varies in inverse proportion to the Euclidean cyclopean distance *r* in the outside world (Section 1). Notice that the perceived size of an oblique line decreases as the angle it subtends at the egocentre decreases and eventually shrinks to a dot when it is aligned with the direction of cyclopean gaze. This is different from the judged length of an oblique line that requires a computation described below in Section 6.1.

## 6. Binocular Perception of the Size and Shape of Objects

When seen from a fixed place, the perceived surfaces of objects in the environment are represented geometrically by 2D curved surfaces with boundary (or surfaces with corners) isometrically embedded in the perceived visual manifold (G,g). The space between objects in the environment is transparent and so the perceived image-point vectors for points in the outside world between objects are zero vectors. Similarly, points in the environment occluded from view by other objects also have zero image-point vectors. Image points on embedded 2D surfaces are easily detected, therefore, because they are the only image points with non-zero image-point vectors. Image points on the boundaries (edges) of perceived 2D embedded surfaces are also easily detected because they correspond to points in (G,g) where the image points and/or the image-point vectors change rapidly from a foreground to a background surface. In the case of a semi-transparent object there are two images, one from transmission through the object and the other a reflection from the semi-transparent object. Reflection causes the orientation of the image to reverse and the vector bundle is said to be twisted. Although reflections can be handled within Riemannian geometry we will not explore them further in this paper. In aerial perspective the atmosphere causes images of objects to become hazy with increasing distance. But still the image-point vectors of the hazy object do not become zero. Thus image-point vectors of embedded surfaces, including reflecting surfaces, are easily detected because they are the only points in (G,g) with non-zero image-point vectors.

We will use the notation $\left(\tilde{G},\tilde{g}\right)$ to represent a 2D submanifold (surface) embedded in the 3D ambient perceived visual manifold (G,g), and $\partial \tilde{G}$ to represent its boundary. As described in Riemannian geometry [57] (Chapter 8), an isometric embedding of $\left(\tilde{G},\tilde{g}\right)$ into (G,g) is a smooth map (isometric embedding) ι: $\left(\tilde{G},\tilde{g}\right)\to \left(G,g\right)$ with the unknown metric $\tilde{g}$ at each point on $\left(\tilde{G},\tilde{g}\right)$ induced by the pull back $\tilde{g}=\iota^{*}g$ of the known metric g on the ambient manifold. The smooth map ι: $\left(\tilde{G},\tilde{g}\right)\to \left(G,g\right)$, and hence the metric $\tilde{g}$, depends on the shape of the object in the outside world and this shape is unknown. It follows that the pulled-back metric $\tilde{g}=\iota^{*}g$ and the shape of the embedded surface $\left(\tilde{G},\tilde{g}\right)$ have to be computed from the image-point vectors in the G-memory of Section 3.

The unknown metric $\tilde{g}$ on a 2D embedded submanifold $\left(\tilde{G},\tilde{g}\right)$ is different from the known metric g at the same point in the ambient manifold (G,g), and its rate of change across the surface of the embedded submanifold is different from the rate of change of g along the same path in the ambient manifold (G,g). The 2D embedded submanifold is perceived, therefore, as a 2D surface $\left(\tilde{G},\tilde{g}\right)$ with boundary $\partial \tilde{G}$ that is curved relative to the ambient 3D perceived visual manifold (G,g) in which it is embedded. Computing the size and shape of embedded surfaces from image-point vectors stored in G-memory is complicated by the fact that all perceived measures of the surface are made relative to the ambient perceived visual manifold (G,g) and this ambient manifold is itself curved (warped) relative to the Euclidean outside world.

### 6.1. Seeing the Size of an Object

Consider measuring the size of an object in the outside world with a tape measure. The perceived size of both the object and the tape measure change with distance r in exactly the same way so the size of the object according to the measure given by the tape remains the same regardless of the distance r. Now consider the concept of a “perceptual tape measure”. By this we mean an internal reference metric that changes its infinitesimal length ds as a function of r in exactly the same way as does the perceived image of an actual tape measure. The use of such an internal reference allows the actual size of an object to be determined regardless of its position in the scene. Since the metric g decreases smoothly as the distance r from the egocentre increases, it follows that the infinitesimal length ds at a point along a curve γ(s) in (G,g) changes as the point on the curve moves closer or further away from the egocentre. Despite this differential expansion/contraction of the curve, a measure of its length L between any two points $\gamma \left(s_{i}\right)$ and $\gamma \left(s_{f}\right)$ along the curve is always obtainable by integrating its metric speed ‖γ˙‖g=〈γ˙,γ˙〉g12 along the curve between the points:

(36) L=∫sisf〈γ˙,γ˙〉g12ds.

Given an internal reference metric that can be moved to any point in (G,g), Equation (36) provides an implementation of a perceptual tape measure able to measure the actual size of perceived objects and distances between points in the outside world taking the warped geometry of the perceived visual space into account. In other words, Equation (36) allows the internal reference metric to change its length as a function of distance *r* in (G,g) in exactly the same way the perception of an actual tape measure changes its length as a function of distance *r* in the outside world. As put by Frisby and Stone, “[the object] looks both smaller and of the correct size given its position in the scene” [40] (p. 41). The precision of a measurement made using a perceptual tape measure will decrease as the object being measured moves further away from the egocentre because of the reduced size of both the object and the perceptual tape. Incorrect estimates of Euclidean distance r will alter the differential stretching of the perceived curve and lead to misperceptions of both apparent and actual size as well as to other illusions (Section 2.8 and Section 8.4).

### 6.2. Seeing the Outline of an Object

The perceived shape of the boundary of a two-dimensional submanifold embedded in (G,g) (or at least of those segments of the boundary that belong to the object and not to other occluding objects) plays an important role in object recognition. Sketching the outline of a hand, for example, provides sufficient information to recognize that the object is a hand. This is an interesting observation because both the perceived boundary and the perceived shape of the boundary of an object vary with the position and orientation of the object in the environment relative to the observer and are not invariant properties of the object. Actually, a smooth 3D object in the outside world does not have an edge and the perceived boundary corresponds to a curve γ(s) on the surface of the 3D object (and in the ambient manifold) that varies depending on the position and orientation of the object relative to the observer. Nevertheless, when observed from a fixed place, objects in the environment have clearly perceivable boundaries or edges.

Cutting and Massironi [112] and Cutting [98] pointed out that many cave paintings, as well as cartoons, caricatures and doodles are made of lines and, as images, they depict objects well. They proposed a taxonomy of lines [112], describing *edge lines* that separate a figure from the background, *object lines* where the line stands for an entire object in front of the background, *crack lines* that imply an interior space hidden from view, and *texture lines* that can represent small edges, small objects, small cracks as well as changes in shading and colour. They also pointed out that by using just a few well-crafted lines an artist can sketch the outline of partly occluded objects in such a way that both the occluding and the occluded objects can be recognized.

Despite the variety of line types, when looking at three-dimensional objects in the environment from a fixed place, lines are representations of the boundaries (or segments of boundaries) of perceived two-dimensional submanifolds embedded in the perceived visual manifold (G,g). The perceived edge of the object is a curve (or segment of a curve) γ(s) embedded in the perceived visual manifold (G,g). The perceived shape of the outline of the object is quantified by the perceived curvature $\kappa \left(s_{0}\right)$ at each point $\gamma \left(s_{0}\right)$ along the curve. As given by Lee [57] for curves in Riemannian manifolds in general, the curvature $\kappa \left(s_{0}\right)$ of a unit metric speed curve γ(s) at each point $\gamma \left(s_{0}\right)$ along the curve is equal to the metric acceleration of the unit metric speed curve at that point.

As shown in Section 4.3, the perceived acceleration of a point moving along a unit metric speed curve γ(s) in the ambient perceived visual manifold (G,g) is given by the covariant derivative $\nabla_{\dot{\gamma }}\dot{\gamma }\left(s_{0}\right)$ at each point $\gamma \left(s_{0}\right)$ along the curve. Since the covariant derivative $\nabla_{\dot{\gamma }}\dot{\gamma }$ is zero for a geodesic curve in (G,g) (geodesic curves appear as constant metric speed straight lines), it follows that the perceived curvature $\kappa \left(s_{0}\right)$ provides a quantitative measure of how far the unit metric-speed boundary curve γ(s) deviates from a geodesic in the ambient manifold at each point along the curve. Thus, the perceived shape of the outline of an object is encoded by the perceived covariant derivatives $\nabla_{\dot{\gamma }}\dot{\gamma }\left(s_{0}\right)$ in the ambient manifold at each point $\gamma \left(s_{0}\right)$ along the curve. The apparent curvature is perceived, therefore, relative to the inherent curvature of the ambient manifold at each point.

### 6.3 Seeing the Shape of an Object

As the gaze point Q=(r, θ, φ) is moved about on the surface of an object in the environment, the perceived 2D surface $\left(\tilde{G},\tilde{g}\right)$ is described by a smooth function r=f(θ,φ) between the Euclidean cyclopean distance r and the cyclopean direction (θ,φ). Indeed, providing the point q=(r, θ, φ) on the surface $\left(\tilde{G},\tilde{g}\right)$ is within the functional region of central vision, the function r=f(θ,φ) can be computed for the space about a single point of gaze. The partial derivatives ∂f∂θ and ∂f∂φ of this function at each point $q\in \left(\tilde{G},\tilde{g}\right)$ on the surface define two vectors in the 3D ambient tangent space $T_{q}G$ at each point on the surface. The two vectors span a 2D subspace $T_{q}\tilde{G}$ in $T_{q}G$ that is tangent to the 2D submanifold $\left(\tilde{G},\tilde{g}\right)$ at the point $q\in \left(\tilde{G},\tilde{g}\right)$. Note that each point $q\in \left(\tilde{G},\tilde{g}\right)$ on the submanifold is also a point q∈(G,g) in the ambient manifold. The cross product ∂f∂θ×∂f∂φ=N of these two vectors defines a vector N in $T_{q}G$ at each point q∈(G,g) that is normal to the 2D submanifold $\left(\tilde{G},\tilde{g}\right)$ at each point $q\in \left(\tilde{G},\tilde{g}\right)$. The vector N at each point can be normalized to obtain a unit length vector n=N〈N,N〉g12 that spans the one-dimensional subspace $N_{q}\tilde{G}$ normal to the submanifold $\left(\tilde{G},\tilde{g}\right)$ at each point $q\in \left(\tilde{G},\tilde{g}\right)$. It follows from this that the second fundamental form II(X,Y) (defined in Appendix B) is a vector normal to the surface in the normal bundle $N_{q}\tilde{G}$ at each point $q\in \left(\tilde{G},\tilde{g}\right)$ that can be written as:

(37) II(X,Y)=h(X,Y)n,

where h(X,Y) is a real number (scalar) equal to the metric length $\|II{\left(X,Y\right)\|}_{g}$ of the vector II(X,Y).

Thus (see Appendix B) we can replace the vector-valued second fundamental form II with a simpler scalar-valued form h known as the *scalar second fundamental form*. This acts on any two vectors X and Y in $T_{q}\tilde{G}$ and transforms them into a real number h(X,Y) at every point $q\in \left(\tilde{G},\tilde{g}\right)$. Therefore, it is a symmetrical 2-covariant tensor field over the submanifold $\left(\tilde{G},\tilde{g}\right)$. Using the index raising lemma and the tensor characterization lemma Lee [57] (Chapter 8) shows that h(X,Y) can be expressed in the form:

(38) $$h\left(X,Y\right)={\langle SX,Y\rangle }_{g},$$

where S is a linear, symmetrical, nonsingular, matrix operator, known as the *shape operator*. It is a linear endomorphism that operates on any vector X in $T_{q}\tilde{G}$ and transforms it into another vector SX in $T_{q}\tilde{G}$. Because the shape operator S is a symmetrical matrix it has two orthonormal eigenvectors $E_{1}$ and $E_{2}$ in the tangent space $T_{q}\tilde{G}$ known as the *principal directions* and two corresponding eigenvalues $\kappa_{1}$ and $\kappa_{2}$ known as the *principal curvatures* of the submanifold $\left(\tilde{G},\tilde{g}\right)$ at the point $q\in \left(\tilde{G},\tilde{g}\right)$. In other words:

(39) $$SE_{1}=\kappa_{1}E_{1}\;\mathrm{and}\;SE_{2}=\kappa_{2}E_{2\;}.$$

The principal curvature $\kappa_{1}$ equals the maximum curvature of the submanifold $\left(\tilde{G},\tilde{g}\right)$ at the point $q\in \left(\tilde{G},\tilde{g}\right)$ in the principal direction $E_{1}$. The principal curvature $\kappa_{2}$ equals the minimum curvature of the submanifold $\left(\tilde{G},\tilde{g}\right)$ at the point $q\in \left(\tilde{G},\tilde{g}\right)$ in the principal direction $E_{2}$.

In Appendix B we show that the covariant derivative $\nabla_{E_{1}}n$ provides measures of the perceived principal curvature $\kappa_{1}$ and the principal direction $E_{1}$ of the submanifold at each point $q\in \left(\tilde{G},\tilde{g}\right)$. Likewise the covariant derivative $\nabla_{E_{2}}n$ provides measures of the perceived principal curvature $\kappa_{2}$ and the principal direction $E_{2}$ of the submanifold at each point $q\in \left(\tilde{G},\tilde{g}\right)$. Although the covariant derivative $\nabla_{E_{1}}n$ is computed in the ambient manifold (G,g), the vector $\nabla_{E_{1}}n$ is contained in the 2D vector space $T_{q}\tilde{G}$ tangent to the submanifold at the point $q\in \left(\tilde{G},\tilde{g}\right)$. The principal direction vector $E_{1}$ is easy to find, being the only vector in $T_{q}\tilde{G}$ for which the two vectors $\nabla_{E_{1}}n$ and $E_{1}$ in $T_{q}\tilde{G}$ are collinear. Because the shape operator S is a non-singular symmetrical matrix, eigenvector $E_{2}$ is orthogonal to $E_{1}$ so it too is easy to find. The metric length ${\left\|\nabla_{E_{1}}n\right\|}_{g}$ of the covariant derivative vector $\nabla_{E_{1}}n$ equals the maximum principal curvature $\kappa_{1}$ of the submanifold at the point $q\in \left(\tilde{G},\tilde{g}\right)$ and the metric length ${\left\|\nabla_{E_{2}}n\right\|}_{g}$ of the covariant derivative vector $\nabla_{E_{2}}n$ equals the minimum principal curvature $\kappa_{2}$ of the submanifold at the same point. It makes intuitive sense that the rate at which the normal vector n rotates as the point $q\in \left(\tilde{G},\tilde{g}\right)$ moves across the surface of the submanifold is related to the curvature of the submanifold. The more curved the submanifold, the greater the rate of rotation of the normal vector n.

Equation (A9) in Appendix B shows that the perceived curvature at each point $q\in \left(\tilde{G},\tilde{g}\right)$ on a submanifold $\left(\tilde{G},\tilde{g}\right)$ is equal to the product $\kappa_{1}\kappa_{2}$ of the principal curvatures at that point. However, the product $\kappa_{1}\kappa_{2}$ is equal to:

(40) $$\kappa_{1}\kappa_{2}={\tilde{R}}_{m}\left(E_{1},E_{2},E_{2},E_{1}\right)-R_{m}\left(E_{1},E_{2},E_{2},E_{1}\right).$$

That is, the perceived curvature $\kappa_{1}\kappa_{2}$ equals the difference between the *Gaussian curvature* $\tilde{K}\left(E_{1},E_{2}\right)={\tilde{R}}_{m}\left(E_{1},E_{2},E_{2},E_{1}\right)$ of the submanifold $\left(\tilde{G},\tilde{g}\right)$ at the point $q\in \left(\tilde{G},\tilde{g}\right)$ and the *sectional curvature* $K\left(E_{1},E_{2}\right)=R_{m}\left(E_{1},E_{2},E_{2},E_{1}\right)$ of the ambient manifold (G,g) at the same point q∈(G,g).

The Gaussian curvature $\tilde{K}\left(E_{1},E_{2}\right)={\tilde{R}}_{m}\left(E_{1},E_{2},E_{2},E_{1}\right)$ of the submanifold depends on the unknown metric $\tilde{g}$ induced on the submanifold by the embedding ι: $\left(\tilde{G},\tilde{g}\right)\to \left(G,g\right)$ and consequently, it is influenced by the actual shape of the object in the Euclidean outside world. However, the Gaussian curvature of the submanifold is not an intrinsic property of the object but varies with the position and orientation of the embedded submanifold $\left(\tilde{G},\tilde{g}\right)$ in the ambient perceived visual manifold (G,g). This is contrary to Gauss’s famous *theorema egregium* that asserts that the Gaussian curvature is an intrinsic property of the object, but that theorem only holds for submanifolds embedded in Euclidean spaces. Here we are considering a submanifold embedded in a curved ambient perceived visual space (G,g). As shown in Appendix B, the sectional curvature $K\left(E_{1},E_{2}\right)=R_{m}\left(E_{1},E_{2},E_{2},E_{1}\right)$ of the ambient manifold (G,g) depends on the position q∈(G,g) in the ambient manifold and on the orientation of the plane II in $T_{q}G$ spanned by the orthonormal eigenvectors $E_{1}$ and $E_{2}$ that are tangent to the submanifold at the point $q\in \left(\tilde{G},\tilde{g}\right)$. From this we see that, while the perceived shape of the 2D submanifold embedded in the perceived visual manifold (G,g) is influenced by the intrinsic shape of the object in the environment, it does not equal the intrinsic shape but varies as a function of the position and orientation of the object relative to the egocentre of the observer.

**Figure 9:**
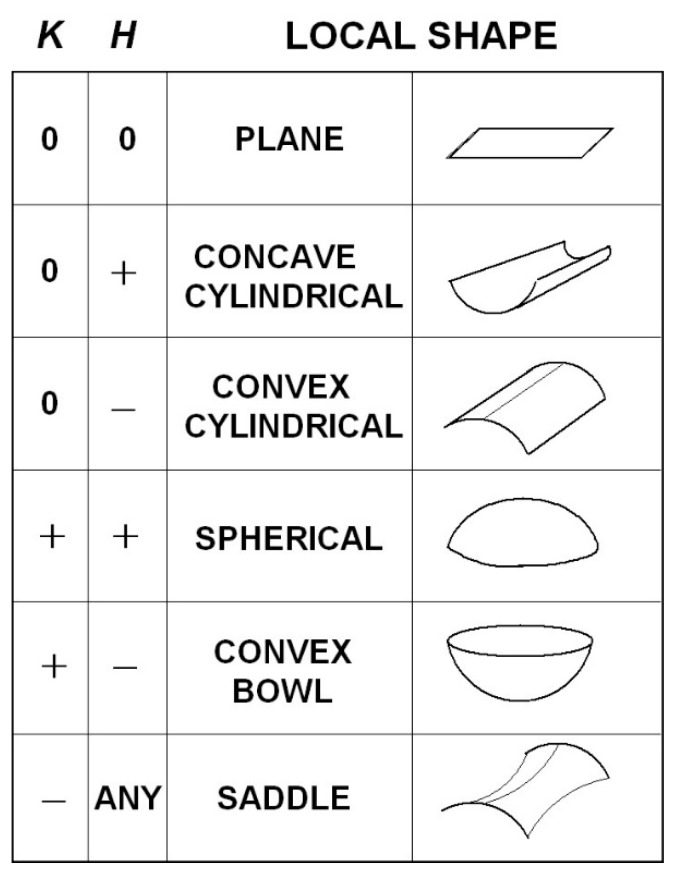
A table illustrating how the combination of the product $K=\kappa_{1}\kappa_{2}$ and the mean H=(κ1+κ2)2 of the principal curvatures $\kappa_{1}$ and $\kappa_{2}$ is sufficient to encode the local shape of the submanifold uniquely at each point on the submanifold [113].

To show that the perceived curvature $\kappa_{1}\kappa_{2}$ at every point on the surface of an object seen from a fixed place is not sufficient to determine uniquely the perceived shape of the surface, consider the example of a cylinder embedded in Euclidean space. The maximum principal curvature $\kappa_{1}$ is measured in the direction $E_{1}$ tangent to the circumference of the cylinder and the minimum principal curvature $\kappa_{2}$ is measured in the direction $E_{2}$ tangent to the long axis of the cylinder. The minimum principal curvature $\kappa_{2}$ is zero everywhere along the long axis of the cylinder so the product $\kappa_{1}\kappa_{2}$ is zero at every point on the cylinder. But the cylinder has a different shape from the Euclidean plane for which the product $\kappa_{1}\kappa_{2}$ also equals zero. Thus, the product $\kappa_{1}\kappa_{2}$ is not sufficient to uniquely encode the local shape of the submanifold. As described by Trucco and Verri [113] and illustrated in Figure 9, it requires a combination of the mean H=(κ1+κ2)2 and the product $K=\kappa_{1}\kappa_{2}$ of the principal curvatures to encode the local shape uniquely.

## 7. A Geometric Representation of Visuospatial Memory

Thus far, we have described G-memory as providing an internal representation of the egocentric 3D outside world viewed stereoscopically from a fixed place in that world. We have proposed that, during each interval of fixed gaze, the encoded left and right image-point vectors associated with each point in the environment are stored in G-memory in association with their cyclopean coordinates (r, θ, φ). Thus, over time, through visual scanning of the environment from the fixed place, the G-memory accumulates an image of the entire 3D environment as seen from that place. The warped geometry of the perceived visual space is encoded by the Riemannian metric g(r, θ, φ) stored at each site (r, θ, φ) in the G-memory. Thus the G-memory encodes both the warped geometry of the perceived environment and the image of the environment as seen from the fixed place.

We now extend the model of G-memory to include moving the head (egocentre) from place to place in the environment. The size–distance relationship introduced by the optics of the eye is unchanged by moving the head from place to place (and hence the metric g(r, θ, φ) is unchanged) but the retinal image *does* change because of changes in perspective associated with the different viewpoints. In this section, we first describe a geometric structure (known as a *vector bundle*) for G-memory associated with a fixed place, and we then extend this to a geometric structure (known as a *fibre bundle*) to account for variable place. The extended geometric structure incorporates a partitioning of visuospatial memory into a family of sub-memories (or G-memories) accessed by the hippocampal encoding of place. Thus the different sub-memories encode images of the environment seen from different places (i.e., place-encoded images). Finally, we describe a geometric mapping between sub-memories (known as a *vector-bundle morphism*) that can remove occlusions and turn visuospatial memory into a 3D cognitive model of the visual environment as seen from different places in that environment.

### 7.1. The Geometric Structure of G-Memory for a Fixed Place

**Figure 10:**
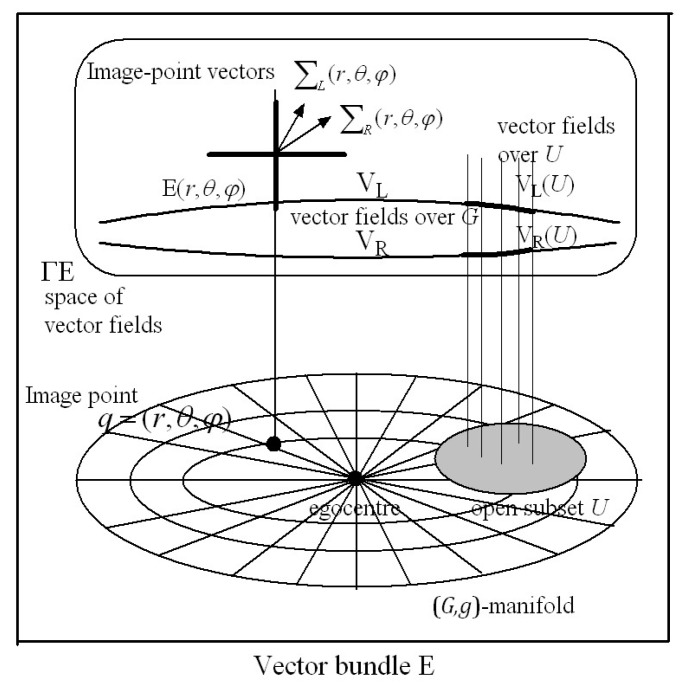
A schematic diagram illustrating the geometric vector bundle structure of G-memory for a fixed place. The cyclopean coordinates q=(r, θ, φ) of each image point in (G,g) act as a memory accession code for the storage and retrieval of the image-point vectors $\Sigma_{L}\left(r,\;\theta ,\;\varphi \right)\;$ and $\Sigma_{R}\left(r,\;\theta ,\;\varphi \right)$. The union of all the image-point vectors $\Sigma_{L}$ and $\Sigma_{R}$ forms two 30-dimensional vector fields **V**_L_ and **V**_R_ in the space of vector fields ΓE over (G,g). U represents an open subset in (G,g) with $V_{L}\left(U\right)$ and $V_{R}\left(U\right)$ the vector fields over the open subset U.

As described in Section 3, each site q=(r, θ, φ) in G-memory corresponds to a point in the 3D Euclidean outside world specified in cyclopean spherical coordinates relative to the egocentric place of the head in the environment (i.e., the egocentre). The G-memory can, therefore, be taken to be an internal representation of 3D egocentric perceived visual space. We now consider the geometric structure of that space. When each point q=(r, θ, φ) in the perceived visual manifold (G,g) is associated with image-point vectors $\Sigma_{L}\left(r,\;\theta ,\;\varphi \right)$ and $\Sigma_{R}\left(r,\;\theta ,\;\varphi \right)$ stored at that site, the structure takes on a geometric form illustrated in Figure 10.

At each image point q=(r, θ, φ) in the manifold (G,g) there exists a fibre (i.e., a *geometric* fibre not a nerve fibre) containing a 30-dimensional vector space E(r, θ, φ) in which two 30-dimensional image-point vectors $\Sigma_{L}\left(r,\;\theta ,\;\varphi \right)$ and $\Sigma_{R}\left(r,\;\theta ,\;\varphi \right)$ are stored. The fibre includes both the image point q=(r, θ, φ) and the vector space E(r, θ, φ) so the union of all the fibres over all points q=(r, θ, φ) in (G,g) equals the total space E called a *vector bundle* π:E→G.

A vector bundle is a well-known structure in Riemannian geometry. It consists of a pair of smooth spaces, E (the total space) and G (the base space), with a smooth surjective (onto) map π:E→G (the projection) between them [57] (Chapter 2). The total space E is a smooth 33-dimensional manifold; three dimensions are required to specify the position q=(r, θ, φ) in (G,g) and 30 dimensions are required to specify each image-point vector $\Sigma_{L}\left(r,\;\theta ,\;\varphi \right)$ and $\Sigma_{R}\left(r,\;\theta ,\;\varphi \right)$ in the vector space E(r, θ, φ) at q=(r, θ, φ) in (G,g). A *section* of E is a map V:E→G such that each V(r, θ, φ)∈E(r, θ, φ) is an image-point vector ($\Sigma_{L}\left(r,\;\theta ,\;\varphi \right)$ or $\Sigma_{R}\left(r,\;\theta ,\;\varphi \right)$) over its image point q=(r, θ, φ). Each section V:E→G is thus a vector field made up of all the image-point vectors over all the points q=(r, θ, φ) in (G,g) acquired through visual scanning with the head in a fixed place. The vector space of all possible vector fields V over (G,g)) is depicted by the notation ΓE (Figure 10).

The vector fields **V**_L_ and **V**_R_ in ΓE (consisting of all the left and right image-point vectors $\Sigma_{L}\left(r,\;\theta ,\;\varphi \right)$ and $\Sigma_{R}\left(r,\;\theta ,\;\varphi \right)$ over all the image-points q=(r, θ, φ) in (G,g) accumulated within a single vector bundle π:E→G through visual scanning) thus encode a visual image of the entire 3D environment as seen from the given fixed place. However, while we describe vector fields **V**_L_ and **V**_R_ as being defined over all image points q=(r, θ, φ) in (G,g), it must be kept in mind from Section 6 that the image-point vectors $\Sigma_{L}\left(r,\;\theta ,\;\varphi \right)$ and $\Sigma_{R}\left(r,\;\theta ,\;\varphi \right)$ are only non-zero at those points q that are located on the surfaces of objects. We also note again here that if the visual environment includes reflections then the orientation of the reflected images is reversed and the vector bundle is said to be twisted [114].

An important geometric idea illustrated in Figure 10 is the notion of open subsets U in (G,g). An open subset U can be expanded or contracted to any size. It can even cover all of (G,g). Vector fields **V**_L_(U) and **V**_R_(U) confined to open subsets U in (G,g) can be defined and all the fibres within these can be parallel processed as a unit. Indeed, it is this point processing (i.e., within fibre) nature of computations in Riemannian geometry that makes this geometry so well suited for describing parallel processing in the nervous system.

If images from a sufficiently large number of gaze points with the head in a fixed place are accumulated in the vector bundle π:E→G then, because of the rule for overwriting image-point vectors (Section 3.1), the two vector fields **V**_L_ and **V**_R_ in ΓE can be regarded as fused into a single binocular vector field **V** over (G,g). For simplicity of description, in subsequent sections we assume that a sufficient number of gaze points have been accumulated through visual scanning for the vector fields **V**_L_ and **V**_R_ in ΓE to be fused into a single binocular vector field **V** over (G,g) in the vector bundle π:E→G.

### 7.2. The Geometric Structure of Visuospatial Memory with Place Encoding

As a person moves from place to place in a local Euclidean environment the images of objects projected on to the retinas change according to changes in the perspective from which they are viewed. We propose that visuospatial memory is partitioned into sub-memories, each represented geometrically by a vector bundle $\pi_{p}:E_{p}\to \left(G_{p},g\right)$ with p being an accession code corresponding to the place p ∈ P of the head (egocentre) in the 3D Euclidean outside world. As indicated in Section 1, it is well established that a place map P is neurally encoded in the hippocampus [43,44,45,46,47,48,49,50] and we see this as providing the place manifold P in what follows. Every time the person passes through a given place p ∈ P in the environment, the retinal images, acquired from a sequence of fixed gaze points with the head in that place, are encoded and stored into the appropriate vector bundle sub-memory $\pi_{p}:E_{p}\to \left(G_{p},g\right)$ associated with that place. The stored images are continuously updated through experience. Over time, many images of the environment acquired by visual scanning from different places in the environment are accumulated into the appropriate place-related vector bundle sub-memories. Since each vector bundle $\pi_{p}:E_{p}\to \left(G_{p},g\right)$ is associated with a different place p ∈ P, we refer to the images of the environment stored in different vector bundle sub-memories as *place-encoded* visual images. The street-view feature of Google maps provides a useful analogy. The street address on the Google map is analogous to the place p of the head in the environment, while all the street-view images at that point on the Google map are analogous to all the images acquired by visual scanning at place p in the environment and stored in the vector bundle $\pi_{p}:E_{p}\to \left(G_{p},g\right)$ associated with p ∈ P.

**Figure 11:**
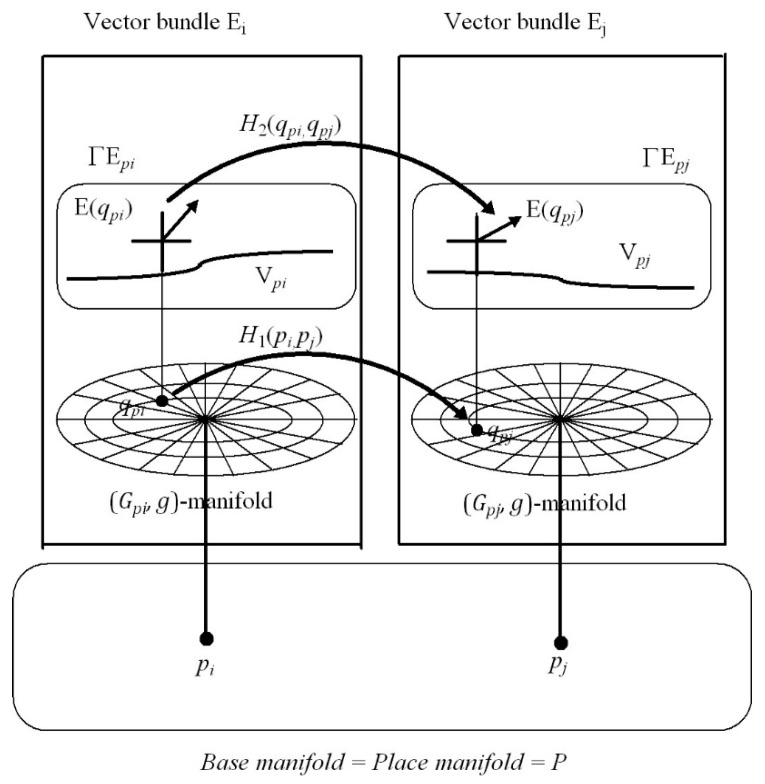
A schematic diagram illustrating the geometric structure of a fibre bundle. The base manifold *P* encodes the place of the head in the Euclidean world. At each place $p_{i}\;\in \;P$ there exists a fibre containing a vector bundle. The vector fields $V_{p_{i}}$ and $V_{p_{j}}$ over the perceived visual manifolds $\left(G_{p_{i}},g\right)$ and $\left(G_{p_{j}},g\right)$ represent the encoded images of the environment seen from places $p_{i}$ and $p_{j}$ respectively. The map $H=\left[H_{1},H_{2}\right]$ between the two vector bundles illustrates a vector-bundle morphism. $H_{1}\left(p_{i},p_{j}\right)$ depends only on the places $p_{i}$ and $p_{j}$ in the place map P while $H_{2}\left(q_{p_{i}},q_{p_{j}}\right)$ depends only on the positions of the image points $q_{p_{i}}$ and $q_{p_{j}}$ in the manifolds $\left(G_{p_{i}},g\right)$ and $\left(G_{p_{j}},g\right).$

Each vector bundle $\pi_{p}:E_{p}\to \left(G_{p},g\right)$ corresponds to a G-memory representing the perceived visual manifold $\left(G_{p},g\right)$ associated with the place p ∈ P. Figure 11 depicts this schematically for two places $p_{i}$ and $p_{j}$. In each vector bundle sub-memory $\pi_{p}:E_{p}\to \left(G_{p},g\right)$ the Riemannian metric g(r, θ, φ) and the warped geometry of the perceived visual manifold $\left(G_{p},g\right)$ are identical. In other words, image points q=(r, θ, φ) in any one sub-memory can be mapped to corresponding image points q=(r, θ, φ) in all other sub-memories and, because the warped geometries of all the $\left(G_{p},g\right)$ manifolds are identical, the flow of image points associated with moving the head from place to place (i.e., *optical flow*) can be represented by the flow of image points in a single equivalent (G,g) manifold.

As shown in Figure 11, the place map P in the hippocampus is represented geometrically by a 3D base Euclidean manifold with a Cartesian external reference frame (X,Y,Z). As the person moves about in the outside world, the movement is represented by a curve p(t), parameterized by time t, in the place manifold P. Since it is not possible to walk through a brick wall or to float up into the air, for example, it is not possible for the point p to move everywhere in the place manifold P. The existence of *no-go places* forms a boundary ∂P on the place manifold P. At each reachable point p ∈ P in the 3D place manifold P, there exists a fibre containing a vector bundle $\pi_{p}:E_{p}\to \left(G_{p},g\right)$ (i.e., a partition of visuospatial memory). Thus, each place p ∈ P acts as an accession code for a vector bundle sub-memory $\pi_{p}:E_{p}\to \left(G_{p},g\right)$ and each vector bundle sub-memory contains a place-encoded visual image of the environment associated with that place.

While the manifolds $\left(G_{p},g\right)$ in the various vector bundles are geometrically equivalent with the same Riemannian metric field g and hence the same warping, the image-point vectors and the vector fields $V_{p}$ stored in each $G_{p}$-memory are different because, although they are images of the same environment, they have each been viewed from a different place p ∈ P in that environment. The changing perspective causes the embedded surfaces of objects in the environment to be stored at different sites in the various $G_{p}$-memories and the embedded surfaces have different sizes, angles, and orientations depending on the place from where they were viewed. Image points occluded from view in one vector bundle are not occluded in others and vice versa. As we will see, it is the difference in the vector fields $V_{p}$ stored in the various $G_{p}$-memories and the way they change from one vector bundle to the next that encodes information about the 3D structure of the outside world.

### 7.3. Fibre Bundles and Vector-Bundle Morphisms

Partitioning of visuospatial memory into vector-bundle sub-memories containing place-encoded visual images can be represented geometrically by a structure in Riemannian geometry known as a *fibre bundle*, illustrated for two places $p_{i}$ and $p_{j}$ in the place manifold P in Figure 11. Every point p ∈ P in the place manifold, together with its associated vector bundle $\pi_{p}:E_{p}\to \left(G_{p},g\right)$, forms a fibre and the union of these fibres forms a fibre bundle. Notice that the definition of a fibre used here is different from that in Section 7.1. Whereas a fibre in Section 7.1 consisted of an image point q=(r, θ, φ) in (G,g) and the associated vector space E(r, θ, φ) in the vector bundle π:E→G, the fibre defined here consists of a point p ∈ P in the place manifold P and an entire vector bundle $\pi_{p}:E_{p}\to \left(G_{p},g\right)$, not just a vector space. This difference in the definition of the fibre defines the difference between the vector bundle and the fibre bundle.

The fibre bundle described does not provide a full 3D representation of the outside world. The vector field $V_{p}$ stored in each vector bundle $\pi_{p}:E_{p}\to \left(G_{p},g\right)$ encodes only the images of curved 2D embedded submanifolds (with boundaries) corresponding to visible patches on the surfaces of objects that can be seen from the fixed place. Theory exists within Riemannian geometry, however, describing maps between vector bundles known as *vector-bundle morphisms* [109,114]. Vector-bundle morphisms allow the place-encoded images stored in the various vector bundles $\pi_{p}:E_{p}\to \left(G_{p},g\right)$ to be mapped reciprocally on to each other. Image points $q_{p_{i}}$ and their associated image-point vectors $\Sigma_{p_{i}}\left(q_{p_{i}}\right)$ in one vector bundle $\pi_{p_{i}}:E_{p_{i}}\to \left(G_{p_{i}},g\right)$ at one point $p_{i}$ in the base manifold P can be transformed by a vector-bundle morphism into the corresponding image points $q_{p_{j}}$ and their associated image-point vectors $\Sigma_{p_{j}}\left(q_{p_{j}}\right)$ in another vector bundle $\pi_{p_{j}}:E_{p_{j}}\to \left(G_{p_{j}},g\right)$ located at another point $p_{j}$ in the base manifold P. Remember, images stored in different vector bundles are of the same environment but seen from different places in that environment and so are different because of differences in perspective. Vector-bundle morphisms can transform the image from any one vector bundle into every other vector bundle. In this way, vector-bundle morphisms can remove occlusions and generate 3D place-encoded images of the 3D outside world with the correct perspective for each place.

### 7.4. Removing Occlusions

To give a simplified illustration of how vector-bundle morphisms can remove occlusions (and generate 3D images), consider two photographs *A* and *B* of the same scene taken from different places in the environment. While it is quickly recognized that the photographs are of the same scene, closer scrutiny will reveal many differences between them. The same image points in the outside world are located at different places in the two photographs. Objects in the photographs may differ in size, the angles between them will be different and the outlines may vary in shape. While many image points in the scene are represented in both photographs, there are also many image points that can be seen in one photograph but are occluded from view in the other. There are also image points occluded from view in both photographs.

Let us define image points that can be seen in both photographs as (++) image points; image points that can be seen in photograph *A* but not in photograph *B* as (+−) image points; image points that can be seen in photograph *B* but not in photograph *A* as (−+) image points; and image points that cannot be seen in either photograph as (−−) image points. In this simplified illustration, a vector-bundle morphism can be thought of as a map $F_{AB}$ able to transform image points (i.e., a small matrix array of pixels depicting a small region of the scene, like the tip of a person’s nose) from photograph *A* to photograph *B*, and an inverse map $F_{BA}$ able to transform image points in the reverse direction from photograph *B* to photograph *A*.

The map $F_{AB}$ can be applied to (++) and (+−) image points in photograph *A*. It transforms (++) image points into image points that can already be seen in photograph *B*. These image points can be used to confirm the precision of the map $F_{AB}$ and errors can be used adaptively to tune the map. The map $F_{AB}$ also transforms (+−) image points in photograph *A* into image points in photograph *B* that are occluded from view in photograph *B*. In other words, the map $F_{AB}$ *fills in* certain image points occluded from view in photograph *B*. Actually, this requires a third-dimension in photograph *B* but we can ignore this in our simplified illustration because, unlike a photograph, the perceived visual manifold $\left(G_{p},g\right)$ in each vector bundle *is* three-dimensional.

A similar argument applies to the map $F_{BA}$ able to transform (++) and (−+) image points in photograph *B* into their corresponding image points in photograph *A,* thereby filling in occluded image points in photograph *A*. Of course, the maps $F_{AB}$ and $F_{BA}$ can do nothing about removing occlusions of (−−) image points. Nevertheless, if a sufficiently large number of photographs of the same scene are taken from a sufficiently large number of different places, and maps F exist between each and every one of these photographs, it is then possible to *fill in* all occlusions in all photographs.

### 7.5. A Geometric Description of Vector-Bundle Morphisms

The maps $F_{AB}$ and $F_{BA}$ in the above simplified illustration play the role of vector-bundle morphisms in the fibre bundle. As illustrated in Figure 11, a vector-bundle morphism $H=\left[H_{1},H_{2}\right]$ between two vector bundles $\pi_{p_{i}}:{\;E}_{p_{i}}\to \left(G_{p_{i}},g\right)$ and $\pi_{p_{j}}:{\;E}_{p_{j}}\to \left(G_{p_{j}},g\right)$ has two parts $H_{1}\left(p_{i},p_{j}\right)$ and $H_{2}\left(q_{p_{i}},q_{p_{j}}\right)$. The first part $H_{1}\left(p_{i},p_{j}\right)$ maps the position of each image point $q_{p_{i}}$ in $\left(G_{p_{i}},g\right)$ to its corresponding position $q_{p_{j}}$ in $\left(G_{p_{j}},g\right)$. The second part $H_{2}\left(q_{p_{i}},q_{p_{j}}\right)$ maps the image-point vector $\Sigma_{p_{i}}\left(q_{p_{i}}\right)$ in the 30-dimensional vector space $E_{q_{p_{i}}}$over $q_{p_{i}}\in \left(G_{p_{i}},g\right)$ in the vector bundle $\pi_{p_{i}}:{\;E}_{p_{i}}\to \left(G_{p_{i}},g\right)$ to the corresponding image-point vector $\Sigma_{p_{j}}\left(q_{p_{j}}\right)$ in the 30-dimensional vector space $E_{q_{p_{j}}}$over $q_{p_{j}}\in \left(G_{p_{j}},g\right)$ in the vector bundle $\pi_{p_{j}}:{\;E}_{p_{j}}\to \left(G_{p_{j}},g\right)$.

The first part $H_{1}\left(p_{i},p_{j}\right)$ is easy to model because the change in position Δq of each image point in the equivalent perceived visual manifold (G,g) is simply equal to the negative of the change $\Delta p=\left(p_{j}-p_{i}\right)$ in the place of the head. Movement of the head in the environment causes all image points q∈(G,g) in the equivalent perceived visual manifold (G,g) to be translated simply by an equal but opposite amount to the change of place $\Delta p=\left(p_{j}-p_{i}\right)$ in the Euclidean place map P, keeping in mind that sites in each $G_{p}$-memory are associated with Euclidean cyclopean coordinates q=(r, θ, φ). Thus:

(41) $$q_{p_{j}}=H_{1}\left(p_{i},p_{j}\right)q_{p_{i}}=q_{p_{i}}-\left(p_{j}-p_{i}\right),$$

where the transformation $H_{1}\left(p_{i},p_{j}\right)$ depends only on the initial and final places $p_{i}$ and $p_{j}$ of the head in the environment. Euclidean coordinates q=(r, θ, φ) can be mapped into (G,g) using the one-to-one, onto, invertible, conformal map described in Section 4.1 and illustrated in Figure 8.

The second part $H_{2}\left(q_{p_{i}},q_{p_{j}}\right)$ of the vector-bundle morphism describes the transformation of the image-point vector $\Sigma_{p_{i}}\left(q_{p_{i}}\right)$ stored at site $q_{p_{i}}\in \left(G_{p_{i}},g\right)$ in the vector bundle $\pi_{p_{i}}:{\;E}_{p_{i}}\to \left(G_{p_{i}},g\right)$ to the corresponding image-point vector $\Sigma_{p_{j}}\left(q_{p_{j}}\right)$ stored at site $q_{p_{j}}\in \left(G_{p_{j}},g\right)$ in the vector bundle $\pi_{p_{j}}:{\;E}_{p_{j}}\to \left(G_{p_{j}},g\right)$. This transformation depends only on the geometry of the perceived visual space (G,g) and, therefore, on the positions of the image points $q_{p_{i}}\in \left(G_{p_{i}},g\right)$ and $q_{p_{j}}\in \left(G_{p_{j}},g\right)$ in the equivalent perceived visual manifold (G,g). It is independent of the place p ∈ P and of the image-point vector $\Sigma_{p_{i}}\left(q_{p_{i}}\right)$ being transformed.

The way image-point vectors are transformed as the head changes place in the environment is independent of the particular visual environment. The laws of optical flow depend only on the changing position of the image point q∈(G,g) in the equivalent perceived visual manifold (G,g). The linear transformation between two 30-dimensional vector spaces containing the image-point vectors $\Sigma_{p_{i}}\left(q_{p_{i}}\right)$ and $\Sigma_{p_{j}}\left(q_{p_{j}}\right)$ associated with a change in the position of the head in the environment depends only on the positions $q_{p_{i}}\in \left(G_{p_{i}},g\right)$ and $q_{p_{j}}\in \left(G_{p_{j}},g\right)$ and is independent of the image-point vectors themselves. The transformation of the image-point vector is described, therefore, by the equation:

(42) $$\Sigma_{p_{j}}\left(q_{p_{j}}\right)=H_{2}\left(q_{p_{i}},q_{p_{j}}\right)\Sigma_{p_{i}}\left(q_{p_{i}}\right),$$

where the linear transformation $H_{2}\left(q_{p_{i}},q_{p_{j}}\right)$ depends only on the sites (positions or accession codes) $q_{p_{i}}$ and $q_{p_{j}}$ of the image points in the $G_{p}$-memories.

Since the metric g is the same for all $G_{p}$-memories and is constant on each visual sphere in each perceived visual manifold $\left(G_{p},g\right)$, it follows that image-point vectors $\Sigma_{p_{i}}\left(q_{p_{i}}\right)$ and $\Sigma_{p_{j}}\left(q_{p_{j}}\right)$ are invariant under transformations between image point positions $q_{p_{i}}\in \left(G_{p_{i}},g\right)$ and $q_{p_{j}}\in \left(G_{p_{j}},g\right)$ confined to the same visual sphere, that is, along the integral flow s(θ) of any g-Killing vector field ξ (Section 5.5). Only a change $r_{i}$ to $r_{j}$ from one visual sphere to another will cause image-point vectors to change. Thus, by adaptively modelling the way foveal image-point vectors change as an object in the environment approaches or recedes along any geodesic integral flow α(r) of a radial vector field $\dot{\alpha }$, the map $H_{2}\left(r_{i},r_{j}\right)$ can be modelled and wired-in to the visual system. Because of the isotropic nature of the geometry of $\left(G_{p},g\right)$ about the egocentre, the particular radial geodesic α(r) is not important. Notice that this is a generalization of the adaptive modelling proposed in Section 3.2 for modelling the relationship between Euclidean distance r of an object and the size of its image on the fovea. In other words, we propose that the nervous system models not only the change in size of the image on the fovea of an object in the environment associated with a change in its Euclidean distance from $r_{i}$ to $r_{j}$ but also models the associated changes in the foveal-hyperfield image features (i.e., image-point vectors) extracted by hypercolumns in V1. This is consistent with binocular gaze trajectories being partitioned into *isovergence arcs* and *isoversion lines* as described by Handzel and Flash [115] in their analysis of the geometry of eye rotations. Isovergence arcs correspond to shifts in gaze confined to visual spheres (i.e., changes in the direction of gaze (θ, φ)) while isoversion lines correspond to shifts in gaze along radial geodesics (i.e., changes in the depth of gaze r). Only changes in foveal images associated with isoversions need be modelled.

## 8. Discussion

The Riemannian geometry of visual space derived and simulated in this paper is essentially the invariant physically-determined geometry attributable to the size–distance relationship introduced by the optics of the human eye. To cope with this imposed warping we contend that the visual system has evolved such that the size–distance relationship given by the 2D images projected on to the foveas is neurally modelled to produce a 3D perceived visual space that matches as closely as possible the Euclidean structure of the 3D world that is actually out there. We also contend that the resulting perceived visual space has an invariant 3D warped geometry that necessarily underlies all other accounts of visual space.

Achieving a 3D correspondence between the perceived and outside worlds requires a means of establishing both the sizes of objects that occur in the retinal images as well as the distances of those objects from the eyes and the egocentre. With both these measures available (Section 2), we argue that the nervous system models adaptively the relationship between them just as it models adaptively sensory–sensory relationships in general, as well as motor–motor, and sensory–motor relationships using non-linear neural adaptive filters [83]. By extending the modelled size–distance relationship for 2D images on the fovea to three dimensions, the perceived 3D size–distance relationship can be determined for each point q=(r, θ, φ) in the environment in the form of a Riemannian metric g(*r*, θ, φ) at each site q=(r, θ, φ) in G-memory (Section 3). This provides the Riemannian 3D visual manifold (G,g). The warped geometry of this manifold has been quantified (Section 4) and simulated (Section 5). In Section 6, the geometry has been applied to perceptions of shape and size while in Section 7 the principles have been extended to give a neurally-feasible account of visuospatial memory and its role in 3D perception.

We now call on the theorems and propositions of Riemannian geometry to discuss how the warping of (G,g) successfully predicts a variety of visual perception phenomena. We also address the relationship of this fundamental invariant geometry to a range of geometries set out by others as well as touching on philosophical issues concerning perception and neural representation in general.

### 8.1. Size Perception

The set of constant tangential speed concentric-circle geodesics in any plane passing through the egocentre in the outside world (Figure 8) corresponds to a cross-section of the visual spheres. If the plane is rotated about a radial line, the concentric circles sweep out concentric visual spheres. The metric g(*r*) is constant on each visual sphere. The metric g(*r*) decreases with increasing r by just the right amount for the perceived arc-length along any circular geodesic between any two radial lines to remain constant regardless of the radius r of the circular geodesic. In other words, the warped geometry of (G,g) causes the perceived arc-length to be determined by the angle Δθ between the two radial lines independently of the distance r and, consequently, the theory predicts that the perceived size of objects in the outside world is determined by the angle they subtend at the egocentre independent of Euclidean distance.

The metric given in Equations (7) and (12) is consistent with the perceived size of a 3D object in the Euclidean outside world varying in all three dimensions in inverse proportion to the Euclidean distance between the egocentre and the object. The perceived size has to vary with Euclidean distance r equally in all three dimensions if objects are to appear to shrink in size as they recede without changing their infinitesimal shape. This is consistent with Hatfield [11] (p. 355), who argued “(1) that visual space exhibits contraction in all three dimensions with increasing distance from the observer, (2) that experienced features of this contraction are […] not the same as would be the experience of a perspective projection onto a frontoparallel plane, and (3) that such contraction is consistent with size constancy”.

A classic example of size diminishing with distance is given by the convergence of railway tracks. Hatfield [39] describes a structure of visual space that is compressed in a Euclidean 3D to 3D projection that allows for railway tracks to converge as they recede in depth while still appearing straight. While Hatfield’s model is not the same as a perspective projection on to a frontoparallel plane, Erkelens [116] shows that such a 3D Euclidean to 2D perspective projection also preserves straight lines. Thus, railway tracks appear to converge but still appear straight in both the Hatfield theory and the Erkelens theory. The perception of straight lines in Euclidean space as straight lines is not inconsistent with the curved visual manifold (G,g) proposed in the Riemannian geometry theory. Geodesics in Figure 5, Figure 6 and Figure 7 are curved pathways in Euclidean space that are perceived as constant speed straight lines in (G,g). But the straight lines in both the Hatfield and Erkelens theories are perceived to be accelerating straight lines, not constant speed straight lines. In other words, a straight line in Euclidean space with intervals of constant length along the line is perceived to be a straight line but with the constant-length intervals perceived as increasing or decreasing smoothly along the line. A point moving at constant speed along the straight line in Euclidean space would be perceived as accelerating or decelerating along a straight line in visual space. Such an accelerating straight line can exist in the curved visual space (G,g) as illustrated in Section 4.2 by the apparent movement of an object off to one side seen from the front window of a train moving along a straight line at constant speed. The object appears to accelerate as it approaches the viewer along a straight line. Such an accelerating straight-line pathway is not a geodesic. A similar phenomenon occurs in the work of those concerned with the perception of slanting planar surfaces; for example, Erkelens [117]. The planar surfaces are perceived as accelerating planar surfaces. A planar surface in Euclidean space, such as a brick wall for example, is perceived as a slanting planar surface, but the apparent size of the bricks changes smoothly with perceived distance along the wall. Again, this is not inconsistent with the curved visual space (G,g) proposed in this paper.

### 8.2. Shape Perception

In Section 6 we showed that warping of the perceived visual manifold affects the perceived shape of objects in the environment. For example, when looking at a point on the surface of an object in the outside world normal to the line of gaze, geodesics originating in that plane do not remain within the plane but form constant tangential speed great circles on a visual sphere, as shown in Figure 6. The surface is perceived as being locally negatively curved, consistent with looking at the inside of a visual sphere (Section 4). When looking at a point on a convex surface (e.g., a positively curved surface in the environment such as the trunk of a tree), it appears slightly flattened because of the negative curvature of the perceived visual manifold. This is consistent with perceived distances being foreshortened relative to their Euclidean distances (Figure 3). This flattening effect decreases rapidly as the convex object moves further away. This is consistent with the observation by Gilinsky [3] (p. 462) that the perceived distance d increases with the true distance D but at a reduced and diminishing rate.

The boundary of an embedded surface $\left(\tilde{G},\tilde{g}\right)$ is perceived as a curve γ(s) parameterized by arc-length s in the ambient manifold (G,g). The covariant derivative $\nabla_{\dot{\gamma }}\dot{\gamma }\left(s_{0}\right)$ in the ambient manifold at each point $\gamma \left(s_{0}\right)$ along the curve provides a measure of the perceived curvature of the boundary. But the perceived curvature $\nabla_{\dot{\gamma }}\dot{\gamma }$ is measured relative to the zero perceived curvature of a geodesic in the ambient manifold passing through the same point. As shown in Section 5, geodesics of the ambient manifold are curved relative to the flat Euclidean outside world and, consequently, the perceived shape of the boundary of an object varies with position and orientation of the object in the outside world relative to the observer.

Similarly, the second fundamental form II(v,v), where v is a unit vector tangent to an embedded surface, provides a perceivable measure of the curvature of the embedded surface in the direction v. However this perceived curvature is measured relative to the zero perceived curvature of a geodesic in the ambient manifold (G,g) passing through the same point in the direction v, so again the perceived shape of the surface varies with the position and orientation of the object in the outside world relative to the observer. The product $K=\kappa_{1}\kappa_{2}$ and the mean H=(κ1+κ2)2 of the principal curvatures provide a measure of the shape of the surface at each point but, as shown in Section 6, the perceived shape $\kappa_{1}\kappa_{2}$ of the embedded surface equals the difference between the *Gaussian curvature* of the submanifold and the *sectional curvature* of the ambient manifold at each point and, consequently, depends on the position and orientation of the surface in the environment relative to the observer.

### 8.3. Warped Geometry

Every plane passing through the egocentre (i.e., any plane rotated about any radial gaze vector (r, θ, φ)) is perceived to be negatively curved but the negative Riemann curvature −1r4 (Section 4.5) decreases rapidly with increasing distance *r* along the radial line. Geodesics other than the radial and circular geodesics in Figure 8 form either outward accelerating or inward decelerating logarithmic spirals (Figure 5). The fact that local spiral geodesics emanating from any point in any plane through the egocentre diverge from each other is consistent with the computation in Section 4.5 showing that sectional curvature is negative everywhere in each plane. This is consistent with Luneburg’s original claim that the perceived visual space is a negatively curved Riemannian space [2]. However, Luneburg argued that it has constant negative curvature, whereas we find the negative curvature decreases rapidly with increasing Euclidean distance r.

While the geometry of the perceived visual manifold is profoundly warped relative to the Euclidean geometry of the outside world, especially at distances close to the egocentre where infinite stretching occurs (Figure 8), the fact that (G,g) is everywhere negatively curved implies that it behaves geometrically in a manner compatible with the behaviour of ordinary Euclidean space [109]. That is, the predicted warping does not induce perceptions of impossible structures inconsistent with the Euclidean geometry of the outside world. For example, the predicted warped geometry is the same in all radial directions from the egocentre just as in Euclidean geometry although the perceived distance is logarithmically foreshortened equally in every direction. Any two points in a negatively-curved space can be connected by a unique geodesic perceived as a constant-speed straight line and the perceived length of the geodesic equals the minimum distance between the two points just as for straight lines in Euclidean space. Two distinct geodesics in (G,g) intersect at only one point and a geodesic defining the shortest distance between a point q and any arbitrary curve in (G,g) always intersects the curve at right angles, just as do straight lines and curves in Euclidean space. Triangles in the outside world are perceived as triangles and relationships between the perceived lengths of the sides and the angles between them are consistent with the behaviour of triangles in Euclidean space. If we have a geodesic triangle, with angles A, B, C and geodesic sides of length a, b, c, the sum of the angles A+B+C is always less than 180 degrees and $c^{2}>a^{2}+b^{2}-2ab\cos C$, analogous to the cosine rule $c^{2}=a^{2}+b^{2}-2ab\cos C$ of Euclidean space [109]. The prediction that all planes passing through the egocentre are totally geodesic, have the same negatively curved geometry and can be mapped in a one-to-one, onto, isometric fashion on to each other, is compatible with the structure of Euclidean space. Every plane passing through the origin in Euclidean space has the same geometry and every point in every plane can be transformed isometrically into every other plane passing through the origin. Thus, while the size–distance relationship introduced by the optics of the eye causes profound warping of the 3D perceived visual manifold (G,g), the warping does not disrupt a smooth, one-to-one, onto, invertible mapping between the perceived visual world and the actual world. While errors in depth perception can lead to illusions of various kinds, the warping itself does not induce paradoxes and ambiguities into the perception of 3D Euclidean space.

### 8.4. IIlusions

The Riemannian metric g(*r*, θ, φ) stored at every cyclopean coordinate in G-memory effectively anticipates the perceived size of an object as a function of its Euclidean distance r in the outside world. An error in estimated distance r, such as can be produced by top-down cognitive mechanisms, results in the encoded image-point vector(s) being stored at the wrong site(s) in G-memory. This leads to errors not only in the perceived depths of objects but also in their perceived sizes. Such errors can introduce perceptual illusions. For example, when a concave face mask is seen as convex because of a learned cognitive expectation that faces are convex in shape, the result is the well-known compelling illusion that the perceived convex face appears to oscillate back and forth when the concave mask is actually rotated at constant speed in one direction. In most cases, cognitive expectations are consistent with the outside world and provide a mechanism able to short-cut bottom-up visual processing. However, when they overrule estimates of depth derived from stereoscopic processing they can mislead the viewer into seeing illusory movements and shapes that are not actually there.

Also well-known are illusions of size associated with after images [93,94,95,96,118,119]. Emmert’s law stated in 1891 that *after-images are seen to double in size with each doubling of seen distance* [95]. If a person fixates a bright object for a long enough interval of time for the image to become ‘burnt’ on to the retina, it induces an after-image that can persist for many seconds even after the gaze is shifted to another point in the environment. Unlike an actual object in the environment whose perceived size decreases with increasing distance, the size of the after-image increases with increasing distance [118]. The after image of a bright disc projected on to a white wall further away than the bright disc appears as a dark disc increased in size. If the after-image is projected on to a sloping wall it is perceived as having an oval shape consistent with the varying distances to the after-image on the sloping wall [93].

According to our proposal, stereoscopic vision provides an estimate of the cyclopean coordinates (*r*, θ, φ) of the gaze point on the wall. The encoded retinal images of the wall (including the superimposed retinal after-image of the disc) are stored into G-memory at sites specified by the estimated cyclopean coordinates (*r*, θ, φ). The metric g(*r*, θ, φ) at those memory sites correctly anticipates the size of the retinal image of the wall but incorrectly anticipates the size of the retinal after-image. The after-image appears larger because it has been stored in a wrong G-memory site at a larger distance r relative to the true distance to the bright disc. The size of the after-image on the retina is appropriate for the actual distance to the bright disc but, because it is ‘burnt’ on to the retina, it does not change size when the depth of gaze is altered. The 1r anticipated change of size of the retinal image with increasing depth of gaze r does not occur and consequently, the after-image is perceived to increase in size in proportion to r, (i.e., the inverse of the 1r anticipated reduction in size) as described by Emmert’s law.

An incorrect estimate of the depth of gaze can disrupt Emmert’s law. Broerse et al. [93] found that when after-images are projected on to the slanting wall of an Ames room, instead of appearing oval, the after-images appear circular as if projected on to the illusory non-slanting wall of the perceived Ames room. Using functional magnetic resonance imaging (fMRI), Sperandio et al. [119] showed that activity in V1 associated with viewing an after-image is modulated by the incorrectly perceived size of the after-image even when the size of the retinal image remains constant. This is consistent with the above account based on the Riemannian geometry theory and with the proposal that the same brain areas are involved in both visual imagery and visual perception [120].

In monocular and pictorial imagery, depth is more or less a free parameter influenced by many factors depending on both the scene and the observer. Where a variation in estimated depth occurs attributable to top-down cognitive mechanisms, this will give rise to variations in the geometry of visual space as measured in different experiments, those involving illusions included. Nevertheless, the size–distance relationship of retinal images introduced by the optics of the eye is always present, giving rise to a fundamental invariant warping of the 3D perceived visual space that underlies all other measured perturbations of the geometry.

### 8.5. Measuring the Geometry of Perceived Visual Space

We indicated above our contention that it is the influence of top-down cognitive mechanisms that is in general responsible for the inconsistencies in experimental attempts to measure the geometry of perceived visual space. As addressed in Section 1, the findings of task and other dependencies led some researchers to call for reconsideration of the concept of visual space [19] or indeed its very existence [33]. Significant in this was the disparity found by Koenderink’s group between results of exocentric pointing and collinearity experiments on the one hand, and parallelity measures on the other. The three types of experiment were similar in that each involved two objects located in the horizontal plane of the observer’s eyes and placed in varying positions in relation to the observer and to each other. The exocentric pointing experiments [15,17] involved a target and a remotely-controlled pointer. The parallelity and collinearity experiments [18,19] involved two rods, one a reference rod and the other a remotely-controlled test rod to be aligned so as to appear parallel to or collinear with the reference. Subjects observed the objects in a series of differing object placements and, according to what they perceived, chose angular settings of pointer or test rod to fulfil the required task. These settings were measured and expressed as a deviation from the veridical (Euclidean) relationship between the objects.

All three experiments yielded deviations that varied systematically with the separation angle of the objects with respect to the observer and with their relative distances from the observer. While the pattern of variations was similar in the exocentric-pointing and collinearity experiments, the pattern of deviations in the parallelity experiment was different and the deviations were considerably larger than in the exocentric pointing and collinearity tests. The findings were later confirmed in a composite experiment by Doumen et al. [24] and led to the notion that there may not be a consistent geometry of visual space. Koenderink et al. [28] argued that to explain such data either the experiments are not appropriate for measuring geometry; or the geometry of visual space would have to be *contextual*, i.e., dependent on what is in the space; *momentary*, i.e., dependent on where the observer is fixating in the space; or *task dependent*, or perhaps all of these.

In our interpretation these data do not preclude the existence of a geometrically invariant perceived visual space. Based on the invariant Riemannian space (G,g) with metric determined by the size–distance relationship introduced by the optics of the eye we now use the simulated geodesics of Section 5 to explain qualitatively from first principles why deviations in the above experiments vary systematically with separation angle and distance ratio. We then suggest how the disparity in the size and the pattern of the deviations can be attributed to differing task constraints and computational strategies within the invariant curved space rather than to changes in the geometry itself.

Figure 5a,b shows spiral geodesics confined to the horizontal plane emanating from two different exocentric initial points in the plane. Consider a pointing device located at such an initial point. For any given target in the plane there will be a particular spiral geodesic that emanates from that initial point and passes through the target. Perceived from the egocentre, this geodesic will appear to be a constant speed straight line between the pointer and the target in the direction of the geodesic’s initial velocity vector at the pointer. In other words, due to the warping of the perceived horizontal plane, an observer will inaccurately aim the pointer in the direction of the initial velocity vector because this direction is perceived as being the constant speed straight-line direction to the target. The veridical direction is of course given by the constant speed straight line drawn in Euclidean space from the pointer to the target. The predicted pointing error is given by the angle between this line and the initial velocity vector for the geodesic joining the pointer and the target. It can be deduced from the spiral geodesics in Figure 5 that such pointing errors will vary systematically depending on the angle of separation at the egocentre between the pointer and the target and with their relative distance from the observer. We conclude that the geometry of (G,g) predicts a systematic variation in errors in both exocentric-pointing and collinearity tasks.

The simulated geodesics in Figure 5 also provide an explanation from first principles for systematic errors in the judgement of parallelity. For any given positions of the test rod and the reference rod, there will be a geodesic passing through both points. Since geodesics are perceived to be constant speed straight lines, it follows that any vector parallel translated along a geodesic forms a set of vectors that are perceived as being parallel to each other. They are not parallel in the outside world. The angle between a vector at an initial point p and this vector parallel translated along the geodesic to any other point q provides a measure of the angular error between perceived parallelity and veridical parallelity. It can be deduced from the geodesics in Figure 5 that this error varies systematically with the angle of separation at the egocentre between such points p and q. Again, the geometry of (G,g) predicts a systematic variation in errors in the parallelity task.

The underlying geometry described by the geodesics in Figure 5 is the same for all three types of experiment. Why then do the pattern of deviations and the size of the deviations measured experimentally in the parallelity task (and hence the geometry of the visual space computed from those data) differ from those measured experimentally in the exocentric and collinearity tasks? We will now argue that this disparity is accounted for not by a varying geometry but by differences in the computational strategy required within the invariant perceived visual space (G,g) to achieve the differing task goals.

In the exocentric-pointing and collinearity tasks the target and pointer are sufficiently far removed from each other that the observer has to switch gaze back and forth between them in order to decide on an appropriate setting for the pointer. While looking at the target, the observer estimates the perceived straight-line direction to the memorized location of the pointer and, while looking at the pointer, the observer estimates the perceived straight-line direction to the memorized location of the target. If we consider a spiral geodesic (Figure 5) that connects pointer and target, the perceived directions correspond to the vectors tangent to the geodesic at the positions of the pointer and the target, respectively. These are not collinear in the Euclidean outside world. But to the observer one is taken to be the negative of the other, aligned on the same perceived straight line between pointer and target. We suggest that the observer adopts a strategy of iteratively averaging these estimates by looking back and forth between target and pointer in order to arrive at a setting. When the negative of the estimated straight-line direction from target to pointer is averaged with that from pointer to target the resulting direction is closer to veridical than either of the estimated directions taken separately. Nevertheless, because of the spiral shape of the geodesic, the averaged direction still deviates from veridical and that deviation varies with separation angle and relative distance (with an interaction between them). These predicted variations have a similar size and pattern to those measured for the exocentric pointing and collinearity tasks of Doumen et al. [24].

This account of findings accords with the suggestion by Doumen et al. [26] that for an exocentric-pointing task an observer needs to make a judgement about the position of both the pointer and the target whereas, for a parallelity task, the observer does not have to know the actual positions to perform the task. In the parallelity task, the observer has to look back and forth between the reference rod and the test rod but, unlike in the exocentric-pointing and collinearity tasks, does not have to estimate the perceived straight-line path between them. Here the likely strategy is to note the angle of the reference rod relative to a locally available estimated reference frame, for example a wall. The angle of the test rod can then be adjusted to match that direction relative to the same reference frame. In Euclidean space, a local external reference frame such as a wall is invariant and the strategy would work. However, as shown by the simulated geodesics in Figure 5 and depicted in Figure 3, the perceived distance to a flat wall varies logarithmically with Euclidean distance and so the perceived wall is negatively curved towards the observer. This occurs even when the curve in the outside world corresponds to a geodesic that appears straight. Visual external reference frames in a parallelity task introduce systematic errors between perceived and veridical parallelity. This is simply because all visual reference frames are warped by the geometry of the underlying perceived visual manifold (G,g). Consequently, according to the explanation given here, due to the warped geometry of (G,g) the angle of the test rod will deviate further from veridical the larger the separation angle between the rods. The deviation will be positive on the left hand side and negative on the right hand side, passing through zero for zero separation angle (straight ahead). This prediction produces the same size and pattern of parallelity errors as measured experimentally by Doumen et al. [24]. Indeed, according to this explanation, because it is the curved geometry of (G,g) causing the warping of the perceived visual reference frame that is in turn responsible for errors in judgement of parallelity, it follows that the parallelity task gives a better measure of the geometry of (G,g) than do the exocentric-pointing or collinearity tasks. Those tasks do not provide an accurate measure of the geometry.

Doumen et al. [25,26] extended the exocentric pointing experiment beyond the horizontal plane to include variations in the positions of pointer and target in the vertical dimension both above and below the height of the observer’s eyes. They measured the deviations from veridical pointing directions in the horizontal and vertical planes passing through the pointer (termed slant and tilt respectively) and found that the vertical separation angle had no effect on the deviations of the slant, but did have a linear effect on tilt. Despite the complication that neither the horizontal plane nor the vertical plane in which slant and tilt are measured pass through the egocentre, the size and pattern of 3D pointing errors measured by Doumen et al. [25] can be accounted for by the same computational strategy within (G,g) described above for exocentric pointing and collinearity in the horizontal plane; that is, by averaging the perceived straight-line directions from target to pointer and from pointer to target.

The perceived straight-line directions are determined as follows. Given three points in the outside world corresponding to the egocentre, the position of the pointer and the position of the target, there exists a unique plane that passes through all three points. As shown in Section 5, the same pattern of geodesics given in Figure 5 for the horizontal plane and the same warped geometry of the perceived visual manifold exists for every plane that passes through the egocentre. Thus, since the unique inclined plane just described does pass through the egocentre, there exists in it a pattern of spiral geodesics isomorphic with those in Figure 5 and, among those, there exists a unique spiral geodesic that passes through the positions of both the pointer and the target. When seen from the egocentre, this unique geodesic is perceived to be a constant-speed straight line connecting pointer to target (and target to pointer). Tangent vectors to the geodesic are collinear with the perceived straight line. If the tangent vectors at the pointer and at the target are averaged to obtain the setting of the pointer and then the direction of the pointer is projected into the horizontal and vertical planes passing through the pointer, we deduce that the same size and pattern of slant and tilt deviations as described by Doumen et al. [25] are obtained. This includes the change in sign in the tilt deviations depending on whether relative distance is greater than or less than one.

Doumen et al. [25,26] concluded that the structure of visual space is distorted in both the horizontal and vertical directions but that the deformation is not isotropic. This raises an important point about the use of the terms *isotropy* and *anisotropy*. As explained by Wagner and Gambino [4], “anisotropy” as used in physics refers to variations in the properties of space as a function of direction from the observer. Lee [57] (Chapter 3, p. 33) states, using standard terminology, that a Riemannian manifold M is homogeneous if its geometry is the same at every point. Given a point p∈M, the Riemannian geometry is isotropic at p if the geometry is the same in every direction out from the point p∈M. Clearly, a homogeneous Riemannian manifold that is isotropic at one point is isotropic at every point; in that case, one says M is homogeneous and isotropic. A homogeneous Riemannian manifold looks geometrically the same at every point, while an isotropic Riemannian manifold looks the same in every direction at every point.

Applying this terminology to the Riemannian manifold (G,g) we can say it is neither homogeneous nor isotropic. In general, it is inhomogeneous and anisotropic but with special restrictions within the manifold this does not apply. All visual spheres are both homogeneous and isotropic. At the egocentre (and only the egocentre), (G,g) is isotropic; as far as the observer is concerned the geometry of (G,g) looks the same in all directions. As can be seen in Figure 5 and Figure 8, the radial geodesics emanating in all directions from the egocentre are accelerating straight lines. These show that while the perceived distance to a target on a radial line is underestimated, the perceived direction from the egocentre to the target and from the target back to the egocentre are collinear and, therefore, an observer can aim accurately at such a target. In other words, egocentric pointing is easy since the geometry of (G,g) is isotropic at the egocentre (and only at the egocentre) and the warping of (G,g) does not disrupt an observer’s ability to aim accurately at a target located anywhere in the 3D outside world. However, for any other point q∈(G,g) the geometry of (G,g) is anisotropic. As a consequence, exocentric pointing is not a trivial task as the warping of (G,g) impedes the observer from pointing accurately from q∈(G,g) to a target located anywhere (other than the egocentre) in the 3D outside world.

Despite the results of experimental attempts to measure perceived visual space being fraught with the obscuring effects of constraints and strategies involved in the testing, the systematic deviations found in exocentric-pointing, collinearity and parallelity tasks speak to an anisotropy of visual space that is consistent with (G,g). It is important to understand that anisotropy does not preclude invariant geometry and, as our simulations show, (G,g) is both. It is our contention that the geometry of an invariant and anisotropic (G,g) will underlie all attempts to measure perceived visual space, so the revelation of anisotropy in such results is no surprise.

### 8.6. 2D versus 3D Representations

Some authors hold that the 2D perspective images projected on to the retinas do not have to be transformed into a 3D representation to account for perceptual phenomena. For example, Glennerster and colleagues [51] argue that a view-based manifold of 2D images can explain human perception in their expanding virtual room experiment and, using optical flow of 2D retinal images, can account for navigation from image to image in a 3D environment. Erkelens [116,121] has shown that a 2D perspective structure of visual space can account for straight lines in the 3D environment being perceived as straight lines. He demonstrated that collinearity but not parallelism is preserved in perspective space and that angles are not invariant under translation and rotation, properties of visual space shown experimentally. Also, according to Gilson and Glennerster [122], in constructing an immersive 3D virtual environment using a head-mounted stereo display it is necessary to ensure that the stereo-projected light rays into each eye from points in the virtual environment match the angles of light rays entering each eye from corresponding points in the actual 3D space as the person moves about in the virtual environment. In other words, the simulator has to present veridical 2D perspective projections of the 3D environment to each eye in order to create the illusion of moving about in an actual 3D world. It would be easy to conclude from this that veridical 2D view-based perspective projections are all that is required to form a representation of visual space. This may well be so for monocular vision and for looking into pictures. However, for binocular vision, whether in an actual 3D environment or in a 3D stereo immersive virtual reality, this does not take into account the ability of the nervous system to employ stereopsis (triangulation and retinal image disparity) to obtain an estimate of Euclidean distance to the array of points in the environment projecting on to each of the left and right retinal hyperfields. In this circumstance, the encoding of the projected 2D images on the retinas is augmented with depth information. Even during the brief interval of a single fixed gaze from a fixed point in the environment, the nervous system can form a 3D representation of the 2D visible curved surfaces of objects in the 3D environment. Because of this, as described in Section 7.2, by reciprocally mapping place-encoded images on to each other, the nervous system can remove occlusions and construct a cognitive (fibre bundle) model of the 3D environment seen from any place with the correct 3D perspective.

### 8.7. Visuospatial Memory

When looking at the outside world from any one place, it is possible, based on the experience of having seen the same local environment from many other places, to visualize the 3D shapes of objects in the environment with the correct perspective as seen from that place. We claim that the fibre-bundle structure of visuospatial memory described in Section 7, together with its vector-bundle morphisms, forms this type of 3D holographic representation of the environment seen from any place in the environment.

Given that vector-bundle morphisms $H=\left[H_{1}\left(p_{i},p_{j}\right),H_{2}\left(q_{p_{i}},q_{p_{j}}\right)\right]$ illustrated in Figure 11 can be wired-in to the visual system, it follows that a place-encoded visual image acquired through visual scanning at one place in the environment and stored in one vector bundle can be transformed into place-encoded visual images seen from other places in the environment stored in other vector bundles. In other words, as well as filling in occlusions, the vector-bundle morphisms transform the perspective of the image so it becomes appropriate for the new place. By transforming between each and every vector bundle in this way, the vector-bundle morphisms can fill in occlusions in all place-encoded images in every vector bundle for every place p ∈ P in the environment. As a result of transformations occurring continuously between vector bundles (the visual cortex is known to be active even when the eyes are shut or in the dark [123]), any one vector bundle can include images transformed from all other vector bundles (i.e., vector-bundle partitions of place-encoded visuospatial memory).

One has only to look at an object and then close the eyes to appreciate that the directly-encoded image of an object in the environment is more intense and contains more detail than the memorized image. Similarly, when visualizing the 3D shape of an object seen from a fixed place, or visualizing the layout of a familiar room on the other side of an opaque wall, the visualized images are less intense and contain less information than the directly-encoded images. Nevertheless, the visualized images and the directly-encoded images do not interfere and can be perceived separately or together. This can be accounted for within the fibre bundle by the proposal that, as well as being partitioned into vector bundle sub-memories, visuospatial memory consists of at least three layers within each vector bundle. Like images composed of layers in image-processing software, the three layers can be superimposed and perceived together, or they can be separated and perceived separately. We propose that the first layer represents the neural activity in cortical hypercolumns held in working memory during the interval of fixed-gaze encoding of the left and right retinal images associated with that fixed point of gaze. This is the most intense and detailed image. The second layer corresponds to the visual images stored into the G-memories within the vector bundles. The third layer consists of those images transformed into each vector bundle from every other vector bundle by vector-bundle morphisms. Memorized images in layers two and three are less intense and less detailed than the directly-encoded images in layer one.

### 8.8. Visuospatial Representation as a Philosophical Issue

With respect to differences between “perceiving” and “sensing” and the philosophical distinction between “direct perception” and “indirect perception” [124], the Riemannian geometry of 3D binocular perception developed in this paper is firmly in the school of indirect perception. We have addressed such issues in the past in our paper entitled “Berkeleian principles in ecological realism: an ontological analysis” [125]. Therein we have argued that Gibson’s notions of direct perception and affordances [126] (like the proposal of George Berkeley (1685–1753) that the external world is “merely ideas in the mind” and the proposal of Thomas Reid (1710–1796) that “what we see, what we visualize, what we believe of an object, is that object’s true reality”) lead to the conclusion that we do not need a brain to perceive reality but require only a direct perception of it. While there is no unambiguous way to measure the conscious perceptions of other people, we do of course have our own perceptual experience of visual space to examine. How this conscious perception is related to neural processes in the brain is an unanswered question. It may be a metaphysical question or consciousness may be an illusion, as argued by Dennett [127]. Nevertheless, it seems to us that an individual’s conscious visual perceptions are inexorably linked to physical processes in the eye and brain. After all, people are rendered blind by poking out their eyes and various disorders of visual perception can be traced to lesions of various types in the brain. Persuaded by this, we hold that the geometrical structure of perceived visual space can be mapped to its representation by physical processes within the brain. Of course given the non-linear limit-cycle behaviour of neurons and neural circuits and the activity-driven synaptic plasticity of thousands of millions of synaptic connections within the brain’s neural networks, such physical processes are notoriously difficult to describe. Nevertheless, we submit that Riemannian geometry provides the best mathematical framework for investigating the non-linear dynamical brain processes yet to be understood.

## 9. Future Directions

Manifolds, vector fields, metrics, curvature tensors, vector bundles, fibre bundles and so forth are constructs from Riemannian geometry that we have shown to be of value in describing computational processes underlying the warped geometry of 3D binocular visual perception. By defining links between experimental observations and structures within Riemannian geometry (e.g., encoding of retinal image features in V1 as vector fields over a manifold) we have been able to employ the theorems and propositions of Riemannian geometry to illustrate some of the computational issues underlying 3D binocular visual perception. There is, however, a caveat. Our theory should not be taken as implying that the visual system actually performs geometric computations. The nervous system may well have evolved its own methods of processing and transforming visual signals; e.g., by means of networks of neural adaptive filters [83]. The value of Riemannian theory lies in its ability to reveal the computational issues involved in transforming retinal images into 3D perceptions of the world and in its ability to demonstrate the logical feasibility that such computational issues can be resolved. As described by Marr [90], how neural circuits actually implement these computational processes requires a second stage of analysis beyond the computational theory.

Despite the detail in the theory there are many phenomena of visual perception that are yet to be addressed. For example, the issue of the perception of accelerating straight lines and accelerating planar surfaces mentioned in Section 8.1 needs further development. In physics, the accelerations of geodesic spray fields are often augmented with accelerations attributable to potential energy conservative force fields such as gravity. This gives rise to accelerating trajectories that deviate from geodesic trajectories. An analogous theory promises to account for perception of accelerating straight lines and accelerating planar surfaces within the curved visual manifold (G,g). While the Riemannian theory depends on stereopsis to obtain measures of Euclidean distance, no explanation is given (other than referring to top-down mechanisms) for monocular depth perception or the ability to perceive depth in pictures. Details of colour perception within the framework of extracting SVD retinal image features or of adaptation of the size of retinal receptive fields to changing levels of luminosity have not been discussed. The ability to predict optical flow when moving in an unfamiliar environment is yet to be explored, as is the ability to construct a 3D perception of a novel environment from recognition of familiar objects within that environment. Clearly there is more to be done, particularly with regard to object recognition.

One important direction for future work is described by Wagner and Gambino [4]. In conjunction with their meta-analysis of the anisotropy and geometry of visual space, these authors review research, both old and new, showing that visual space is strongly influenced by context, judgement methods, instructions, and stimulus conditions. They argue, however, that this does not mean we should abandon the concept of visual space which always exists within our visual experience even though its exact geometry can change with circumstances. They do not agree with people who say we should abandon the concept of visual space just because there are contextual effects and state that “the goal of our studies should be to specify exactly how metric estimates of visual space change in response to these contextual variables. Mathematically, we should incorporate these other factors into our metric equations to predict perceptual judgements” [4] (p. 585).

The metric equations described in this paper suggest a basis from which such future work could be constructed. If top-down cognitive mechanisms based on experiential knowledge of size–distance relationships were to modify the cyclopean co-ordinates associated with image points in the environment, this would temporarily retune the sites in the gaze-based *G*-memory where the vectors of retinal image features are stored. This will alter the size, depth and shape of the perceived image. In the case of the Ames room or the virtual expanding room, for example, the changed geometry of the perceived image will be altered to match the expected perceived shape of rooms derived from previous experience. Recognition of an object with recall of its associated properties may take precedence in terms of functional (survival) value over verisimilitude. This could explain why a cognitive overlay of object recognition based on a probabilistic analysis of past experience may sometimes overrule the perception of size and shape based on binocular vision and stereopsis. Perhaps in the future we can come to see the inhomogeneous geometry associated with cognitive overlays simply as the predictable perturbations of the underlying invariant geometry introduced by the optics of the eye.

Meanwhile, the proposed structure of fibre bundles with wired-in vector-bundle morphisms (Section 7 and Figure 11) already contributes a substantial account of how we see the way we do. It provides a geometric model of the visual environment as seen from any place and does so in a way that is neurally feasible. It allows the 3D shapes of objects in the environment to be visualized in the correct perspective no matter from where they are viewed. By virtue of its Riemannian structure, it accounts for the perceived size of such objects. It incorporates the laws of optical flow and thus allows visualization of the changing 3D images associated with progression of the head along any path in the environment. Moreover, it lends itself to other visual roles. For example, it can play a role in object recognition by correcting for changes in perceived size and shape as a function of position and orientation of the object relative to the observer. It accounts for an ability to change the position of a reference metric (perceptual tape measure, Section 6.1) in the perceived visual manifold. It can function as a model of the environment in model-based reinforcement learning [128,129,130,131]. It can play a role in seeing the world from another person’s point of view. It can be involved in the acquisition of new motor skills through imitation and mental imagery. It can allow familiar 3D environments to be visualized as if seen from a place not previously experienced enabling, for example, the drawing of a plan of furniture layout in a familiar house as if seen through the roof from a point above the house. Also, as we will show in a subsequent paper, it can be integrated with our previously published Riemannian geometry theory of human movement [52] and our Basic Unit of Motor Production (BUMP) theory of response planning [132,133] to explicate the proprioceptive-visual and visual-proprioceptive transformations and the selection of task-compatible movement synergies required in the planning and execution of visuospatial motor tasks.

To account for the results of their expanding virtual room experiment, Glennerster and colleagues [51,97] have proposed a view-based approach to spatial representation in human vision, suggesting that observers use view-based methods [134] to guide their actions and to represent the spatial layout of the scene. They also write that “a robot or animal stores views or ‘snapshots’ of a scene and records something about the motor output required to move between one view and the next, without integrating this information into a Cartesian map” [51] (p. 196). In other words, they suggest that as persons move about in a room they form a *manifold of views* of the room. Each point in the manifold corresponds to a single view and neighbouring points correspond to views from neighbouring vantage points. The goal is to navigate across the manifold of images.

The Riemannian geometry theory of visuospatial representation described in this paper has much in common with this view-based approach. The notion of a geometric fibre bundle is analogous to a “manifold of views” except that the place-encoded images can be 3D. The possible role of optical flow in navigating a person through a visual environment is similar in both theories. However, the fibre-bundle theory goes beyond the view-based approach by taking the warped geometry of the 3D perceived visual manifold into account. Moreover, by integration with our Riemannian geometry theory of human movement [52], a generalized fibre-bundle conceptualization of visuomotor performance promises to quantify Glennerster’s “something about the motor output required to move between one view and the next.”

## Appendix A. Extraction of Non-Linear Orthogonal Visual Image Features Using Singular Value Decomposition (SVD)

### A1. Extraction of Linear Orthogonal SVD Image Features

Consistent with the work of Simoncelli and Olshausen [76] mentioned in Section 2.5, extraction of orthogonal stochastic feature signals from images on retinal hyperfields by the hypercolumns of V1 can be seen as a two-stage process. The first stage, analogous to the principal components stage of Simoncelli and Olshausen, is accomplished by singular value decomposition (SVD) [135,136]. This is achieved by slow tuning of synaptic weights connecting left retinal hyperfields to left ocular dominance minicolumns and corresponding right retinal hyperfields to right ocular dominance minicolumns within the same hypercolumns. SVD learning rules are described by many [137,138,139,140,141] and, with the tuning of the synaptic weights occurring over several weeks, the process is similar to Hebbian learning [142] in which, basically, synapses that fire together wire together with *winner take all* collateral inhibition.

The resulting synaptic weights for each ocular dominance minicolumn correspond to a rank-one matrix in SVD of the average image falling on the hyperfield over an interval of several weeks or more. Each rank-one matrix of synaptic weights corresponds to an orthogonal *stochastic* feature (i.e., a rank-one orthogonal hyperfield image) extracted from the averaged hyperfield image. The synaptic weights for left ocular dominance minicolumns in a hypercolumn are tuned in this way to extract orthogonal stochastic feature signals from the left retinal hyperfield projecting to the hypercolumn. Similarly, the synaptic weights for right ocular dominance minicolumns in the same hypercolumn are tuned to extract orthogonal stochastic feature signals from the corresponding right retinal hyperfield projecting retinotopically to the same hypercolumn. Since the stochastic amplitude spectrum of images falling on each hyperfield has a spatial-frequency bandwidth of only 10 cycles per degree or less, it follows that 10 rank-one matrices of synaptic weights is more than sufficient to encode the correlations within each stochastic hyperfield image. For a simulator demonstration of the relationship between bandwidth of a stochastic signal and the number of singular values required, see [83]. The number 10 is not critical in what follows but it is used here to give a ball-park indication of the number of minicolumns required.

The synaptic weights tune so slowly over several weeks that during the interval of a single fixed gaze they can be regarded as fixed or wired-in to the minicolumns. Thus, the image $I_{L}$($\hat{\theta }$,$\hat{\varphi }$) falling on a left retinal hyperfield during the interval of a fixed gaze is decomposed by the fixed synaptic weights connecting it to 10 left ocular dominance minicolumns into a set of 10 orthogonal rank-one image features. The set of 10 orthogonal stochastic feature signals encoded by the temporospatial pattern of neural activity within the 10 left ocular dominance minicolumns corresponds to the 10 singular values $\left(\sigma_{L1},\;\cdots ,\sigma_{L10}\right)$ of the SVD. Similarly, the image $I_{R}$($\hat{\theta }$,$\;\hat{\varphi }$) falling on the corresponding right retinal hyperfield during the same interval of fixed gaze is decomposed by the fixed synaptic weights connecting it to 10 right ocular dominance minicolumns in the same hypercolumn into a set of 10 orthogonal stochastic feature signals represented by 10 singular values $\left(\sigma_{R1},\;\cdots ,\sigma_{R10}\right)$ encoded by the temporospatial pattern of activity in the 10 right ocular dominance minicolumns.

Each singular value $\sigma_{i}$ measures, in a matched-filter fashion, the strength of the corresponding rank-one stochastic feature in the image I($\hat{\theta }$,$\;\hat{\varphi }$) falling on the hyperfield during the interval of a fixed gaze. In other words, the hyperfield image is decomposed into 10 rank-one orthogonal hyperfield images that, if added together in the appropriate proportions $\sigma_{i}$, would recreate the hyperfield image. Because of disparity between left and right retinal images, the point in the environment projecting on to a left retinal hyperfield is shifted relative to the point in the environment projecting on to the corresponding right retinal hyperfield. When gaze is shifted to a new gaze point, the retinal images change and a new set of stochastic features is extracted by the rank-one matrices of fixed synaptic weights on each minicolumn producing a different temporospatial pattern of neural activity in the minicolumns.

### A2. Extraction of Non-Linear Orthogonal SVD Image Features

The second stage of visual image feature extraction described by Simoncelli and Olshausen [76] takes non-Gaussianity (non-linearities) of stochastic images into account. We have shown previously [83] that this can be achieved within an SVD adaptive filter using only a small number of extra adaptive synaptic weights. The procedure takes advantage of the fact that a non-linearity described by a second-order Volterra kernel is equivalent to a linear filter in cascade with a squarer, while a non-linearity described by a third-order Volterra kernel is equivalent to a linear filter in cascade with a cuber [143,144]. This allows a considerable reduction in the number of adaptive parameters required given that a Volterra model of a third-order non-linear dynamic filter typically requires hundreds of thousands of adaptive parameters. Using the squarer and cuber method, non-linear orthogonalization can be achieved with only 60 extra adaptive parameters as demonstrated previously with a third-order non-linear SVD singular-vector adaptive filter that was thoroughly tested using signals with a variety of non-white spectral density functions and non-Gaussian amplitude probability density functions [83].

Accordingly, to account for skewness and kurtosis associated with the presence of third- and fourth-order moments, our second stage process takes the singular values $\left(\sigma_{L1},\;\cdots ,\sigma_{L10}\right)$ and $\left(\sigma_{R1},\;\cdots ,\sigma_{R10}\right)$ from the first stage and begins by squaring and cubing each $\sigma_{i}$. It then employs a Hebbian-like learning rule (i.e., least mean square (LMS) adaptive filter algorithm [145,146,147]) to tune minicolumn synaptic weights so as to orthogonalize the linear, squared, and cubed singular value signals $\left(\sigma_{i}^{1}\left(t\right),\sigma_{i}^{2}\left(t\right),\sigma_{i}^{3}\left(t\right)\right),\;i=1,\ldots ,10$ extracted from left and right hyperfields. This gives a total of 30 stochastic non-linear orthogonal feature signals $\Sigma_{L}=\left(\Sigma_{L1},\cdots ,\Sigma_{L30}\right)$ for the left retinal hyperfield image encoded by 30 left ocular dominance minicolumns within the hypercolumn, and 30 stochastic non-linear orthogonal feature signals $\Sigma_{R}=\left(\Sigma_{R1},\cdots ,\Sigma_{R30}\right)$ for the corresponding right retinal hyperfield encoded by 30 right ocular dominance minicolumns within the same hypercolumn. This type of spatiotemporal orthogonalization not only encodes the non-Gaussianity of stochastic hyperfield images but, as shown in [83], it also tracks changes in the probability density and spectral density functions of hyperfield images that occur over time.

## Appendix B. Computing Curvatures

### B1. Gaussian Curvatures and Sectional Curvatures

Suppose $\left(\tilde{G},\tilde{g}\right)$ is a 2D submanifold isometrically embedded ι: $\left(\tilde{G},\tilde{g}\right)\to \left(G,g\right)$ in the 3D ambient perceived visual manifold (G,g). At any point $q\in \left(\tilde{G},\tilde{g}\right)$ the 3D tangent space $T_{q}G$ is called the ambient tangent space over the 2D submanifold $\left(\tilde{G},\tilde{g}\right)$. The 3D ambient tangent space $T_{q}G$ can be partitioned into two g-orthogonal vector subspaces, the 2D tangent space $T_{q}\tilde{G}$ tangent to the submanifold $\left(\tilde{G},\tilde{g}\right)$ and the 1D tangent space $N_{q}\tilde{G}$ g-normal to the 2D submanifold $\left(\tilde{G},\tilde{g}\right)$. Suppose there are two vector fields X and Y in the 2D tangent bundle $T\tilde{G}$ over the submanifold $\left(\tilde{G},\tilde{g}\right)$. These two vector fields can be extended arbitrarily into the 3D tangent bundle TG over the ambient manifold (G,g). The covariant derivative $\nabla_{X}Y$ of the extended vectors X and Y in the ambient manifold at the point $q\in \left(\tilde{G},\tilde{g}\right)$ can be computed because, as described in Section 4.2, the geodesic spray field $f_{2}\left(q,\dot{q}\right)$ is known for every $\left(q,\dot{q}\right)$ in the ambient tangent bundle TG. The covariant derivative $\nabla_{X}Y$ is a vector in the 3D ambient tangent space $T_{q}G$. This vector can be projected into the g-orthogonal spaces $T_{q}\tilde{G}$ and $N_{q}\tilde{G}$. The component in $T_{q}\tilde{G}$ is the covariant derivative ${\tilde{\nabla }}_{X}Y$ in the submanifold $\left(\tilde{G},\tilde{g}\right)$ compatible with the unknown metric $\tilde{g}$. The component of $\nabla_{X}Y$ projected into the normal space $N_{q}\tilde{G}$ is denoted by II(X,Y) and is known as the *second fundamental form* at $q\in \left(\tilde{G},\tilde{g}\right)$.

The second fundamental form II(X,Y) is independent of the arbitrary extension of the vectors X and Y into TG, it is bilinear over real-valued smooth functions $C^{\infty }\left(\tilde{G}\right)$ on $\left(\tilde{G},\tilde{g}\right)$, and it is symmetrical in X and Y. From the projection of $\nabla_{X}Y$ into $T_{q}\tilde{G}$ and $N_{q}\tilde{G}$, we obtain the Gauss formula:

(A1) $$\nabla_{X}Y={\tilde{\nabla }}_{X}Y+II\left(X,Y\right).$$

The Gauss formula relates the connection $\tilde{\nabla }$ on the submanifold $\left(\tilde{G},\tilde{g}\right)$ to the connection ∇ on the ambient manifold (G,g).

The Gauss equation can be derived from the Gauss formula. For any vector fields X,Y,Z,and W in the 2D tangent bundle $T\tilde{G}$ over the submanifold $\left(\tilde{G},\tilde{g}\right)$, the Gauss equation,

(A2) $$R_{m}\left(X,Y,Z,W\right)={\tilde{R}}_{m}\left(X,Y,Z,W\right)-{\langle II\left(X,W\right),II\left(Y,Z\right)\rangle }_{g}+{\langle II\left(X,Z\right),II\left(Y,W\right)\rangle }_{g}$$

relates the Riemann curvature tensor ${\tilde{R}}_{m}\left(X,Y,Z,W\right)$ of the submanifold $\left(\tilde{G},\tilde{g}\right)$ at the point $q\in \left(\tilde{G},\tilde{g}\right)$ to the Riemann curvature tensor $R_{m}\left(X,Y,Z,W\right)$ of the ambient manifold (G,g) at the same point q∈(G,g). For any two vectors X and Y spanning the 2D vector space $T_{q}\tilde{G}$ tangent to the submanifold $\left(\tilde{G},\tilde{g}\right)$ at the point $q\in \left(\tilde{G},\tilde{g}\right)$, the *Gaussian curvature* of the submanifold at that point is given by:

(A3) $$\tilde{K}\left(X,Y\right)=\frac{\tilde{R_{m}}\left(X,Y,Y,X\right)}{{\left\|X\right\|}_{\tilde{g}}^{2}{\left\|Y\right\|}_{\tilde{g}}^{2}-{\langle X,Y\rangle }^{2}}.$$

If the vectors X and Y are g-orthogonalized using the Gramm–Schmidt algorithm, we obtain two orthonormal vectors $e_{1}$ and $e_{2}$ spanning $T_{q}\tilde{G}$. The Gaussian curvature of the submanifold at the point $q\in \left(\tilde{G},\tilde{g}\right)$ can then be written as:

(A4) $$\tilde{K}\left(e_{1},e_{2}\right)={\tilde{R}}_{m}\left(e_{1},e_{2},e_{2},e_{1}\right).$$

We have applied the above relationships to curvatures of the perceived visual manifold in Section 5. There we construct a family of geodesics in the outside world emanating from an initial point q=α(0) with unit initial velocity vectors $v_{i},\;i=0,1,\cdots ,35$ confined to the plane II in the tangent space at the initial point q=α(0) spanned by two specified orthonormal vectors $e_{1}\;\mathrm{and}\;e_{2}$. These geodesics sweep out a 2D surface $S_{II}$ in the outside world called the *plane section determined by the plane II.* Any geodesic γ(t) contained in $S_{II}$ emanating from the initial point q=γ(0) is, by construction, also a geodesic in the ambient 3D outside world. Thus at the initial point q both the covariant derivative ${\tilde{\nabla }}_{\dot{\gamma }}\dot{\gamma }$ in $S_{II}$ and the covariant derivative $\nabla_{\dot{\gamma }}\dot{\gamma }$ in the ambient outside world are zero.

Thus, from the Gauss formula Equation (A1), the second fundamental form II vanishes at the initial point q. Consequently, from the Gauss equation Equation (A2), it follows that,

(A5) $$R_{m}\left(e_{1},e_{2},e_{2},e_{1}\right)={\tilde{R}}_{m}\left(e_{1},e_{2},e_{2},e_{1}\right),$$

where, by definition, $K\left(e_{1},e_{2}\right)=R_{m}\left(e_{1},e_{2},e_{2},e_{1}\right)$ is the *sectional curvature* of the ambient manifold (G,g) at the initial point q and $\tilde{K}\left(e_{1},e_{2}\right)={\tilde{R}}_{m}\left(e_{1},e_{2},e_{2},e_{1}\right)$ is the *Gaussian curvature* of the plane section $S_{II}$ at the initial point $q\in S_{II}$. This is a special property of the plane section $S_{II}$ constructed as described. It does not apply to embedded submanifolds in general. The total curvature of the perceived visual manifold at the initial point q∈(G,g) is equal to the sum of the sectional curvatures of all the plane sections at that point.

### B2. Principal Curvatures, Principal Directions and Perceived Curvatures

The relationships presented in Section 6.3 derive from the following reasoning in Lee [57] (Chapter 8). Since $E_{1}$ and $E_{2}$ in $T_{q}\tilde{G}$ are tangent to the submanifold at $q\in \left(\tilde{G},\tilde{g}\right)$ while n is g-orthogonal to the submanifold at the same point, it follows that ${\langle n,\;E_{1}\rangle }_{g}=0$, so we can write:

$0=\nabla_{E_{1}}{\langle n,E_{1}\rangle }_{g}={\langle \nabla_{E_{1}}n,\;E_{1}\rangle }_{g}+{\langle n,\;\nabla_{E_{1}}E_{1}\rangle }_{g}$.

Therefore:

${\langle \nabla_{E_{1}}n,\;E_{1}\rangle }_{g}=-{\langle n,\;\nabla_{E_{1}}E_{1}\rangle }_{g}$,

$=-{\langle n,\;{\tilde{\nabla }}_{E_{1}}E_{1}+h\left(E_{1},E_{1}\right)n\rangle }_{g}$, (using the Gauss formula and Equation (37))

$=-{\langle n,\;h\left(E_{1},E_{1}\right)n\rangle }_{g}$, (because ${\tilde{\nabla }}_{E_{1}}E$ is g-orthogonal to n)

$=-h\left(E_{1},E_{1}\right){\langle n,n\rangle }_{g}$,

$=-h\left(E_{1},E_{1}\right)$, (because ${\langle n,n\rangle }_{g}=1$)

$=-{\langle SE_{1},E_{1}\rangle }_{g}$, (using Equation (38))

$=-{\langle \kappa_{1}E_{1},E_{1}\rangle }_{g}$, (using Equation (39))

$=-\kappa_{1}{\langle E_{1},E_{1}\rangle }_{g}$,

$=-\kappa_{1}$, (because ${\langle E_{1},E_{1}\rangle }_{g}=1$).

Thus, from ${\langle \nabla_{E_{1}}n,\;E_{1}\rangle }_{g}=-{\langle SE_{1},E_{1}\rangle }_{g}$ we obtain:

(A6) $$\nabla_{E_{1}}n=-SE_{1}=-\kappa_{1}E_{1}.$$

In other words, the negative of the perceived rate of change $\nabla_{E_{1}}n$ of the unit normal vector n for movement in the direction $E_{1}$ tangent to the submanifold is collinear with $E_{1}$ and its g-norm equals the perceived maximum principal curvature $\kappa_{1}$ of the submanifold at that point. The principal direction $E_{1}$ can be easily detected because the perceived rate of change $\nabla_{E_{1}}n$ of the unit normal vector n is collinear with the direction of movement only for the principal direction $E_{1}$. By similar reasoning:

(A7) $$\nabla_{E_{2}}n=-SE_{2}=-\kappa_{2}E_{2}.$$

Since the shape operator S is symmetrical, the direction $E_{2}$ is g-orthogonal to $E_{1}$ in the 2D tangent space $T_{q}\tilde{G}$.

Substituting the principal directions $E_{1}$ and $E_{2}$ into the Gauss equation Equation (A2) we obtain:

(A8) $$R_{m}\left(E_{1},E_{2},E_{1},E_{2}\right)={\tilde{R}}_{m}\left(E_{1},E_{2},E_{1},E_{2}\right)-h\left(E_{1},E_{2}\right)h\left(E_{2},E_{1}\right)+h\left(E_{1},E_{1}\right)h\left(E_{2},E_{2}\right).$$

We define:

$h_{11}=h\left(E_{1},E_{1}\right)={\langle SE_{1},E_{1}\rangle }_{\tilde{g}}={\langle \kappa_{1}E_{1},E_{1}\rangle }_{\tilde{g}}=\kappa_{1}$,

$h_{22}=h\left(E_{2},E_{2}\right)={\langle SE_{2},E_{2}\rangle }_{\tilde{g}}={\langle \kappa_{2}E_{2},E_{2}\rangle }_{\tilde{g}}=\kappa_{2}$,

$h_{12}=h\left(E_{1},E_{2}\right)={\langle SE_{1},E_{2}\rangle }_{\tilde{g}}={\langle \kappa_{1}E_{1},E_{2}\rangle }_{\tilde{g}}=0$,

$h_{21}=h\left(E_{2},E_{1}\right)={\langle SE_{2},E_{1}\rangle }_{\tilde{g}}={\langle \kappa_{2}E_{2},E_{1}\rangle }_{\tilde{g}}=0$.

Thus, Equation (A8) can be rewritten as $R_{m}\left(E_{1},E_{2},E_{1},E_{2}\right)={\tilde{R}}_{m}\left(E_{1},E_{2},E_{1},E_{2}\right)+\kappa_{1}\kappa_{2}$.

Rearranging and using skew-symmetry of the Riemann curvature tensor, we obtain the perceived curvature $\kappa_{1}\kappa_{2}$ of the submanifold at the point $q\in \;\left(\tilde{G},\tilde{g}\right)$ to be:

(A9) $$\kappa_{1}\kappa_{2}={\tilde{R}}_{m}\left(E_{1},E_{2},E_{2},E_{1}\right)-R_{m}\left(E_{1},E_{2},E_{2},E_{1}\right),$$

where ${\tilde{R}}_{m}\left(E_{1},E_{2},E_{2},E_{1}\right)$ is the Gaussian curvature of the submanifold $\left(\tilde{G},\tilde{g}\right)$ at $q\in \;\left(\tilde{G},\tilde{g}\right)$ and $R_{m}\left(E_{1},E_{2},E_{2},E_{1}\right)$ is the sectional curvature of the ambient perceived visual manifold (G,g) at q∈ (G,g) associated with the plane in $T_{q}G$ spanned by the two orthonormal vectors $E_{1}$ and $E_{2}$ tangent to the submanifold at that point. Both the Gaussian and sectional curvatures in Equation (A9) depend on the position q∈ (G,g) in the perceived visual manifold and on the orientation of the submanifold defined by the plane in (G,g) spanned by $E_{1}$ and $E_{2}$ tangent to the submanifold at $q\in \;\left(\tilde{G},\tilde{g}\right)$.
